# STEER: decoupling kinetics with Spatial-Temporal Explainable Expert model for RNA velocity inference

**DOI:** 10.1093/nsr/nwag199

**Published:** 2026-03-30

**Authors:** Zhiyuan Liu, Yaru Li, Dafei Wu, Weiwei Zhai, Liang Ma

**Affiliations:** State Key Laboratory of Animal Biodiversity Conservation and Integrated Pest Management, Institute of Zoology, Chinese Academy of Sciences, Beijing 100101, China; University of the Chinese Academy of Sciences, Beijing 100049, China; Department of Automation, Tsinghua University, Beijing 100084, China; State Key Laboratory of Animal Biodiversity Conservation and Integrated Pest Management, Institute of Zoology, Chinese Academy of Sciences, Beijing 100101, China; State Key Laboratory of Animal Biodiversity Conservation and Integrated Pest Management, Institute of Zoology, Chinese Academy of Sciences, Beijing 100101, China; University of the Chinese Academy of Sciences, Beijing 100049, China; Center for Excellence in Animal Evolution and Genetics, Chinese Academy of Sciences, Kunming 650223, China; State Key Laboratory of Animal Biodiversity Conservation and Integrated Pest Management, Institute of Zoology, Chinese Academy of Sciences, Beijing 100101, China; University of the Chinese Academy of Sciences, Beijing 100049, China

**Keywords:** spatial RNA velocity, kinetics disentangle, mixture of experts, graph attention network, spatial transcriptomics

## Abstract

RNA velocity provides a powerful scope for understanding cell state dynamics by modeling spliced and unspliced mRNA captured by single-cell or spatial transcriptomic technologies. However, prevailing methods relying on restrictive kinetic assumptions often fail in the presence of heterogeneous kinetic regimes, which is common in tissues of complex biological systems. These limitations hinder both accurate inference and interpretability, particularly in spatial contexts with kinetic mixing. Here, we present STEER (Spatial-Temporal Explainable Expert model for RNA-velocity inference), a flexible and interpretable framework that integrates spatially informed graph-attention auto-encoder with a kinetically guided mixture-of-experts architecture. STEER disentangles kinetically and/or spatially mixed populations by assigning cells to expert-defined regimes, and infers cell-gene-specific kinetic rates with cell-level latent time. Benchmarking STEER on synthetic and challenging real-world systems, demonstrates its robust performance and enhanced interpretability. Particularly, STEER reveals spatiotemporally complementary immunoregulatory programs at the maternal–fetal interface of mouse uterus. Overall, STEER provides a generalizable and explainable framework for decoding nuanced spatio‑temporal dynamics in complex tissues, offering insight into tissue morphogenesis, lineage specification, and tumor progression.

## INTRODUCTION

Complex biological systems are governed by multiple, often overlapping cellular processes that act simultaneously across time and space [1,2]. Advances in single-cell and spatial transcriptomics capture heterogeneous transcriptional states and, in spatial assays, provide tissue-level context [3–5], offering rich insights into molecular function, tissue development, and disease progression [6–8]. RNA velocity methods infer the directionality of cell-state changes by exploiting the kinetics of unspliced and spliced mRNA with a mechanistic model, thereby offering a principled window into cellular dynamics [9,10].

Despite its promise, current RNA-velocity methods face fundamental limitations in the presence of mixed kinetics [2,11–13] (that is, coexisting regimes with distinct kinetic laws for transcription, splicing, and degradation rates; parameters may vary by cell and gene within each law). Prevailing approaches often impose restrictive assumptions, including globally shared or time-invariant kinetic rates [9,14,15]; equilibrium or near-equilibrium behavior [9,16]; and/or a single kinetic regime shared by all populations [9,17–19] (that is, common kinetic law). In heterogeneous tissues, where distinct programs co-occur and switch sharply in a state and/or niche dependent manner [20–23], these assumptions conflate signals from multiple regimes, obscure lineage or context specific dynamics, and degrade the accuracy of velocity fields and latent-time estimates. Spatial mixing further compounds the problem because physical proximity does not imply kinetic uniformity; consequently, borrowing information across heterogeneous spatial neighborhoods blends cells with incompatible kinetics [24–26], reducing inference robustness and confounding regime-specific dynamics.

To address these limitations, we present STEER (Spatial-Temporal Explainable Expert model for RNA-velocity inference), a unified deep learning framework that disentangles mixed kinetics while leveraging spatial context. STEER integrates a spatially informed graph-attention auto-encoder with a kinetically guided mixture-of-experts (MoE) that partitions cells into locally coherent regimes. It learns cell level latent state and kinetic time, and cell-gene-specific transcription, splicing, and degradation rates. This piecewise formulation enables regime-level comparison and preserve gene-level interpretability. Across synthetic benchmarks and diverse biological systems, including hematopoiesis, neurodevelopment, placentation, and adult brain, we show that STEER dissects nuanced, co-occurring and potentially spatially intertwined dynamics, offering an interpretable and generalizable framework for spatio-temporal velocity modeling in complex tissues.

## RESULTS

### Overview of STEER

**Figure 1.**
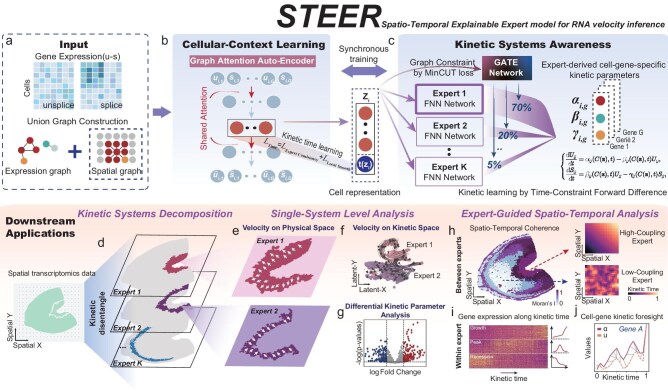
Overview of the STEER framework and its downstream applications. (a) For each single cell or spatial spot, unspliced (U) and spliced (S) read counts are concatenated into a node-feature vector. A unified graph is built by combining transcriptional similarity with spatial proximity (when available). (b) A GAAE projects nodes into latent space while reusing the attention matrix learned in the first graph attention layer, thereby preserving local neighborhood structure and reducing over-smoothing. The bottleneck embedding is passed through a kinetic-time encoder and combined with the inferred time to yield the final cell representation. (c) Latent cell representations and inferred kinetic time are softly routed to multiple expert networks via a gating module, with each expert representing a distinct kinetic regime. Cell-gene-specific transcription (α), splicing (β), and degradation (γ) rates are modeled via expert-conditioned kinetic functions. A graph-constrained MinCUT regularization promotes topological coherence of expert assignments, and kinetic parameters are optimized using a temporally constrained forward-difference loss that restricts supervision to forward neighbors along inferred progression. (d) Expert assignments separate overlapping dynamical systems and partition spatially intermixed trends into distinct kinetic layers. (e–g) Based on inferred kinetic regimes, STEER supports analyses at the level of individual experts. (e) RNA-velocity vectors mapped onto the physical coordinates reveal system-specific flow patterns *in situ*. (f) Projection of the same velocity vectors into the learned kinetic embedding reveals trajectories in latent kinetic space. (g) Cell-gene specific kinetic parameters enable differential analysis between experts. (h–j) Expert-guided spatiotemporal analysis. STEER also provides insights into dynamics both between and within experts. (h) Spatial autocorrelation of expert-specific latent time quantifies the degree of spatiotemporal coherence for each expert; with examples of high and low coupling. (i) Ordering cells by expert-specific latent time identifies time-variable genes associated with progression within each kinetic regime. (j) Dynamic time warping alignment of transcription rate α and unspliced counts shows that changes in α can precede changes in unspliced abundance, providing an early kinetic indicator of future cell-state transitions.

STEER is a kinetically informed deep learning framework that combines a graph attention autoencoder (GAAE) with a MoE architecture to infer gene- and cell-specific transcriptional kinetics from single-cell or spatial transcriptomic data. The model incorporates both unspliced and spliced mRNA abundances and a unified cell graph integrating expression similarity and spatial proximity (Fig. 1a; see Methods). The GAAE encodes this graph into a context-aware latent representation ${{\bf z}}$ (Fig. 1b), while a latent kinetic time variable *t* is jointly inferred and regularized to vary smoothly along the latent representation space.

To capture transcriptional heterogeneity arising from spatial context, temporal progression, or divergent regulatory programs, STEER adopts a generalized transcription kinetics model with regime- and time-dependent parameters:


\begin{eqnarray*}
\left\{ {\begin{array}{@{}*{2}{l}@{}} {\displaystyle\frac{{d{U}_g}}{{dt}}}&{ = {\alpha }_g ( {C ( {{\bf z}} ),\!t} ) - {\beta }_g ( {C ( {{\bf z}} ),\!t} ){U}_g,}\\ {\displaystyle\frac{{d{S}_g}}{{dt}}}&{ = {\beta }_g ( {C ( {{\bf z}} ),\!t} ){U}_g - {\gamma }_g ( {C ( {{\bf z}}),\!t} ){S}_g,} \end{array}} \right.
\end{eqnarray*}

where ${U}_g$ and ${S}_g$ denote the unspliced and spliced abundances of gene *g*, and $C( {{\bf z}} )\in \{ {1,2,\ldots,K} \}$ denotes a discrete kinetic regime assignment determined by the latent state. STEER implements $C( {{\bf z}} )$ via an MoE module with *K* expert networks, each defining a regime-level kinetic function that governs the cell–gene-specific rates $( {\alpha ,\beta ,\gamma } )$. The number of experts is selected using an entropy-elbow criterion (see Supplementary Note). A soft gating network assigns each cell a distribution over experts based on its latent state and time, and a MinCUT-based regularization stabilizes this routing by encouraging topological consistency while preserving sharp regime boundaries along the latent representation space (Fig. 1c).

The training proceeds in three stages under a self-supervised objective that combines graph reconstruction, temporal regularization (directionality and smoothness), and a dynamical consistency loss that ties the learned kinetics to locally observed transcriptional changes along latent time (see Methods for details). The initialization stage pretrains the GAAE to initiate a context-aware embedding from highly variable genes. The second stage refines the latent space by introducing temporal regularization. The final stage jointly optimizes expert routing and MoE kinetics under the full objective (including MinCUT and the dynamical loss) to learn regime-specific transcriptional dynamics.

After training, STEER disentangles mixed dynamical programs and yields interpretable outputs, including regime assignments, latent kinetic times, gene- and cell-specific kinetic parameters, and velocity vectors. These outputs support downstream analyses such as regime-resolved velocity visualization in spatial and latent spaces, expert-aware kinetic profiling of fate-priming signals, and time-variable gene discovery guided by latent time (Fig. 1d–j).

### STEER decomposes multi-regime kinetics and yields robust velocity across single-cell and spatial settings

We evaluated STEER’s ability to disentangle heterogeneous transcriptional kinetics through benchmarks on simulations and real datasets spanning both single-cell and spatial transcriptomics settings.

**Figure 2.**
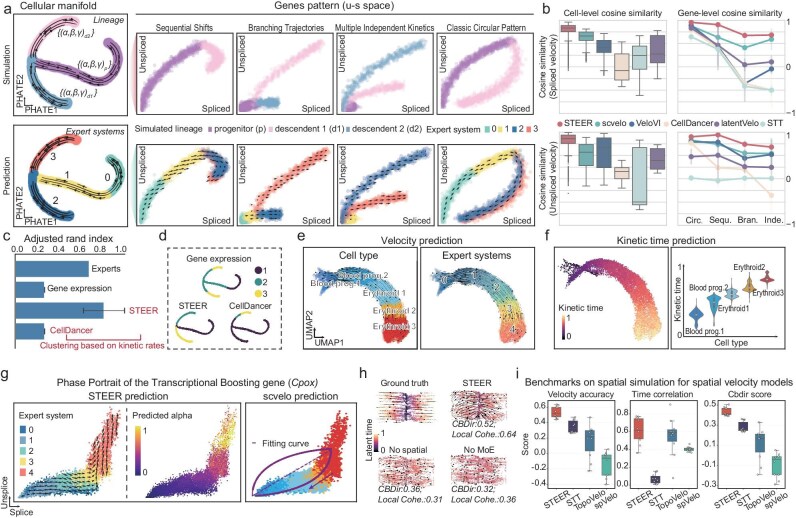
STEER decomposes multi-regime kinetics and yields robust velocity across single-cell and spatial settings. (a) Top: Simulation design showing ground-truth velocity in PHATE space (left) and representative unspliced–spliced phase portraits under four synthetic kinetic patterns (right; see Note S7), with cells colored by simulated kinetic regimes. Bottom: STEER-inferred velocity for the same cells at the cell and gene levels, colored by predicted expert assignment. (b) Boxplots show cosine similarity between predicted and ground-truth cell-level velocity (left), and line plots show gene-level velocity cosine similarity for the gene patterns in (a) (right). Points and error bars indicate mean ± s.d. (c) Adjusted Rand index between lineage labels and clusters defined by expert assignment, K-means on gene expression, or K-means on predicted kinetic rates (α, β, and γ). Bars show mean and error bars show s.d. across five clustering repeats. (d) PHATE embedding colored by K-means clusters based on gene expression, CellDancer-predicted kinetic parameters, or STEER expert assignment. (e) STEER-inferred velocity in single-cell data, with cells colored by cell type (left) or expert assignment (right). (f) Latent-time predictions shown in UMAP space (left) and summarized by cell type (right). (g) Velocity dynamics of the transcriptionally boosted gene Cpox. Left: STEER inference colored by Expert System. Middle: Predicted transcription rate. Right: Comparison with scVelo. (h) Ablation analysis in spatial simulations, showing ground-truth velocity and latent time, followed by results from No MoE, No Spatial, and full STEER models. CBDir and local coherence scores are shown below each panel. (i) Performance benchmarking on spatial simulations. Boxplots summarize velocity accuracy, latent-time correlation, and CBDir scores across 10 replicates covering bilinear and multi-radial dynamical scenarios. In panels (b, f, and i), boxplots show the median and interquartile range, with whiskers extending to 1.5 × IQR. Time values in panels (f, h) were min–max normalized. Data in panels (a–d) are based on 2000 cells; those in panels (e–g) are based on 9815 cells. Abbreviations: MoE, mixture of experts; CBDir, cross-boundary directionality; Local Cohe., local coherence; Blood Prog., blood progenitors. Circ., classic circular pattern; Sequ., sequential shifts; Bran., branching trajectories; and Inde., multiple independent kinetics.

We first used a synthetic data bifurcating process. It constitutes 1000 simulated genes with known velocities and latent time, across four major kinetic regimes (Methods). Among all methods, STEER yielded velocity estimates that most closely matched the ground-truth dynamics (Fig. 2a and b; Figs S1–S2). Its expert-based architecture successfully decomposed lineage-specific kinetic regimes, enabling accurate inference of gene-level kinetics in all four patterns (Fig. 2a and b; Fig. S1d). In contrast, alternative methods performed well mainly on genes with circular pattern on the u–s space, which resembled the classic almond shape [14], but showed reduced accuracy in other patterns, especially patterns with explicit piecewise kinetics (for example, branching, independent) (Fig. 2b). In addition, STEER produced coherent kinetic time estimates (Fig. S3) and provided cell- and gene-specific estimates of transcription, splicing and degradation rates ($\alpha $, $\beta $, and $\gamma $). Clustering by STEER inferred kinetic rates yielded finer segmentation of dynamic regimes than clustering by cellDancer derived rates or by raw expression profiles (Fig. 2c and d).

We next assessed STEER on a mouse erythroid maturation single-cell dataset, a classic system that has been reported to exhibit rapid, state-dependent kinetic shifts, including late-stage transcriptional boosting [13,27]. STEER reconstructed the expected maturation-directed velocity, with its inferred latent-time ordering aligned with annotated stages (Fig. 2e and f). Importantly, STEER recovered the late-stage transcriptional burst by assigning it to specific experts (for example, Experts 3 and 4). In particular, STEER identified elevated transcription rates ($\alpha $) for boosting genes such as *Cpox* and *Hba-x* (Fig. 2g; Fig. S4b–d; Note S8). In contrast, methods assuming global kinetics can fail to capture these regime shifts and may yield artificial backflow of velocity toward progenitors [2] (Fig. S4a). We further demonstrated STEER’s robust performance in resolving multi-lineage trajectories in more complex real biological systems across additional single-cell datasets [9,27,28], including whole-mouse gastrulation and dentate gyrus development (Note S10; Figs S5–S7).

Beyond single-cell settings, STEER integrates multi-regime kinetic decomposition with spatial neighborhood information when available. Using spatial simulations with known ground truth [29], we performed ablations to disentangle the respective contributions of the MoE module and the spatial graph. Removing the MoE collapses STEER to a single-regime fit, abolishing regime decomposition and reducing directional consistency with the ground-truth dynamics (Fig. 2h). Conversely, ablating the spatial graph diminishes the local coherence of the inferred vector field (Fig. 2h). Overall, the full model achieves the closest agreement with the ground truth, indicating that the regime decomposition and spatial coupling provide complementary benefits. Benchmarking STEER against state-of-the-art spatial velocity inference approaches [16,29,30], we observed consistent improvements in velocity accuracy, latent-time correlation, and CBDir scores across spatial simulations real spatial transcriptomic datasets spanning multiple platforms, including Stereo-seq, Visium, Slide-seqV2, etc. (Fig. 2i; Figs S6 and S8).

Collectively, these benchmarks indicate that STEER can disentangle co-occurring kinetic regimes, thereby improving the accuracy of velocity and latent-time estimates across both single-cell and spatial settings. We further show that STEER remains robust when trained on downsampled (sequencing) read depth or number of cells. It also shows promising generalizability to intermediate dynamics not observed during training (Figs S9–S11).

### STEER resolves regime-specific early kinetics underlying lineage commitment

Lineage commitment is often triggered by subtle shifts in transcriptional regulation that precede apparent changes in expression. The kinetics-informed decomposition allows STEER to distinguish such lineage priming trend with early rate variation. We validate this in both neurodevelopmental and hematopoietic systems.

**Figure 3.**
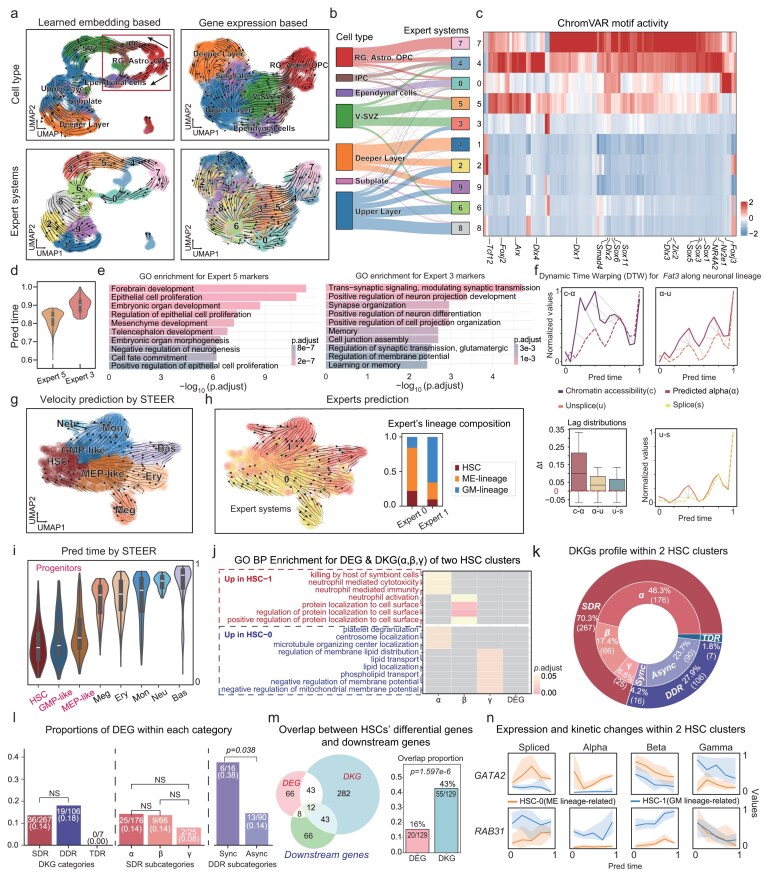
STEER resolves early-stage kinetics underlying lineage specification. (a) RNA velocity projections in UMAP space, annotated by cell type (top) and STEER expert systems (bottom). Left: projections based on STEER-derived latent embedding; right: projections based on gene expression. Black arrows highlight two cell-fates trajectories. (b) Sankey diagram illustrating the correspondence between cell type annotations and expert assignments. (c) Heatmap of average *z*-score normalized ChromVAR motif deviation scores for each expert. Motifs were mapped to the mm10 genome using the JASPAR2020 database. (d) Violin plots of STEER-inferred kinetic time for the Experts 5 and 3. (e) GO enrichment analysis of genes differentially expressed between Experts 5 and 3. (f) Dynamic time warping alignment of transcriptional features for Fat3 along the neuronal lineage (excluding Expert 0). Pairwise alignments are shown for chromatin accessibility c vs. α (left), α vs. unspliced counts (u, middle), and u vs. spliced counts (s, right). In each comparison, the second feature is shown as a dashed line. Gray dotted lines indicate aligned time points across 15 equal-sized bins; lines trace average values per bin. (g) RNA velocity projections in UMAP space inferred by STEER. (h) RNA velocity projection colored by expert systems, with stacked bar plot summarizing lineage contributions to each expert, including HSC, ME lineage (MEP-like, Meg, Ery), and GM lineage (GMP-like, Mon, Neu, Bas). (i) Violin plots of STEER-inferred latent time across cell types; progenitor populations are indicated. (j) GO enrichment analysis of differentially expressed genes and differential kinetic genes (α, β, and γ) between two experts within the HSC population. (k) Double doughnut chart showing the composition of DKGs in two HSC expert groups, including single differential rate (SDR), double differential rate (DDR), and triple differential rate (TDR) categories, with DDR further divided into synchronous and asynchronous patterns. (l) Bar plots showing the proportion of DEGs within each DKG category. The left panel compares DEG enrichment between SDR and DDR (TDR excluded due to sparsity). The middle panel further subdivides SDR into differential α, β, and γ categories. The right panel contrasts Sync vs. Async patterns within DDR. (m) Venn diagram showing overlap among DEGs, DKGs, and downstream DEGs (from MEP-like vs. GMP-like comparison), with bar plots quantifying the overlaps. (n) Temporal trajectories of gene expression and kinetic parameters for GATA2 (top) and RAB31 (bottom) across STEER-inferred time within the HSC population. Cells were grouped into five equal time bins; lines represent median values and shaded regions indicate interquartile ranges. All statistical comparisons were performed using two-proportion *z*-tests (two-tailed), NS: *P* > 0.1.

We first applied STEER to an embryonic day 18 mouse brain data assayed with 10× Multiome, which provides paired scRNA and scATAC profiles. Using only spliced and unspliced counts, STEER recovered velocity fields and latent time that faithfully recapitulated the neurodevelopmental progression, which corroborated with declined overall chromatin accessibility across genes [31–33] (Fig. 3a; Fig. S12).

STEER disentangled two trajectories, as revealed only by its learned kinetics-informed embedding, rooted from radial glia (RG) cell population (Fig. 3a). A neuronal trajectory that proceeds through intermediate progenitor cells to cortical neurons [34,35], and an ependymal trajectory which culminates in ependymal cells that provide structural and regulatory support for neurogenesis [36,37]. This lineage segregation was clearly captured by the separation of Expert 4 (neuronal) and Expert 0 (ependymal) from the RG-associated Expert 7 (Fig. 3a, left; Fig. S13). Notably, the experts tend to stratify cells more by kinetic rate changes than by expression differences. This is supported by the markedly sharper transitions in relative kinetic distance than in gene expression distance at expert boundaries (Fig. S14; Note S14).

Along the neuronal trajectory, STEER further delineated several expert-defined sub-states (Fig. 3b), with early stage experts showing high ChromVAR motif activity across a broad set of transcription factors, including regulators implicated in neurogenesis and stem-cell maintenance, whereas later-stage experts focused on a limited set of transcription factors (Fig. 3c; Figs S12b and S15), consistent with progressively restricted regulatory programs during neuronal maturation [31]. Particularly, Expert 5 and Expert 3 decoupled cells in ventricular-subventricular zone (Fig. 3b). Consistent with their positions along latent time, Expert 5 was linked to early neurogenic programs, whereas Expert 3 was linked to synaptogenesis and neuronal differentiation (Fig. 3c–e; Fig. S16). Further investigation of gene-level kinetics across these expert-defined regimes revealed the cascade of cis-regulatory dynamics, where chromatin opening preceded transcriptional activation (that is, elevated $\alpha $), unspliced transcripts accumulation, and mature mRNA production (Fig. 3f; Figs S17 and S18).

Next, we apply STEER to more challenging human haematopoiesis data profiled by scNT-seq. This protocol couples metabolic labelling with scRNA-seq, providing an independent temporal yard-stick [38]. Without leveraging such temporal information, conventional methods can hardly recover the correct hierarchy of hematopoiesis [2,38] (Fig. S19a). In contrast, STEER successfully resolved these dynamics with solely transcriptomic data and recapitulated the differentiation from hematopoietic stem cells (HSCs) toward granulocyte-monocyte (GM) lineage and megakaryocyte-erythroid lineage with separate experts (Fig. 3g–i; Fig. S19b, c). Expert 0 captured the trajectory from HSCs through MEP-like intermediates to megakaryocytes and erythrocytes, while Expert 1 traced the path from HSCs through GMP-like to neutrophil, monocyte and basophil (Fig. 3g–i; Fig. S19c).

Focusing on the early stem cell population, the two experts partitioned the HSCs into subpopulations, which we termed HSC-0 and HSC-1. These subpopulations presented 129 differential expression genes (DEGs) but found no significantly enriched functional pathways (Fig. 3j, right panel; Fig. S19d; Table S1). On the contrary, genes with differential kinetic rates (referred as DKGs, see Methods for details) were strongly enriched for lineage-relevant programs such as platelet degranulation for ME priming and immune response for GM priming (Fig. 3j; Fig. S19e). In the 380 identified DKGs, only 55 genes overlapped with DEGs (Fig. S19f, left). The overlapping genes constitute of 43% of DEGs but only 14% of DKGs, indicating that kinetics exposes regulatory bias in advance to detectable expression changes (Fig. S19f, right). Further partition the DKGs according to the number and combination of differential kinetic parameters (see Methods), we found that genes with coordinated synergistic rate shifts (specifically double differential rates combining increased transcription ($\uparrow \alpha$) with decreased degradation ($\downarrow\! \gamma$), or vice versa) displayed significantly greater proportion overlapping with DEGs (Fig. 3k and l right panel), indicating that coordinated transcription-degradation modulation amplifies expression differences. Notably, such genes represented only a small fraction of all DKGs (Fig. 3k), underscoring the unique capacity of kinetic rate analyses to capture biological nuances that traditional DEGs alone may overlook.

Kinetic decomposition enabled STEER to identify early differential signals consistent with emerging lineage bias. Roughly half of the DEGs that later distinguish MEP-like from GMP-like cells already show differential signals (expression or kinetics) in HSCs (Fig. 3m). *GATA2*, the canonical ME regulator [39], was consistently identified by both expression- and kinetics-based tests at the progenitor stage and again among downstream DEGs. In contrast, *RAB31*, a coordinator of endolysosomal transport in monocyte-lineage cells [40], emerged solely as a DKG: its rates shifted markedly without significant expression differences (Fig. 3n; Table S1). These cases exemplify how STEER’s regime-specific decomposition detects fate-biasing kinetics that precede transcriptional divergence.

Taken together, these findings demonstrate STEER’s advantage in revealing early regulatory events relating to cell-fate specification, which insights that can hardly be accessed by conventional velocity or DEG analysis.

### STEER decouples confounding kinetic trends in mouse placentation spatial data

**Figure 4.**
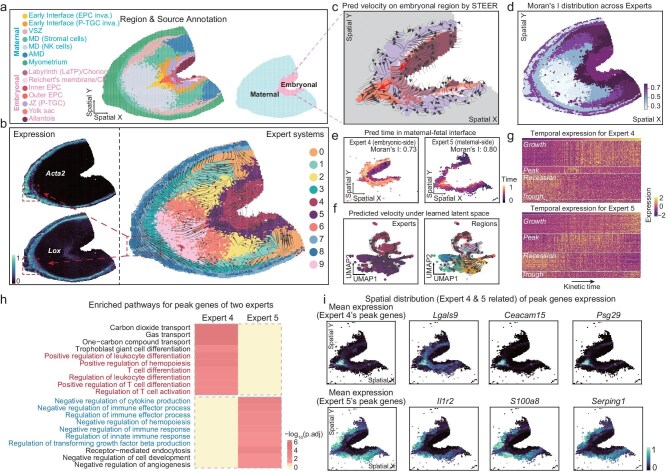
STEER reveals spatio-temporal dynamics in mouse placental spatial transcriptomics. (a) Spatial annotations of tissue regions and sources (embryonic vs. maternal) for each spot. Region labels indicate embryonic or maternal origin for each spot. (b) STEER-inferred RNA velocity vectors projected onto spatial coordinates, colored by expert assignment (right). Normalized expression patterns of Acta2 (contractile cytoskeleton) and Lox (ECM remodeling) (left) highlight spatial concordance with expert assignment. Red rectangles and arrows indicate corresponding structures across gene expression and expert distribution. (c) RNA velocity fields predicted by STEER in the embryonic compartment. Arrows indicate directional flows from inner to outer ectoplacental cone (EPC) and from allantois to chorion. (d) Spatial distribution of Moran’s I values for kinetic time across experts, revealing varying degrees of spatiotemporal coupling. (e) Predicted kinetic time for experts at the maternal-fetal interface (Expert 4: embryonic side; Expert 5: maternal side). Spatial autocorrelation of these time values is quantified by Moran’s I. (f) Predicted RNA velocity in the learned latent space for Experts 4 and 5, colored by expert assignment (left) and region annotation (right). Annotations are as in (a) and (b). (g) Time-variable genes identified for Experts 4 and 5, categorized into Growth, Peak, Recession, and Trough patterns. Columns represent cells; rows represent genes; color indicates z-score normalized expression. (h) Pathway enrichment of Peak genes from Experts 4 and 5. Bar color indicates −log10 adjusted *P*-values (Benjamini–Hochberg correction). Highlighted pathways reflect immune specialization in Expert 4 and immune suppression in Expert 5. (i) Spatial expression of Peak genes from Experts 4 (top row) and 5 (bottom row). Leftmost panels show mean expression across all Peak genes per expert. The remaining panels show representative genes: Lgals9, Psg29, and Ceacam15 for Expert 4; Il1r2, Serping1, and S100a8 for Expert 5. The plotted spatial domain corresponding to the regions occupied by Expert 4 and Expert 5. Panels (a, b, and d) include 13 258 spatial transcriptomic spots. Abbreviations: EPC, ectoplacental cone; P-TGC, parietal trophoblast giant cells; VSZ, vascular sinuses zone; MD, mesometrial decidua; AMD, anti-mesometrial decidua; LaTP, labyrinth trophoblast progenitor; CP, chorionic plate; and JZ, junctional zone.

The development of multicellular organisms is orchestrated by cellular processes evolving both temporally and spatially. However, divergent kinetic trends in neighboring regions pose a major challenge for single-regime methods that infer spatial trajectories. To assess STEER’s capability to resolve such intertwined dynamics, we applied it to high-resolution spatial transcriptomics data of mouse uterine tissue at embryonic day 8.5, generated by Stereo-seq [41]. This data encompasses both maternal and embryonic compartments and was originally annotated at bin size 50 (Fig. 4a).

The multi-regime decomposition allows STEER to reveal complex spatio-temporal dynamics, and more importantly, the pivotal embryonic events that aligned the developmental stage (Fig. 4b and c; Fig. S20a and b). In particular, it resolved the migration of allantois to chorion within the embryonic compartment [41], and characterized the invasion of inner ectoplacental cone (EPC) to outer EPC toward the maternal decidua boundary (Fig. 4c). Such events can be missed or only partially detected by single-regime methods (for example, scVelo) with manual segmentation of the embryonic region in advance (Fig. S21a–d). The respective contributions of model components to this observation are further examined by ablation analyses (Fig. S22c). In addition, these dynamics remain consistent across multiple binning resolutions (Fig. S23).

The experts identified by STEER delineated continuous spatial compartments in this tissue with varied spatio-temporal coherence (Fig. 4b and d; see Methods). Experts covering the mesometrial decidua (MD) region (Experts 6 and 9), enriched for high migratory NK cells, exhibited the lowest spatial coherence (Fig. 4d; Fig. S24a). We observed high spatio-temporal coherence on experts located surrounding MD region, which mainly cover the maternal-fetal (Experts 4 and 5) and myometrium-decidua (Expert 7) interfaces (Fig. 4d; Fig. S24a). Expert 7 marking the remodeling zone within myometrium [42] (for example, *Lox, Vim*), which distinguished from Expert 8 that defined the supportive smooth-muscle belt [43] (for example, *Acta2, Cnn1*) (Fig. 4b; Fig. S20c). Experts 4 and 5, situated on opposite sides of the maternal-fetal interface. Interestingly, they revealed antagonistic spatial dynamics with velocity fields converging towards the early interface (Fig. 4a, e and f; Fig. S20d).

Given the counter-directional dynamics, Experts 4 and 5 provided an opportunity to unlock the underlying molecular programs at the maternal-fetal interface. We applied TDEseq algorithm [44] to identify temporally dynamic genes within each expert, categorizing the identified genes into Growth, Peak, Recession, and Trough patterns based on expression dynamics along the inferred kinetic time (Fig. 4g). Pathway enrichment analyses revealed that these time-variable genes were associated with interface development, tissue remodeling, inflammatory responses, and cell migration (Fig. 4h; Fig. S24b).

Among them, Peak-pattern genes highlighted complementary immune-regulatory programs across the interface (Fig. 4h). In Expert 4, Peak genes exhibited maximal expression in the outer EPC region of the embryonic compartment (Fig. 4a and i top), implicating embryonic signals promote maternal immune cells toward specialized, tolerogenic states (for example, regulation of T cell activation and hematopoiesis) (Fig. 4h). Conversely, in Expert 5, Peak genes peaked on the maternal decidual side (Fig. 4a and i bottom) and were enriched for pathways suppressing excessive immune responses (for example, negative regulation of cytokine production) (Fig. 4h). These observations reflect a bidirectional immunomodulatory dialogue at the maternal-fetal interface: the embryo compartment drives immune specialization, whereas the maternal compartment imposes essential inhibitory checkpoints. Such complementary immunoregulatory programs establish a fine balance conducive to a stable pregnancy.

In summary, STEER’s MoE framework disentangles complex spatio-temporal dynamics without prior anatomical partitioning, enabling accurate velocity inference across spatially contiguous yet kinetically divergent regions. By decomposing overlapping spatial processes, STEER not only reconstructs developmentally relevant trajectory fields but also uncovers molecular programs that coordinate spatial organization.

### STEER generalizes regime-aware spatial dynamics in non-developmental tissues

Having focused on developmental settings above, we next asked whether steer can be extended to reveal interpretable dynamics in non-developmental tissues with strong spatial heterogeneity.

We first analyzed two human oral squamous cell carcinoma samples (OSCC) generated by 10× Visium spatial transcriptomics, where spots are annotated as tumor core, transitory, and invasive edge regions [45]. Since the two sample slices are from independent patients, we train steer on one slice (training slice) and apply it to the other slice (transfer slice) in a zero-shot transfer manner as validation.

**Figure 5.**
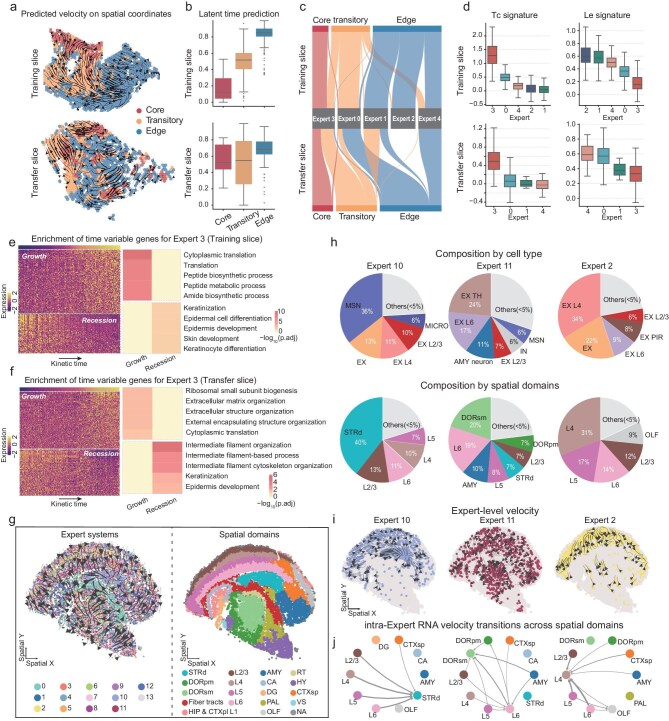
STEER generalizes regime-aware spatial dynamics across non-developmental tissues. (a) Predicted RNA velocity vector projected onto spatial coordinates for the training (top) and transfer (bottom) slices. (b) Boxplots of STEER-inferred latent time across Core, Transitory, and Edge regions. (c) Sankey diagram visualizing the consistent mapping between histological spatial domains and STEER expert assignments across both slices. (d) Validation of expert identity using established Tc and Le gene signatures. (e, f) Temporal dynamics of Expert 3 (Tumor Core) in training slice (e) and transfer slice (f). Left: Heatmap of time-variable genes ordered by latent time, highlighting Growth and Recession programs. Right: Significant pathways identified in functional enrichment analysis for time variable genes. (g) Left: Aggregate RNA velocity field predicted by STEER for 11 854 spatially resolved cells, colors by expert assignment. Right: Spatial domain annotations of individual cells, with colors indicating anatomically defined regions. (h) Pie charts showing the composition of cell types (top) and spatial domains (bottom) for the three identified kinetic experts (Experts 10, 11, and 2). Minor populations (< 5%) were grouped as ‘Others’. (i) Expert-specific RNA velocity fields, revealing distinct dynamic streams. (j) Network plots showing intra-expert RNA velocity transitions across spatial domains, with each network directly corresponding to the expert shown above.

In the training slice, steer recovered a coherent ordering from the tumor core through a transitional zone toward the invasive edge in both the spatial velocity field and the inferred kinetic time (Fig. 5a and b top). Expert assignment further decomposed this organization into distinct regimes (Fig. 5c top). In particular, Expert 3 dominated the tumor core region and extended into adjacent transitory region. It showed the highest tumor-core (TC) signature and lowest leading-edge (LE) signatures [45] among other experts (Fig. 5d). Along Expert 3 specific kinetic time, increasing genes were enriched for translation and biosynthetic programs, whereas decreasing genes were enriched for squamous epithelial differentiation and keratinization (Fig. 5e). This pattern is consistent with de-differentiation accompanied by elevated biosynthetic activity, which may precede or accompany transitions toward more invasive programs.

By directly transfer the trained model to the held-out slice without fine-tuning, steer consistently recovered the core-to-edge ordering in both the velocity field and kinetic time (Fig. 5a and b bottom). Despite a more fragmented spatial distribution of the tumor core, they were consistently assigned to Expert 3 (Fig. 5c and d bottom). Its time-variable genes recapitulated concordant functional themes as in training slice, with increasing genes enriched for ribosome/translation-related processes (with additional enrichment for extracellular matrix organization) and decreasing genes enriched for intermediate filament organization and keratinization/epidermis-development terms (Fig. 5f). The consistent results demonstrate that STEER can resolve non-development dynamics in cancer and can generalize across patients.

We next applied steer to Stereo-seq data from an adult mouse coronal hemibrain [46]. The aggregate velocity field showed a dominant cortex-to-subcortex trend across the sampled tissue plane (Fig. 5g). Unlike the developmental settings in Fig. 4, expert assignments in the adult brain were not organized into contiguous spatial domains but were instead spatially intermingled (Fig. 5g; Fig. S25a and c). We characterized each expert by its cell-type and spatial-domain composition together with marker-gene signatures (Fig. 5h; Fig. S25a and b), and summarized expert-specific time-variable genes along kinetic time (Fig. S26; Table S2).

Through kinetic decomposition, steer separated this composite field into expert-specific velocity components with coherent directionality across major cortical and subcortical structures (Fig. 5i and j; Fig. S25a–d; Supplementary Methods). For example, Expert 10 captured a convergent component from broad cortical regions toward the dorsal striatum (STR_d_); Expert 11 captured a cortex-thalamus component spanning upper layers into layer 6 (L6) and extending toward thalamic nuclei (DOR_sm_ and DOR_pm_); and Expert 2 emphasized an intracortical component with streamlines accumulating toward layer 4 (L4). These examples illustrate how expert routing can disentangle spatially intertwined components of the velocity field in mature tissue. The resulting components form structured spatial patterns consistent with prior reports of region- and layer-associated transcriptomic organization in adult brain tissue [47,48], and can be viewed as transcriptome-derived kinetic programs mapped onto tissue coordinates.

Collectively, these results indicate that steer can decompose composite spatial dynamics into expert-specific kinetic components and associated gene programs in both heterogeneous tumors and mature brain tissue.

## DISCUSSION

In this study, we introduce STEER, a deep learning framework that unifies a spatially informed GAAE with a kinetically guided MoE to infer RNA velocity, cell‑specific kinetic parameters, and a globally coherent latent time from single-cell or spatial transcriptomic data. Existing approaches either rely on globally shared or weakly factorized kinetic architectures, which can suffer from parameter interference in multi-regime systems, or incorporate spatial context only post hoc, limiting their ability to resolve co-localized but kinetically divergent programs. In contrast, STEER partitions the latent representation space into discrete expert-defined regimes while preserving global temporal continuity. This regime-aware design not only yields more accurate velocity vectors in synthetic benchmarks, but also correctly oriented trajectories in erythropoiesis and hippocampal neurogenesis. By integrating spatial information, STEER disentangles spatially antagonistic maternal-fetal signals and resolves transcriptional gradients in complex tissues beyond developmental scenarios.

A central innovation of STEER lies in its MoE architecture, which addresses a fundamental limitation of simple or single-network models when distinct transcriptional programs coexist. By routing cells to specialized expert networks, STEER learns piecewise kinetic surfaces ($\alpha $, $\beta $, $\gamma $) which yield interpretable and biologically coherent regimes. In synthetic data featuring a bifurcating trajectory, STEER’s experts aligned with the ground-truth lineage and recovered of branch-specific induction and repression patterns that baseline models averaged out. In human hematopoiesis, this expert-based decomposition revealed early fate-priming signatures in HSC subpopulations and recapitulated transcriptional boosts in canonical erythroid genes. Analysis integrates with chromatin accessibility data revealed regime level cascade of temporal change where chromatin opening precede the increases in $\alpha $, thus linking early epigenomic remodeling to transcriptional activation and lineage commitment.

The application of graph attention enables STEER to emphasize informative neighbors and suppress noisy or poorly connected cells, which yielding smooth and coherent representation with precise latent time estimates even under sparse sampling. By constructing a union graph over expression similarity and spatial proximity, the attention-based weighting allow STEER to adaptively select relevant neighbors within a biologically informed context. This integration provides a flexible search space to mitigate spatial heterogeneity and to enforcing local continuity in the latent representation space, which enhance velocity estimation accuracy.

The GAAE–MoE integration is especially important for spatial transcriptomics, where transcriptionally divergent cell types can co-occur in physical proximity and violate local kinetic homogeneity assumptions [19,29]. STEER addresses this by combining a spatially informed graph with gated expert assignments, enabling it to disentangle overlapping programs and resolve counter-directional flows within the same tissue domain. At the maternal-fetal interface, STEER’s experts autonomously partitioned embryonic and decidual compartments and preserved consistent vector directions across boundaries, revealing coordinated smooth-muscle remodeling, trophoblast invasion, and complementary immunoregulatory dynamics. In contrast, baseline methods often required manual segmentation or produced inconsistent local directions under kinetic mixing. As an additional mature-tissue example, applying STEER to adult mouse hemibrain spatial transcriptomics likewise revealed expert-specific spatial gradients organized across major anatomical domains. Together, these results highlight how regime-aware decomposition can improve interpretability in anatomically complex, spatially heterogeneous systems.

More generally, in spatial transcriptomics the inferred velocity field should be interpreted primarily as a transcriptome-derived dynamical signal defined on cells/spots in tissue coordinates. Spatial velocity therefore often manifests as gradients of time-variable gene programs across anatomical domains, rather than literal displacement of the measured units. In developmental or remodeling settings, these gradients may co-occur with coordinated cell rearrangements and migration, but such correspondence is context-dependent and should not be assumed in mature tissues [2,49]. Within this framing, steer’s MoE provides a regime-aware decomposition of composite spatial dynamics, separating co-occurring kinetic programs into expert-specific components and enabling interpretation through cell-type composition, marker-gene signatures, and time-dependent gene modules.

Together, our findings position STEER as a regime-aware framework for studying heterogeneous cellular dynamics. By modeling transcriptional kinetics as a mixture of expert-defined regimes, STEER enables joint inference of velocity, latent time, and kinetic decomposition within spatially and temporally complex systems. Practically, the granularity of regime decomposition is constrained by sequencing depth, per-regime sample size, and computational budget; when *K* is limited, experts may capture higher-level macro-regimes. In addition, STEER assumes the availability of spliced and unspliced measurements and is applicable under data-quality levels observed in commonly used spatial transcriptomics platforms (Note S15). In spatial assays where splicing is not directly measured, upstream recovery of U/S layers (for example, as addressed by SIRV [50]) would be required prior to applying regime-aware kinetic modeling. Future extensions incorporating multi-omics priors or hierarchical expert routing may further refine regime resolution and extend this framework toward increasingly complex biological systems.

## MATERIALS AND METHODS

### Model design

STEER is a deep learning framework for RNA velocity inference that integrates a spatially informed GAAE and a kinetically guided MoE model to estimate cell- and gene-specific transcriptional kinetics. The model takes as input both unspliced and spliced transcript counts, together with a unified cell-cell graph constructed from transcriptional similarity and, when available, spatial proximity. The GAAE learns a low-dimensional embedding of cell states that preserves local context. A continuous latent-time variable is learned as a mapping from the latent state and is regularized to align with local transcriptional tendencies. To capture kinetic heterogeneity, a graph-constrained gated MoE module assigns cells to a set of discrete transcriptional regimes, each modeled by a dedicated expert network. This architecture enables flexible and interpretable modeling of complex, multi-regime transcriptional dynamics. Detailed model formulation and equations are provided in the Supplementary Methods.

### Training strategy

STEER was trained in three sequential stages. First, an initial GAAE was trained on highly variable genes to obtain a coarse latent representation and unsupervised cell grouping. These initial groups captured global transcriptional structure and were used to remove uninformative genes, infer local transcriptional tendencies, and provide a coarse prior for subsequent expert assignment. Second, the GAAE was retrained on the selected gene set with kinetic-time inference and temporal regularization enabled, producing a temporally coherent and spatially informed latent embedding that served as initialization for dynamical learning. Third, by activating the MoE module, the full model was trained end-to-end to jointly optimize expert routing, cell- and gene-specific kinetic parameters, and velocity consistency across temporally ordered neighborhoods. Detailed implementation descriptions of the temporal regularization, local transcriptional tendency inference, loss formulations and optimization strategies are provided in the Supplementary Methods.

### Benchmarking datasets

We evaluated STEER on both simulated and real datasets from single-cell and spatial transcriptomics. Simulations were designed to represent multiple kinetic regimes, including monotonic, branching, independent, and circular dynamics, and we additionally adopted the spatial simulation framework in TopoVelo for benchmarking under spatial settings. Real datasets included multiple single-cell and spatial transcriptomic platforms spanning developmental and non-developmental scenarios. Dataset details corresponding preprocessing procedures are described in the Supplementary Methods.

### Evaluation metrics

The performance of STEER was assessed using multiple complementary metrics covering velocity accuracy, temporal ordering, spatial consistency and spatiotemporal coherence. These included cosine similarity between predicted and ground-truth velocities, latent-time correlation, cross-boundary directionality (CBDir), local coherence and Moran’s I. Detailed definitions and implementation of all evaluation metrics are provided in the Supplementary Methods.

### Differential kinetic gene analysis

Based on STEER-inferred kinetic parameters, we performed differential analysis to identify differential kinetic genes (DKGs), defined as genes exhibiting significant differences in at least one kinetic parameter. DKGs were further classified according to the number and combination of differential kinetic parameters, allowing distinction among different patterns of transcriptional, splicing and degradation changes. Detailed definitions are provided in the Supplementary Methods.

## Supplementary Material

nwag199_Supplemental_Files

## Data Availability

All datasets used in this study are publicly available. The Annotated Erythroid Lineage of Mouse Gastrulation Data was obtained from the scVelo website (https://ndownloader.figshare.com/files/27686871) and originates from Pijuan-Sala *et al.*’s study [27]. The MURK gene list used in the Results section is accessible at https://github.com/mebarile/Gata1_Erythroid_kinetics. The Annotated Developing Mouse Hippocampus data was obtained from the scVelo command scvelo.datasets.dentategyrus_lamanno and originates from La Manno *et al.*’s study [9]. Human Hematopoiesis scNT-seq Data were retrieved using the Dynamo package via the command dyn.sample_data.hematopoiesis_raw. Preprocessed 10× Embryonic E18 Mouse Brain data were downloaded from https://github.com/welch-lab/MultiVelo, with the original data available through the 10× Genomics website at https://www.10xgenomics.com/resources/datasets/fresh-embryonic-e-18-mouse-brain-5-k-1-standard-1-0-0. The Bin-50 Stereo-seq Mouse Placentation Data (E8.5 S1) was acquired from the MPSTA database (https://db.cngb.org/stomics/mpsta/download/). The Cell-bin and Bin-60 Stereo-seq Mouse brain data were downloaded at Spateo [46] website (https://www.dropbox.com/s/seusnva0dgg5de5/mousebrain_cellbin_clustered.h5ad?dl=0) and (https://www.dropbox.com/s/wxgkim87uhpaz1c/mousebrain_bin60_clustered.h5ad?dl=0). The annotated OSCC data were downloaded at https://figshare.com/articles/dataset/Spatial_transcriptomics_reveals_distinct_and_conserved_tumor_core_and_edge_architectures_that_predict_survival_and_targeted_therapy_response_/20304456/1. The processed Smart-seq2 data was available with the GEO accession GSE204700. The raw Slide-seq data was download at https://singlecell.broadinstitute.org/single_cell/study/SCP815/sensitive-spatial-genome-wide-expression-profiling-at-cellular-resolution. The Slide-tags mouse brain data are from previous work by Gu *et al.* [29], and raw data can be accessed via GEO under accession code GSE244355. The python package of STEER is available at https://github.com/lzygenomics/STEER.
