# Supplementary material for: STEER: decoupling kinetics with Spatial-Temporal Explainable Expert model for RNA velocity inference
Source: Natl Sci Rev. 2026 Mar 30;13(9):nwag199. doi: 10.1093/nsr/nwag199 (PMC13220760; doi:10.1093/nsr/nwag199)
Supplement: nwag199_Supplemental_Files [file nwag199_supplemental_files.zip › Supplementary Figures.pdf]

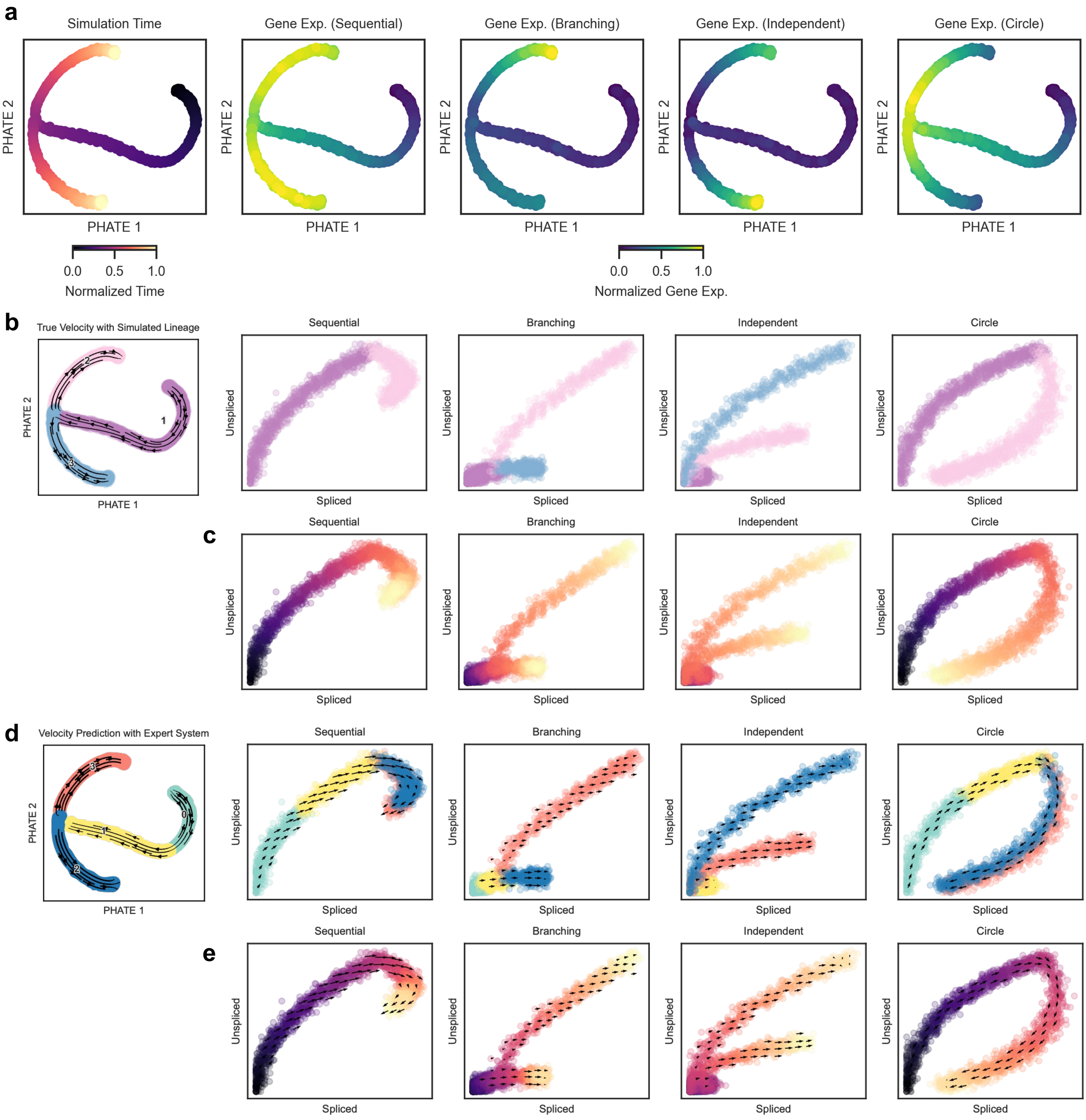

Supplementary Fig. 1. Simulation Design at the Cell and Gene Levels
(a) Cells are embedded in PHATE space based on simulated expression profiles of unspliced and spliced transcripts. The left panel is colored by simulated time (min-max normalized), while the right panels display the spliced expression levels of randomly selected genes following the Sequential, Branching, Independent, and Circle patterns. **(b)** Colors indicate the lineage design, comprising one progenitor lineage and two descending branching lineages. **(c)** For each gene pattern (Sequential, Branching, Independent, and Circle), cells are shown in both unspliced and spliced spaces, with colors representing the simulation time. **(d)** Arrows indicate the predicted velocity, and scatter points are colored by the predicted expert labels. **(e)** Colors represent the predicted time. In all panels, each point corresponds to a cell (n = 2,000 cells).

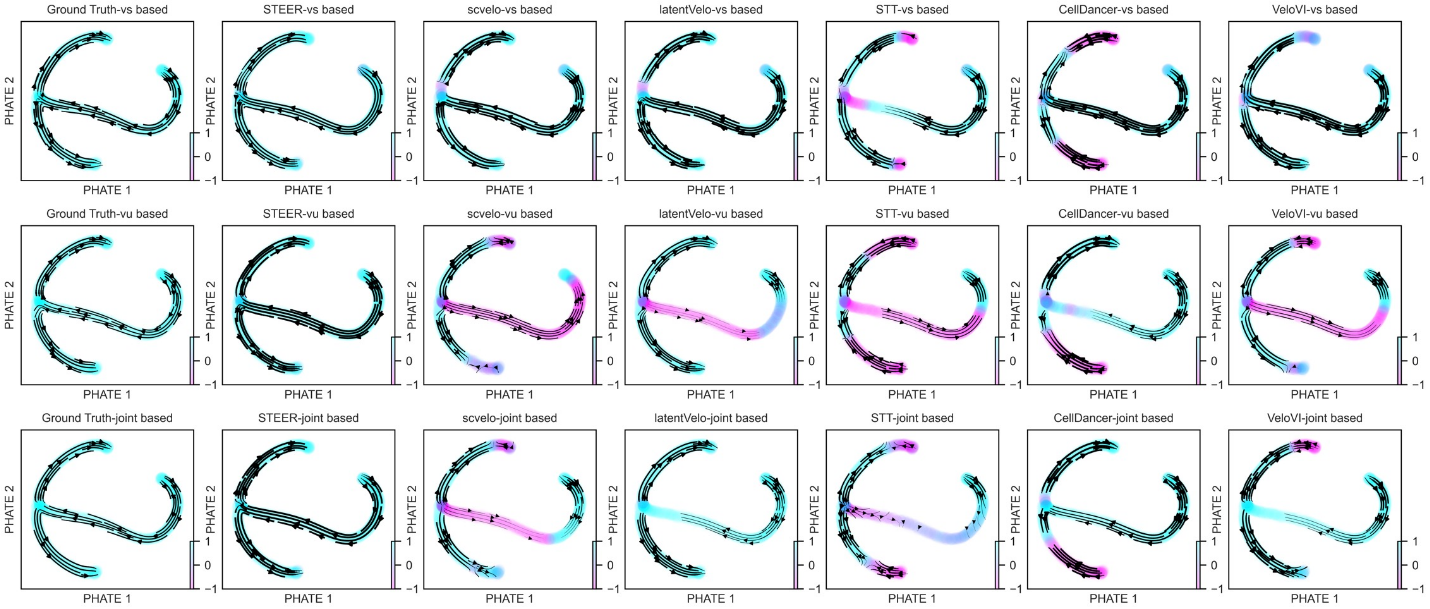

Supplementary Fig. 2. Cosine Similarity of Vector Fields Based on Predicted and Ground Truth Velocities
Cells are embedded in PHATE space using simulated expression profiles of unspliced and spliced transcripts, with color representing the cosine similarity between predicted and ground truth 2-dim velocity vectors. The top row displays velocity vectors derived from spliced velocity (denoted as vs), the middle row from unspliced velocity (denoted as vu), and the bottom row from the concatenated unspliced and spliced velocities (denoted as joint). The left column shows the ground truth, while the remaining columns compare the results from various methods. In these plots, each point corresponds to a cell (n = 2,000 cells).

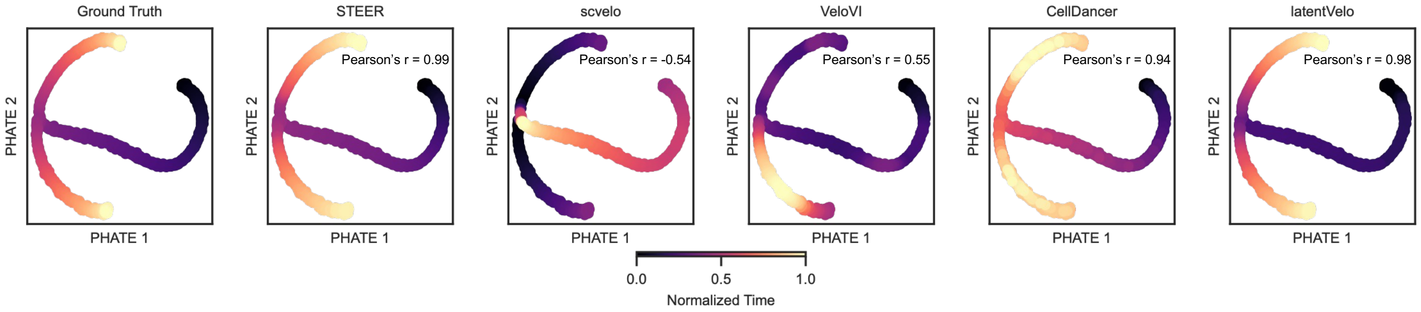

Supplementary Fig. 3. Comparison of Simulated and Predicted Time Across Different Methods
These panels display the predicted time under PHATE space for both the ground truth and all comparative methods, each point corresponds to a cell (n = 2,000 cells). The annotated values indicate the Pearson correlation coefficients between the predicted and simulated times.

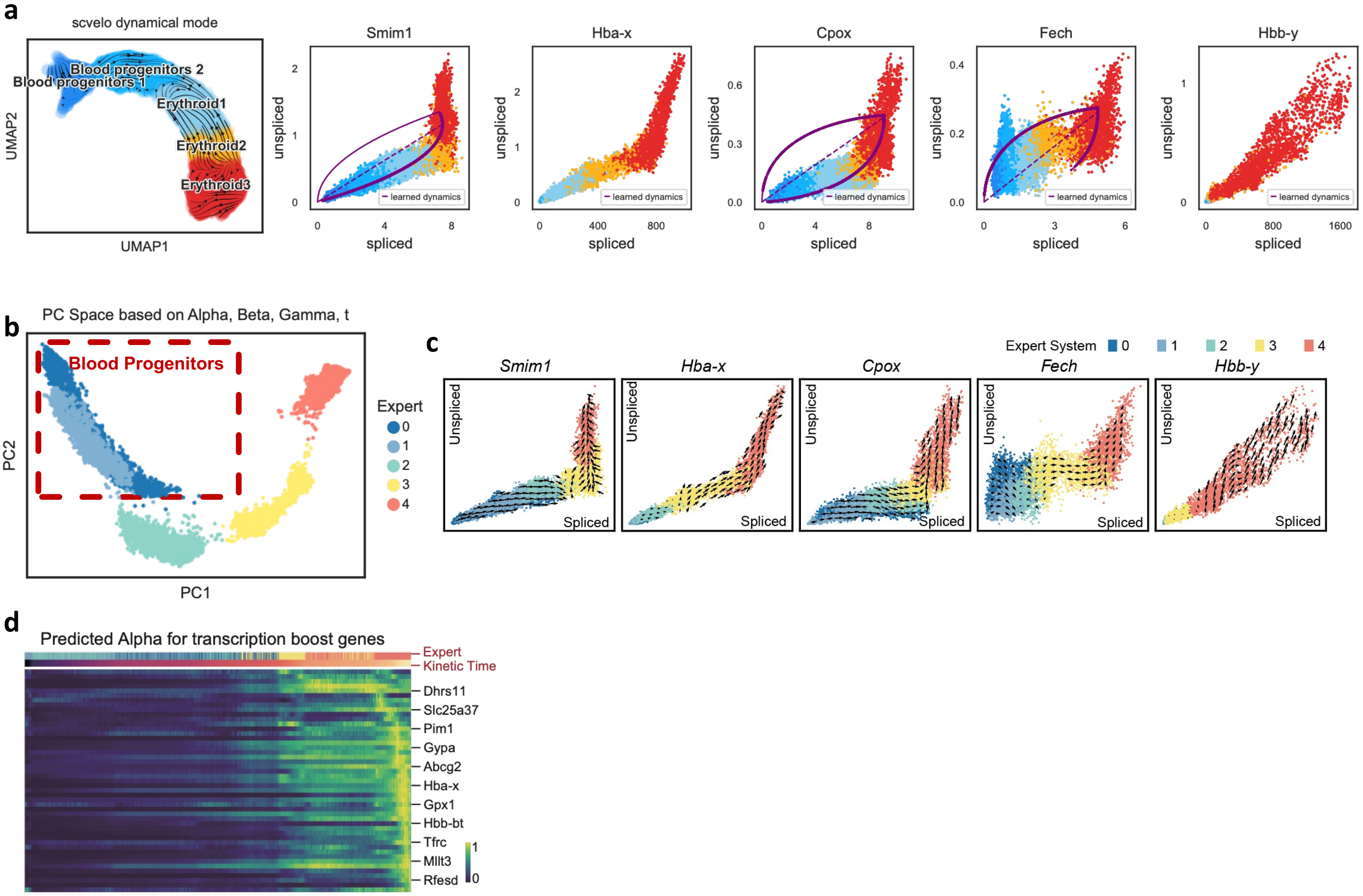

Supplementary Fig. 4. Evaluation using mouse erythroid development dataset
(a) Cell-level RNA velocity projections in UMAP space (left) and gene-specific velocity patterns in the transcriptional (u) and spliced (s) mRNA abundance space (right), computed by scVelo. Curves represent scVelo's fitted dynamics, while genes Hba-x and Hbb-y lack velocity curves due to their exclusion as velocity genes in scVelo, colored by cell type. Notably, scVelo shows an apparent backflow from erythroid cells toward blood progenitors in UMAP space, and for burst-like genes the fitted phase portraits often exhibit a repression-like lower branch that is inconsistent with the expected transcriptional upregulation during erythroid commitment. **(b)** Principal component (PC) space constructed from predicted kinetic parameters (α: transcription rate, β: splicing rate, γ: degradation rate) and kinetic time by STEER. These parameters collectively represent the full set of variables in the RNA velocity differential equations. **(c)** Cell-level RNA velocity projections in UMAP space (left) and gene-specific velocity patterns in the transcriptional (u) and spliced (s) mRNA abundance space (right), computed by STEER, colored by predicted Expert. **(d)** Heatmap of predicted α values by STEER for genes previously reported to exhibit transcription boost.

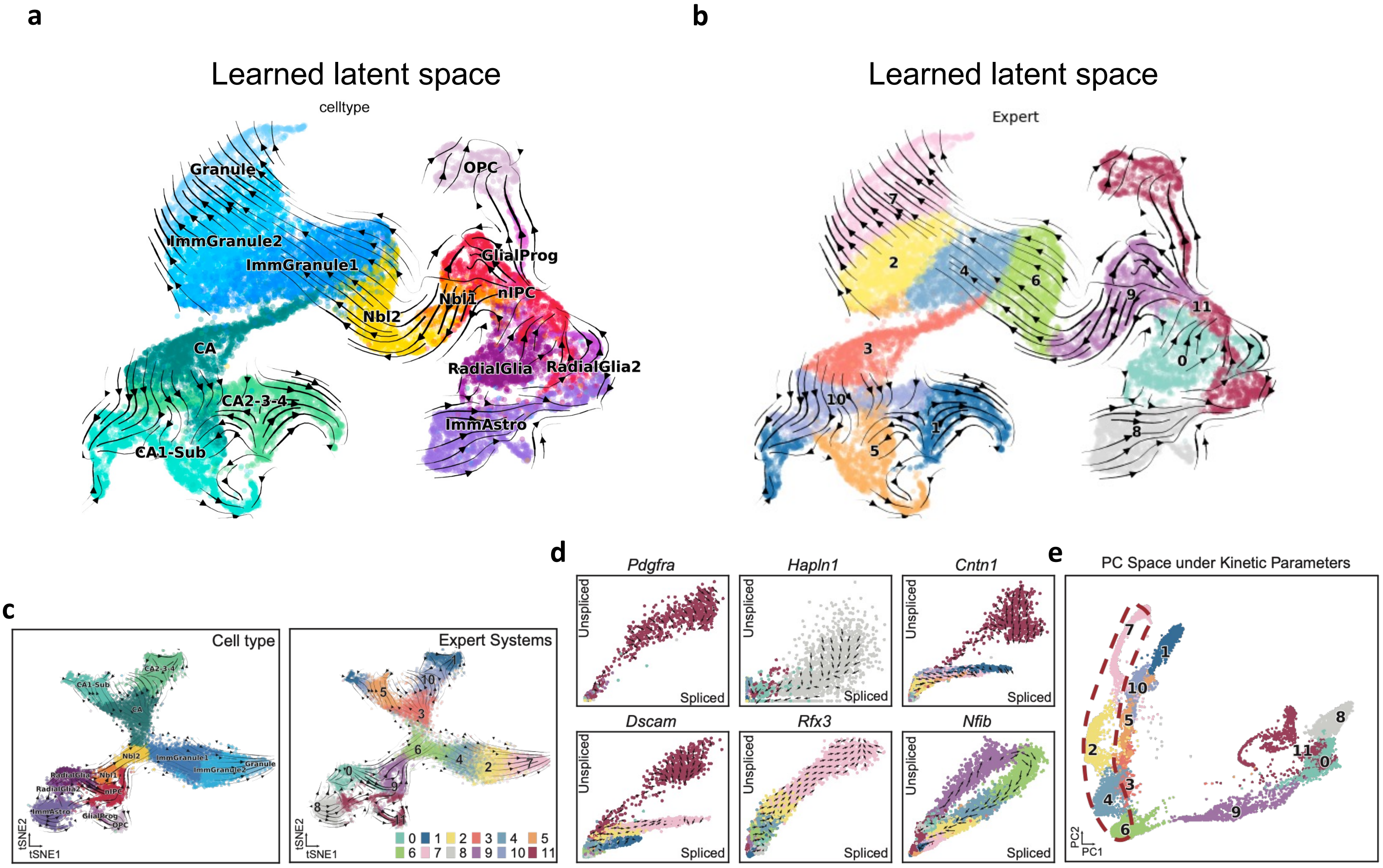

Supplementary Fig. 5. Evaluation using mouse hippocampus development dataset
(a, b) Cell-level RNA velocity UMAP projections under the learned latent space, colored by cell type and Expert Systems. **(c)** Cell-level RNA velocity projections in t-SNE space, colored by cell type and Expert Systems. **(d)** Gene-level RNA velocity projections in u-s space, colored by Expert Systems. **(e)** Principal component (PC) analysis based on predicted kinetic parameters—α (transcription rate), β (splicing rate), γ (degradation rate) and kinetic time by STEER. The red box highlights a local ordering relationship. These parameters collectively represent the complete set of variables in the RNA velocity differential equations.

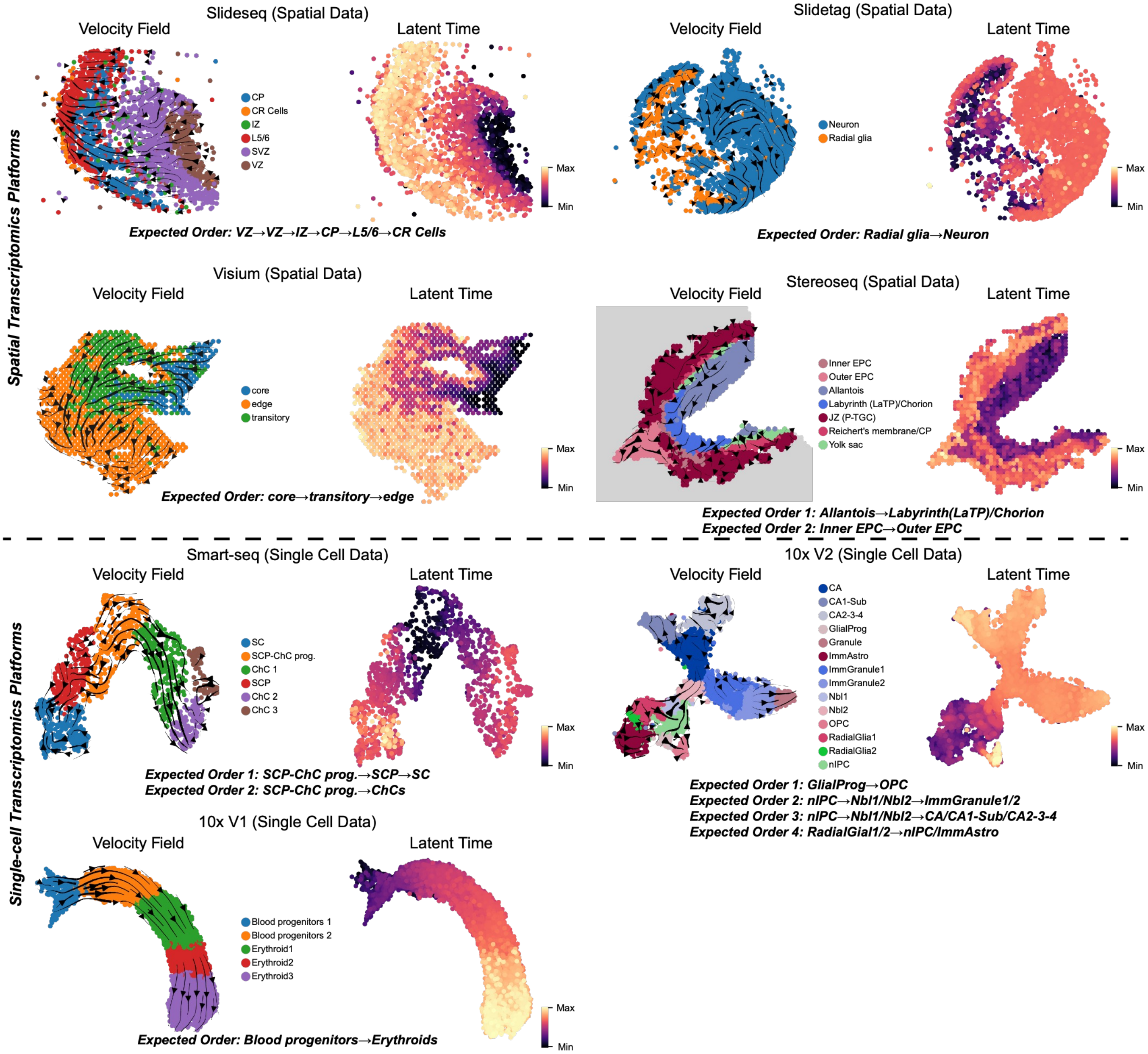

Supplementary Fig. 6. Robustness of STEER across diverse transcriptomic platforms.
STEER was benchmarked on seven datasets spanning major spatial (top rows) and single-cell (bottom rows) modalities. Left columns: RNA velocity fields colored by cell type or spatial domain. Right columns: Inferred cell-level latent time. The evaluation covers high-resolution/sparse spatial methods (Slide-seq, Slide-tag), heterogeneous cancer tissue (Visium OSCC), large-scale spatial profiles (Stereo-seq), and single-cell platforms with varying sequencing depths (Smart-seq2, 10x Genomics). Text indicate biologically expected trajectories.

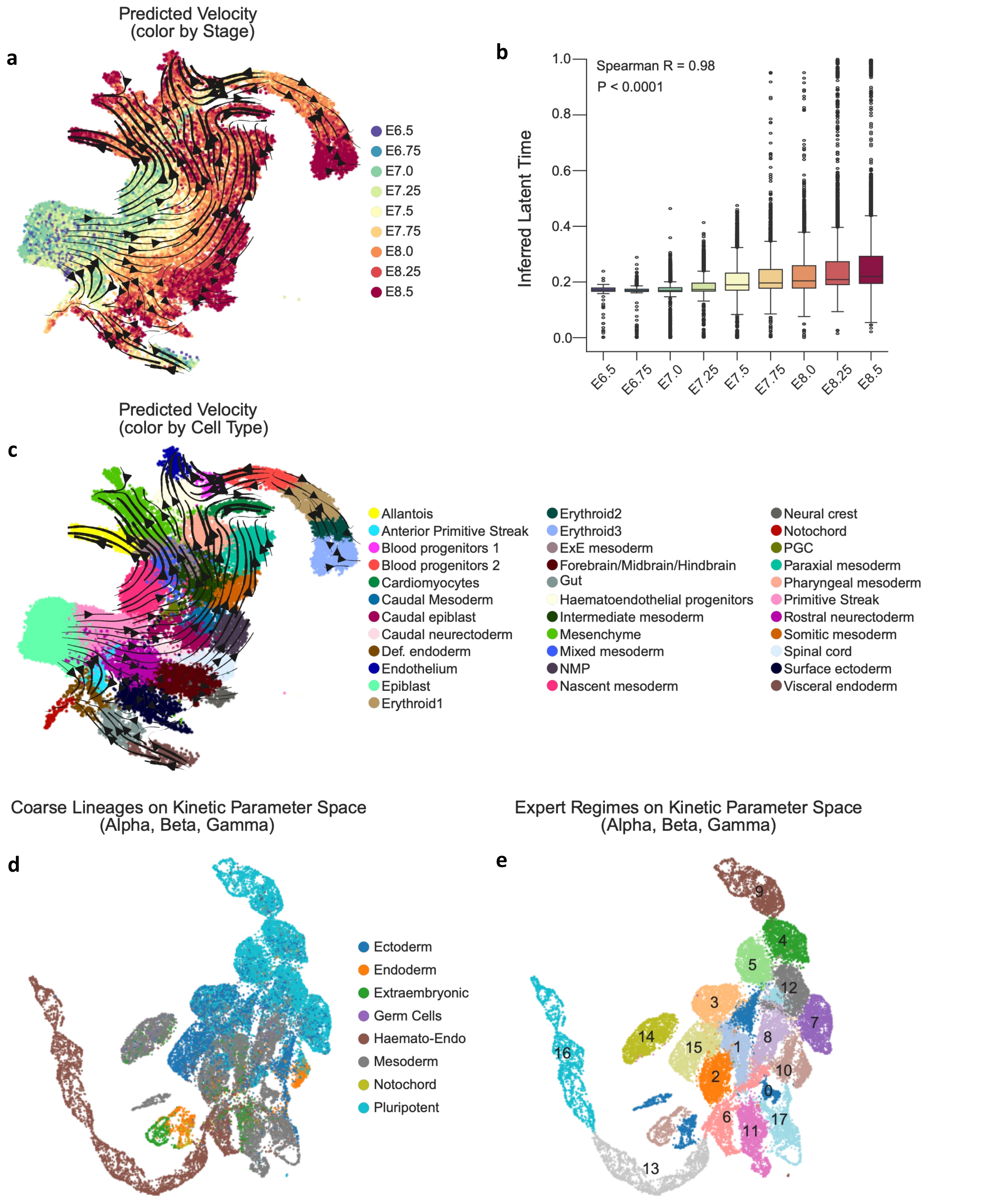

Supplementary Fig. 7. STEER resolves complex whole-embryo dynamics and disentangles multi-lineage kinetics in mouse gastrulation.

(a) RNA velocity streamplots of mouse gastrulation (E6.5–E8.5) colored by developmental stage **(a)** and cell type **(c)**, demonstrating accurate reconstruction of global temporal progression and distinct lineage trajectories. **(b)** STEER-inferred latent time shows strong quantitative alignment with physical developmental stages (Spearman $R=0.98$, $P<0.0001$; calculated on stage means). **(d, e)** Projection of cells into the learned kinetic parameter space (α,β,γ embedding) reveals clear segregation of major lineages **(d)** and Expert regimes **(e)**, confirming the model's capacity to disentangle heterogeneous kinetics across complex multi-lineage systems. For computational efficiency, the training is on a random subset of 30,000 cells.

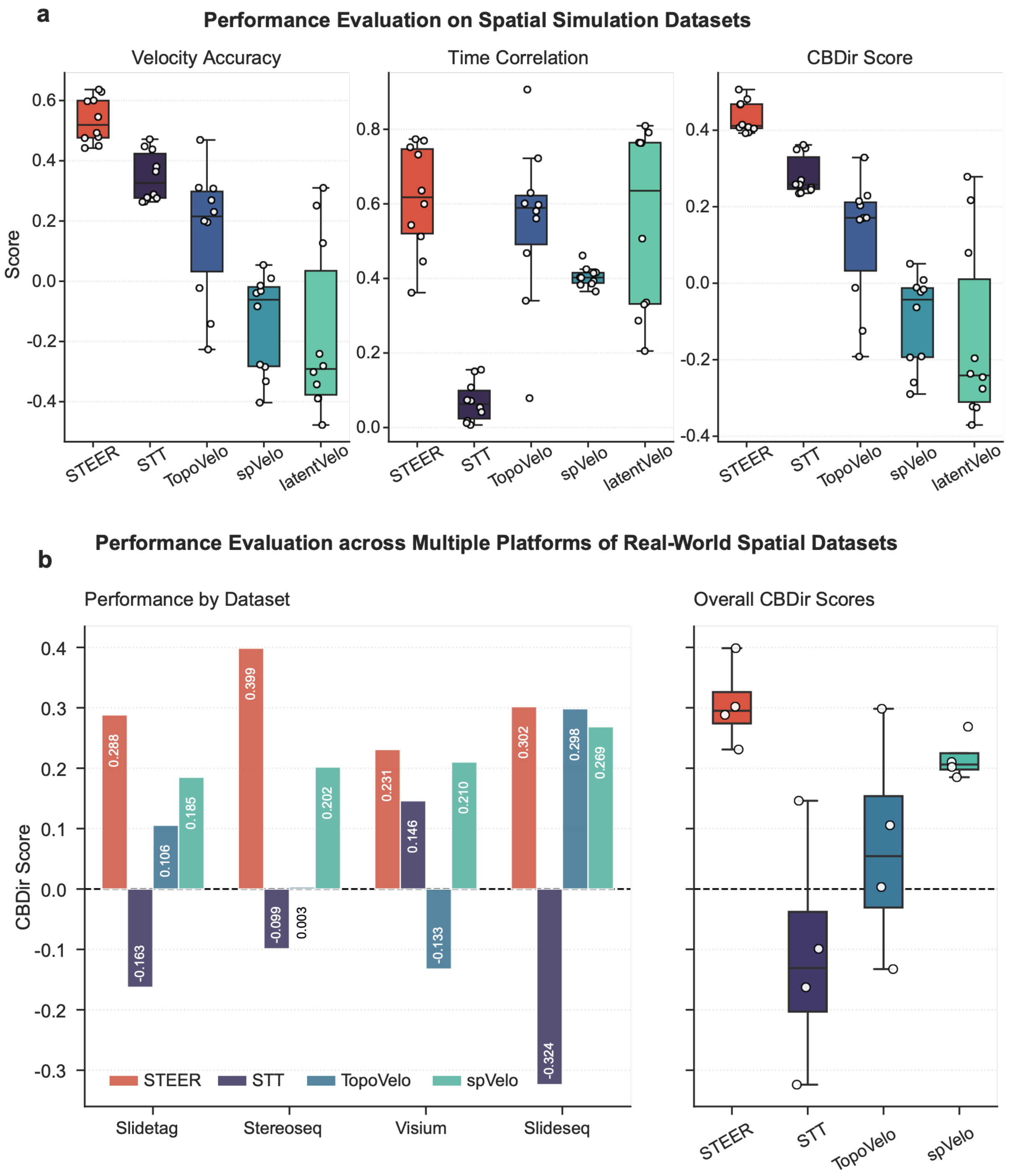

Supplementary Fig. 8. Quantitative evaluation on spatial simulation and real-world spatial transcriptomics datasets.

(a) Performance comparison on spatial simulation datasets. Boxplots summarize velocity accuracy, latent-time correlation, and CBDir scores across methods. Each point represents one of 10 simulation replicates spanning bilinear and multi-radial dynamical scenarios.

(b) Performance on real-world spatial transcriptomics datasets across multiple platforms (Slide-tag, Stereo-seq, Visium, and Slide-seq). Left, dataset-wise CBDir scores. Right, distributions of CBDir scores aggregated across datasets. Higher scores indicate better agreement between inferred dynamics and prior biological ordering.

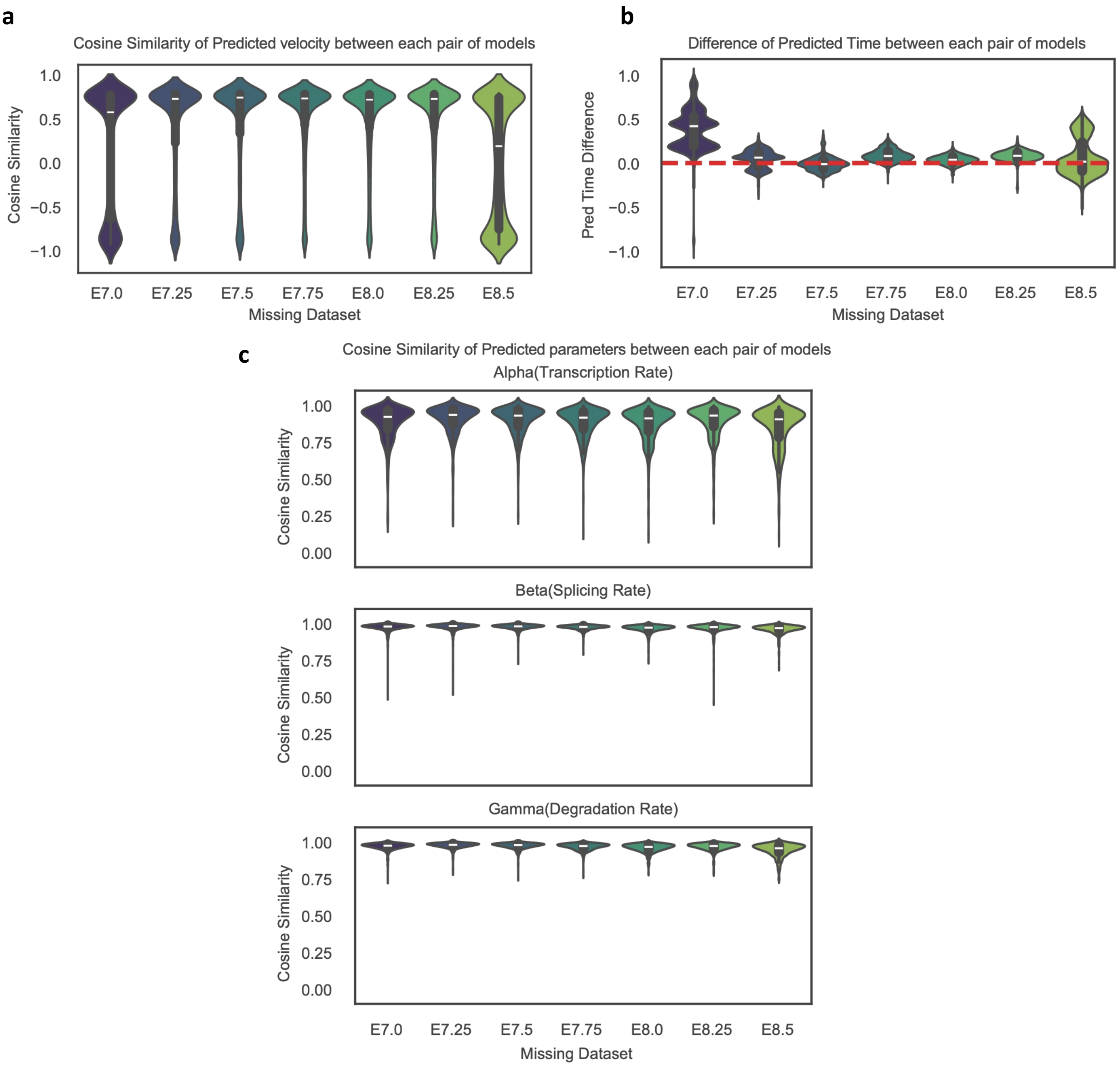

Supplementary Fig. 9. Generalization test using single-stage dropout
(a) Violin plots depicting cosine similarities of predicted velocities for each cell between models trained on the complete dataset and those trained with a single stage excluded. Each violin represents a different excluded stage. **(b)** Violin plots illustrating differences in predicted times between pairs of models. **(c)** Violin plots showing cosine similarities of predicted parameters between model pairs: alpha (top panel), beta (middle panel), and gamma (bottom panel).

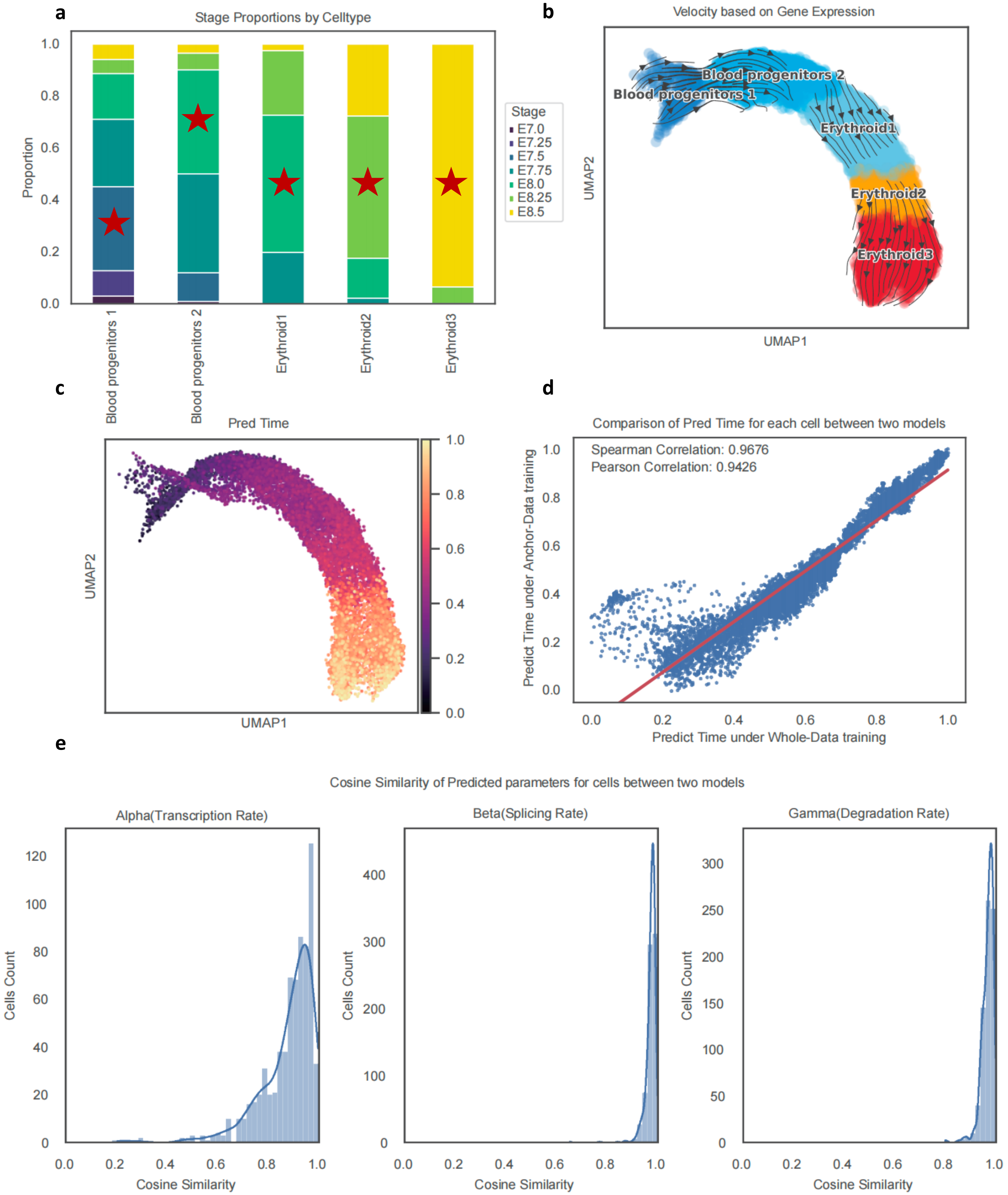

Supplementary Fig. 10. Generalization test using multiple-stage dropout
(a) Stacked bar plot illustrating the stage proportions for each cell type. Red stars denote the most enriched stage for each cell type, retained as an anchor to preserve primary cell type characteristics. **(b)** Velocity plot displaying predicted velocities mapped onto gene-expression UMAP space, colored by cell type. **(c)** UMAP plot colored by predicted time values. **(d)** Scatter plot comparing time predictions for each cell between the model trained on the full dataset and the model trained using only the anchor dataset. **(e)** Histograms depicting cosine similarities between the two models for predicted alpha (first panel), beta (second panel), and gamma (third panel) parameters for each cell. Counts represent the number of cells within each cosine similarity bin.

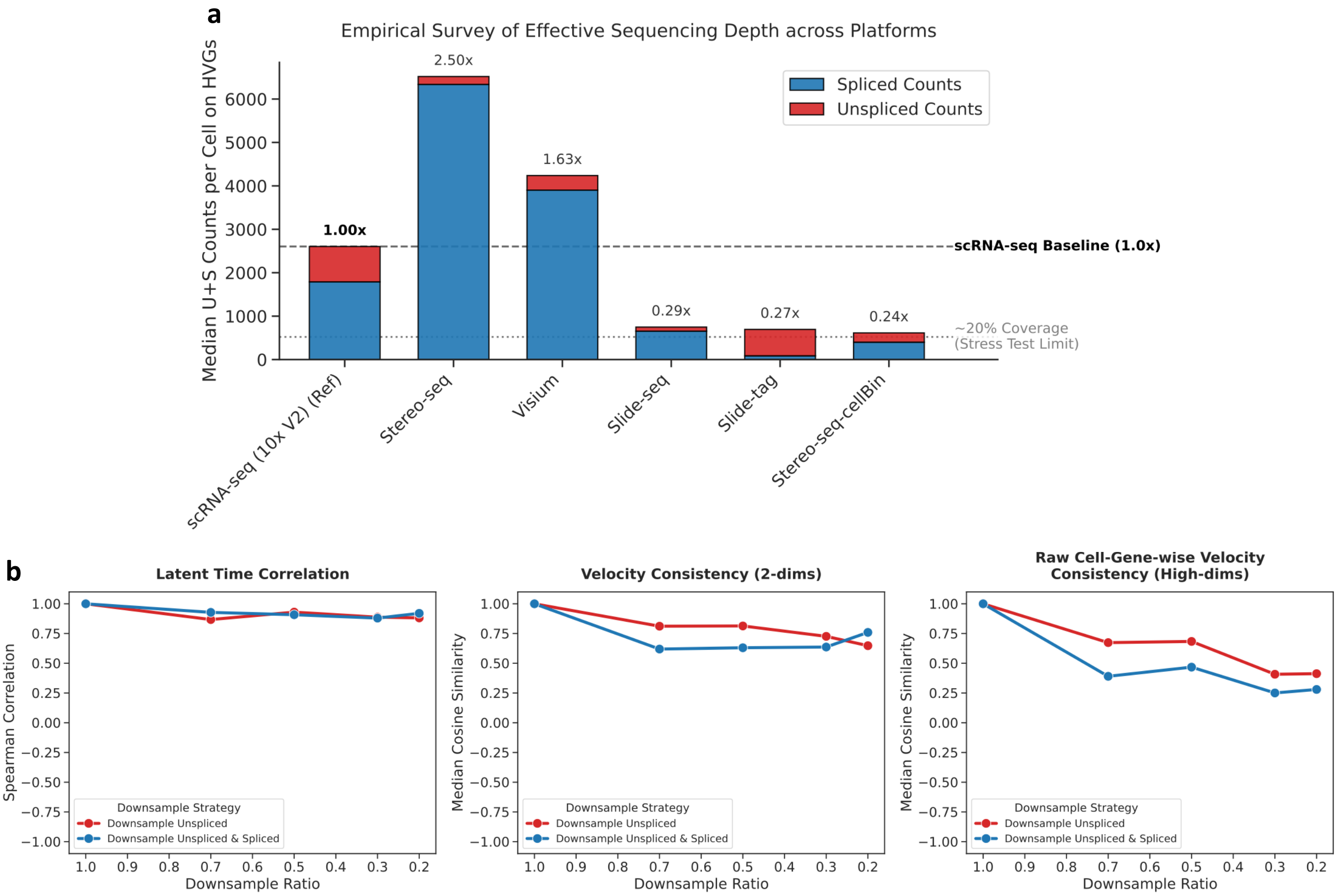

Supplementary Fig. 11. Robustness evaluation of STEER across downsampling strategy.

(a) Comparison of effective kinetic coverage across transcriptomic platforms. Bar heights represent the median combined unspliced (Red) and spliced (Blue) counts per cell calculated on highly variable genes. The black dashed line indicates the baseline depth of the reference 10x Genomics v2 dataset. The survey reveals that single-cell resolution spatial platforms (e.g., Slide-seq, Stereo-seq cell-bin) typically retain only ~25–30% of the effective sequencing depth found in standard scRNA-seq, with unspliced counts being particularly sparse. The gray dotted line denotes the 20% threshold used for robustness benchmarking.

(b) Performance benchmarks on the mouse Dentate Gyrus dataset downsampled from ratio 1.0 to 0.2 using two strategies: reducing only unspliced counts (Red) or both unspliced and spliced counts (Blue). (left) Latent Time Correlation: Spearman correlation of inferred latent time relative to the full-depth data. (middle) Visual Consistency (TSNE): Median cosine similarity of velocity vectors projected into the TSNE embedding space. (right) Raw Cell-Gene-wise Velocity Consistency: Median cosine similarity of high-dimensional velocity vectors computed on aligned genes.

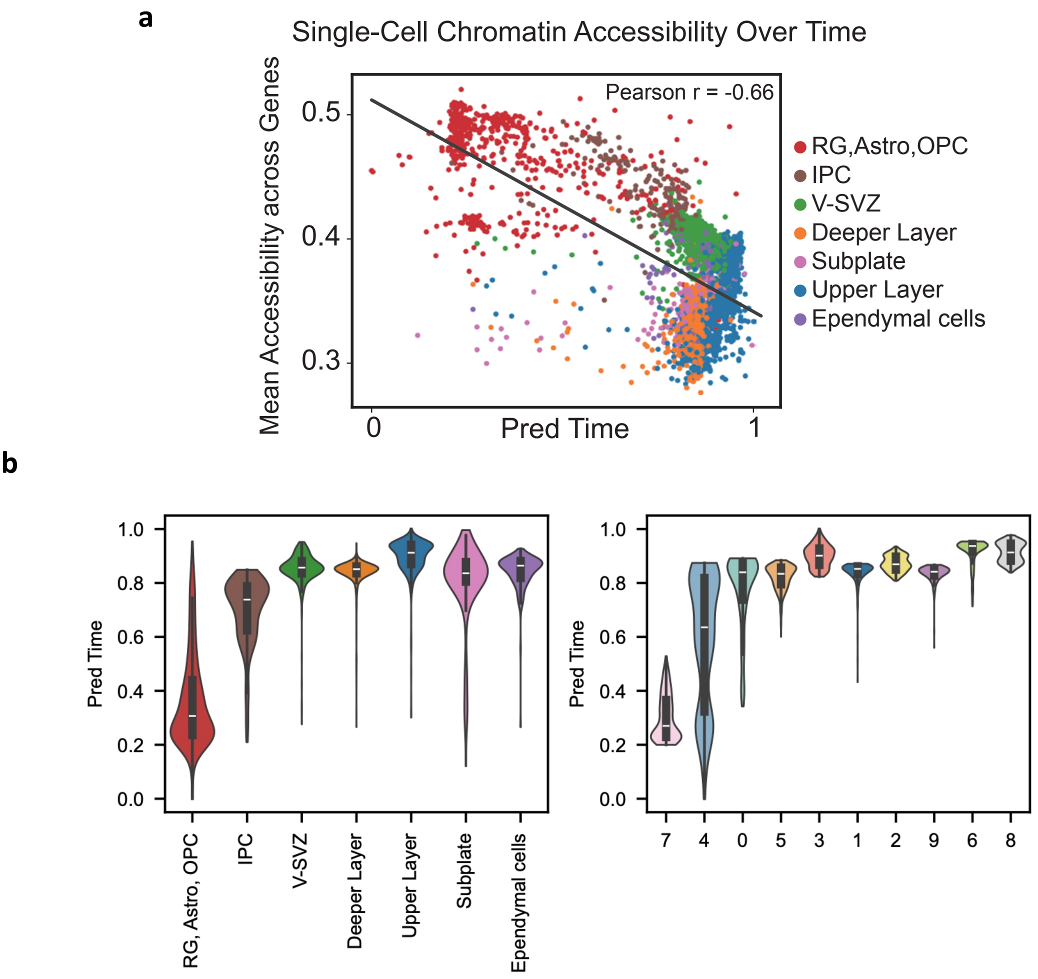

Supplementary Fig. 12. Evaluation of predicted time on Multi-omics Mouse Brain Data (E18)

(a) Chromatin accessibility (averaged across genes) per cell, ordered by STEER-inferred time and colored by cell type. Black line indicates linear regression.

(b) Predicted time points, summarized by cell type and Expert System (color-coded).

Note: A total of 4,881 cells are represented in both panels (a) and (b).

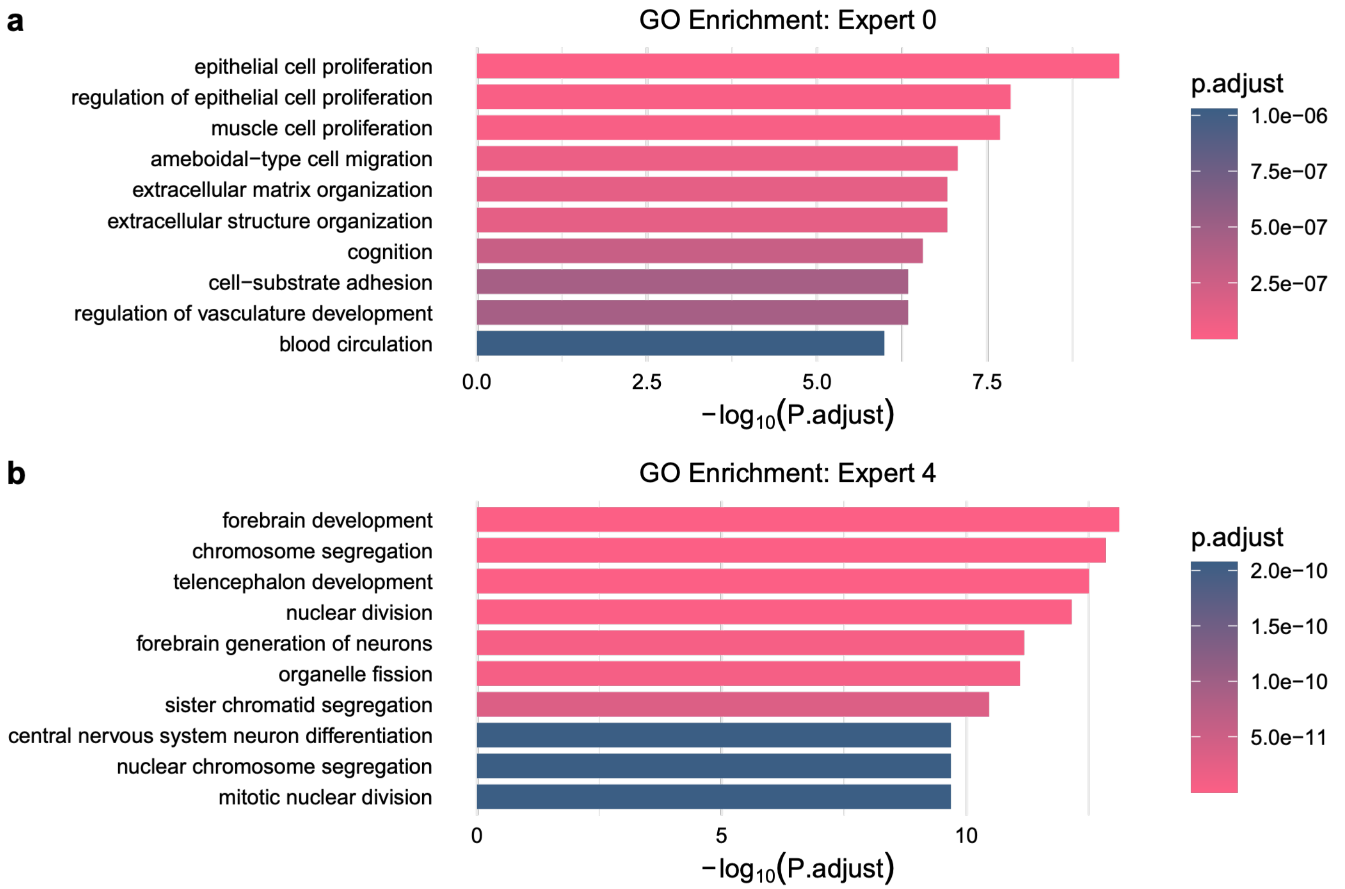

Supplementary Fig. 13. Functional characterization of differential kinetic programs between Expert 0 and Expert 4 within the annotated RG, Astro, OPC population.

(a, b) Bar plots displaying the top enriched Gene Ontology (GO) Biological Process terms for genes identified with differential kinetic rates (predicted alpha) specific to Expert 0 and Expert 4. The analysis was performed on the "RG, Astro, OPC" population to resolve the functional divergence between these two neighboring Experts. (a) Expert 0 is enriched for terms related to tissue structure and remodeling, such as "extracellular matrix organization" and "cell-substrate adhesion," consistent with the supportive role of the ependymal lineage. (b) Expert 4 is enriched for neurogenic and proliferative terms, such as "forebrain development" and "nuclear division," consistent with neuronal lineage commitment. The x-axis represents the significance of enrichment (−log10 adjusted P-value), and bars are colored according to the adjusted P-value (Benjamini-Hochberg correction).

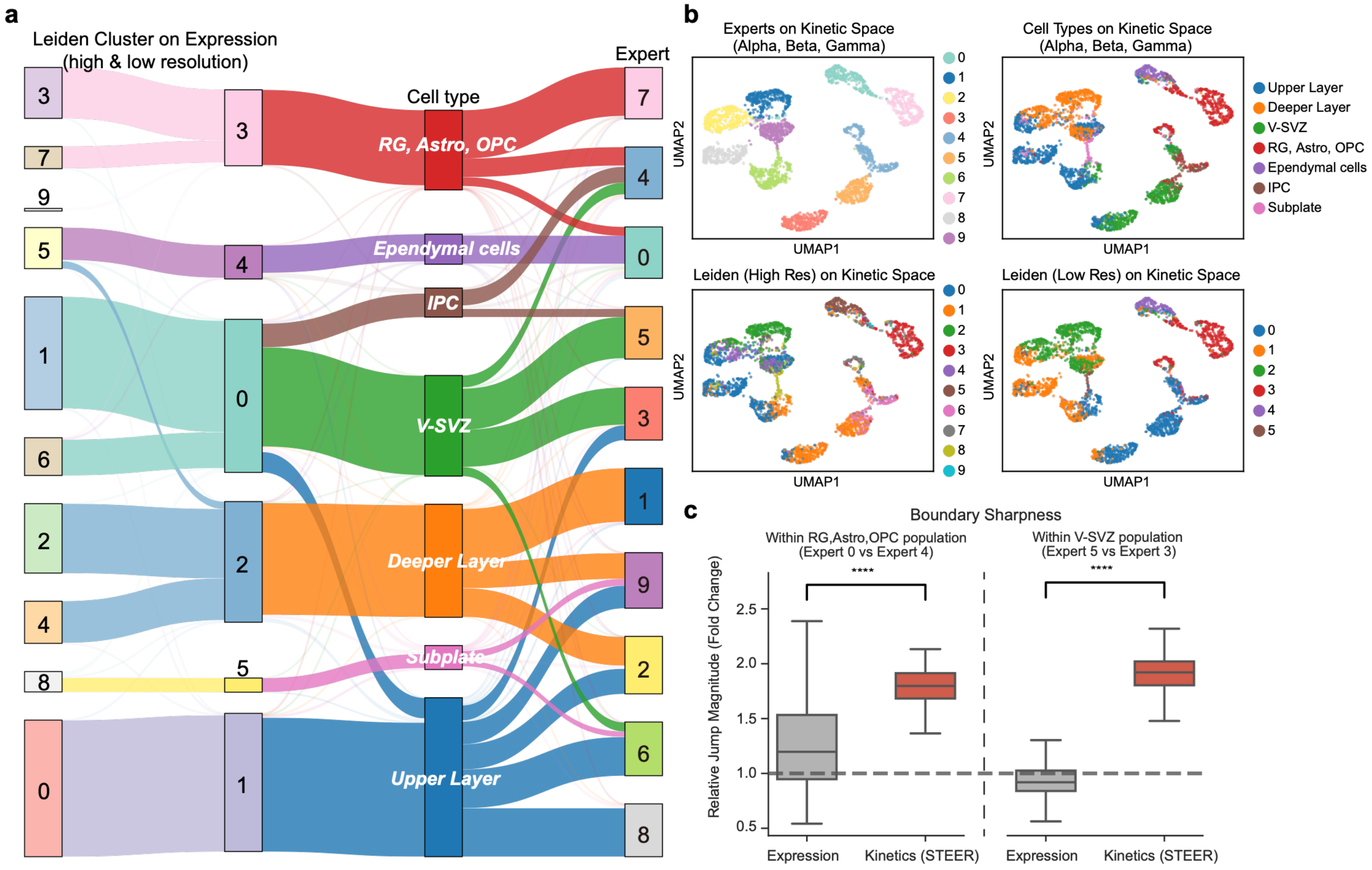

Supplementary Fig. 14. STEER experts capture distinct kinetic regimes beyond transcriptomic clustering.
(a) Sankey diagram illustrating the correspondence between expression-derived Leiden clusters (left), annotated cell types (middle), and STEER Expert systems (right). The flows visualize how cells from the "RG, Astro, OPC" and "V-SVZ" populations are distributed across different clustering resolutions and Expert assignments.
(b) UMAP projections of cells based on the inferred kinetic parameter space (α,β,γ). The four panels display the same kinetic embedding colored by: STEER Expert systems (top left), ground-truth cell type annotations (top right), high-resolution Leiden clusters (bottom left), and low-resolution Leiden clusters (bottom right).
(c) Quantification of boundary sharpness at the interfaces between neighboring Experts. Box plots show the relative jump magnitude (defined as the fold-change of boundary-pair distance relative to the mean internal-pair distance) calculated in transcriptomic space (“Expression”) versus kinetic parameter space (“Kinetics”). Comparisons are shown for the interface between Expert 0 and Expert 4 within the “RG, Astro, OPC” population (left) and between Expert 5 and Expert 3 within the "V-SVZ" population (right). The horizontal dashed line (y=1) indicates the baseline distance observed between neighbors within the same Expert. Statistical significance was assessed using a two-sided Mann–Whitney U test (****$P<0.0001$). Center lines indicate medians; box limits indicate the 25th and 75th percentiles; whiskers extend to 1.5 times the interquartile range.

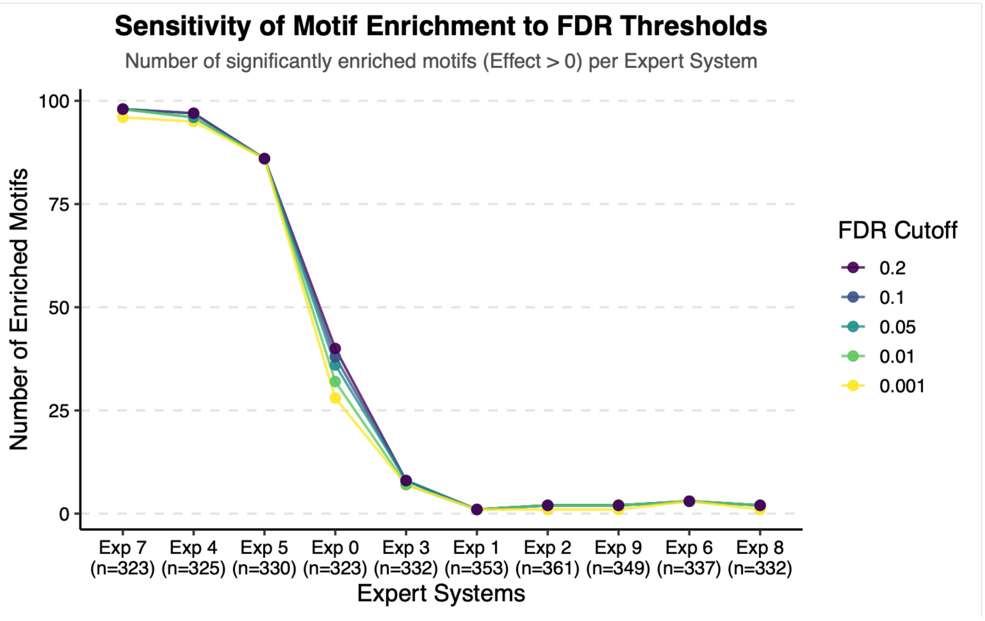

Supplementary Fig. 15. Sensitivity analysis of expert-associated motif enrichment across statistical thresholds.

The line plot shows the number of significantly enriched transcription factor motifs (ChromVAR effect size > 0) identified for each Expert System under varying False Discovery Rate (FDR) thresholds (ranging from 0.001 to 0.2). Significance was assessed using a one-vs-all Wilcoxon rank-sum test. The x-axis indicates the Expert ID and the corresponding number of cells (n).

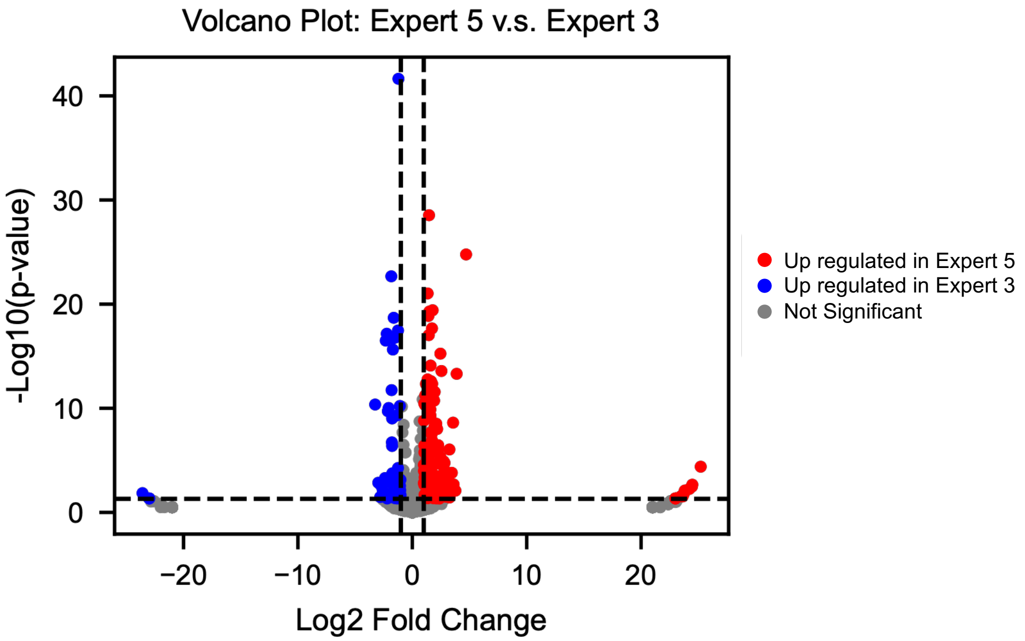

Supplementary Fig. 16. Volcano Plot of Differentially Expressed Genes Between Expert 5 and Expert 3
Differential gene expression was assessed using a *t*-test. Genes with a significance cutoff of $p \leq 0.05$ and $\log_2$ fold change (log2FC) ≥ 1 were considered upregulated in Expert 5 (colored in red). Conversely, genes with log2FC ≤ -1 and $p \leq 0.05$ were considered upregulated in Expert 3 (downregulated in Expert 5) and are colored in blue.

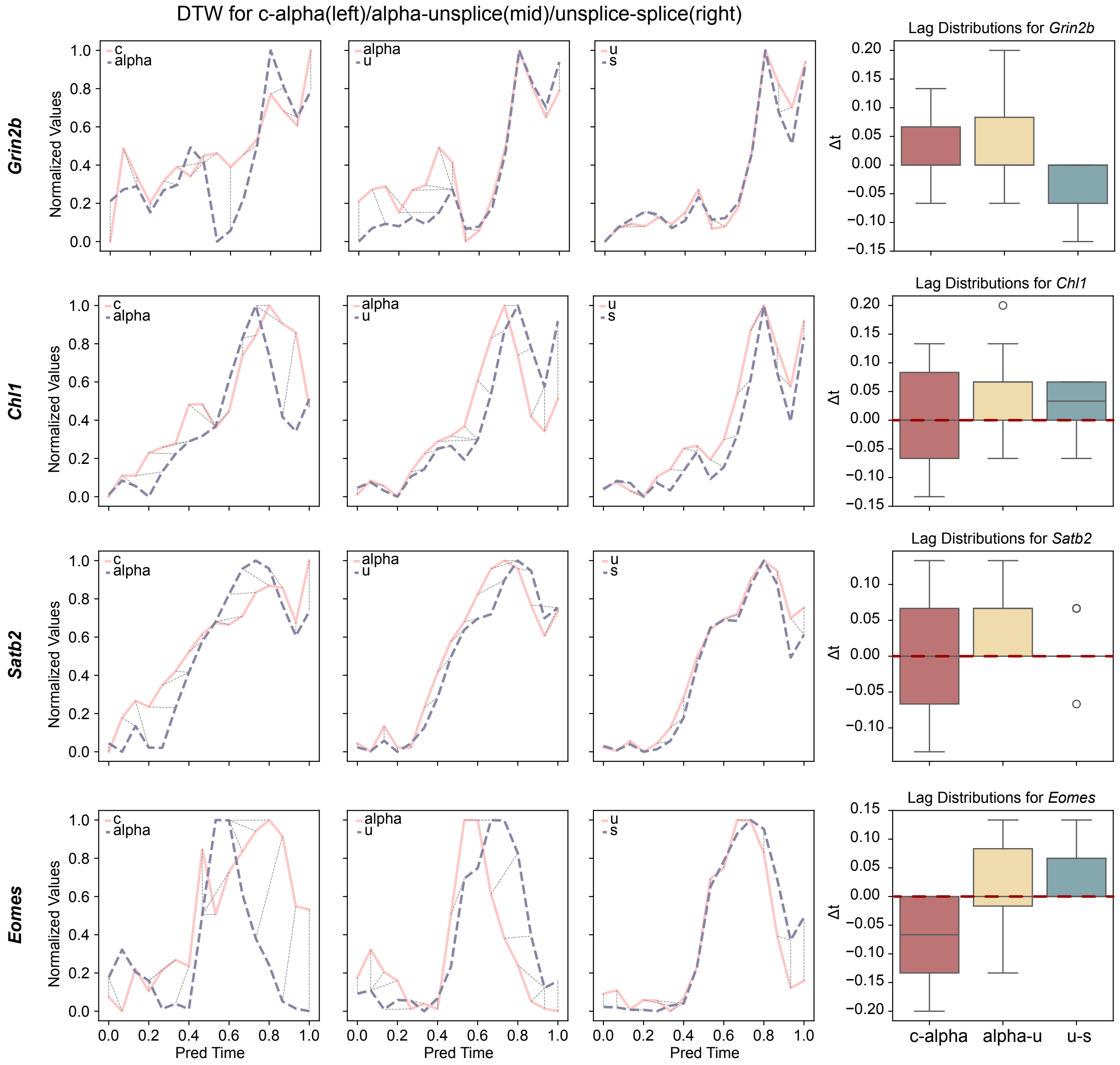

Supplementary Fig. 17. Gene-level illustration of dynamic time warping (DTW) alignment in multi-omics mouse brain data.

Line plots illustrate dynamic time warping (DTW) alignment of gene-level molecular trajectories along the predicted kinetic time. The y-axis shows normalized values of chromatin accessibility, predicted transcription rate (α), unspliced RNA, and spliced RNA. Columns are organized by regulatory pairs: the first column compares chromatin accessibility and α; the second compares α and unspliced RNA; and the third compares unspliced and spliced RNA. Solid pink lines denote the modality theoretically expected to occur earlier based on prior knowledge, whereas dashed dark-blue lines denote the modality expected to occur later. Fine dotted gray lines indicate aligned time points after DTW. Trajectories were coarse-grained into 15 equally sized bins, with values averaged within each bin and connected by line segments. The final column summarizes the distribution of time lags (Δt) between each regulatory pair shown in the first three columns, computed as differences between aligned time points after DTW. Each box in the boxplot represents the distribution of lag values across the 15 bins.

Note: Genes shown here were selected based on established roles in cortical neurodevelopment and representative kinetic patterns and are intended as illustrative examples of inferred cis-regulatory relationships. For quantitative inference of chromatin–transcription ordering, see the global, stage-resolved analysis in Supplementary Fig. 18. Analyses were restricted to expert-defined cell populations informed by prior biological knowledge: Eomes (an IPC marker) was evaluated in expert 4 (IPC-associated); Satb2 in upper-layer-associated experts 3, 6, and 8; and Grin2b and Chl1, which lack clear regional specificity, across neuronal-lineage-related experts excluding expert 0 (ependymal-associated).

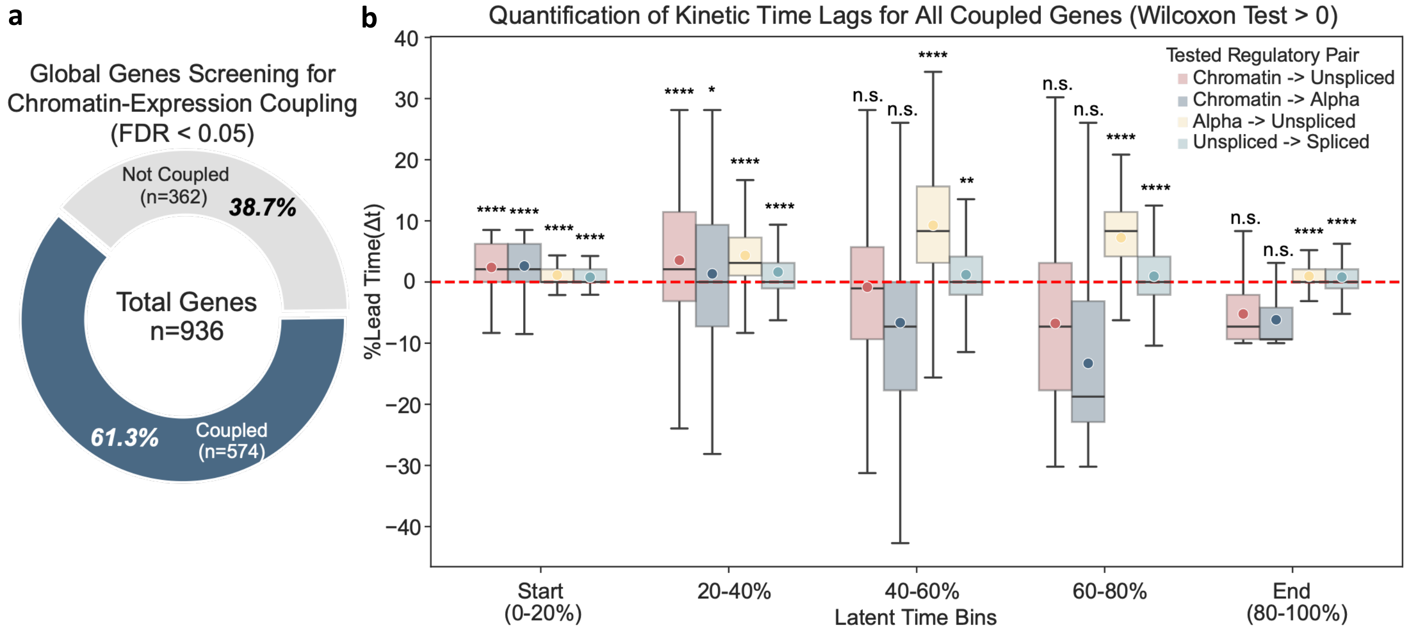

Supplementary Fig. 18. Global quantitative analysis of kinetic time lags reveals stage-dependent chromatin priming.

(a) For each gene, Spearman's rank correlation between chromatin accessibility and unspliced RNA counts along latent time was computed. Statistical significance was assessed using permutation tests (n = 1,000 permutations of time bins) followed by Benjamini–Hochberg false discovery rate (FDR) correction. Genes with FDR < 0.05 were classified as significantly coupled and retained for downstream analysis. The donut chart summarizes the proportions of coupled and uncoupled genes. **(b)** For significantly coupled genes, time lags (Δt) were estimated using dynamic time warping (DTW) for four regulatory pairs: ATAC → Unspliced, ATAC → α, α → Unspliced, and Unspliced → Spliced. Lag distributions were computed within five consecutive latent-time bins. Boxplots show the median and interquartile range, with points indicating the mean lag. Statistical significance of positive lag values (Δt > 0) was assessed using one-sided Wilcoxon signed-rank tests (*P < 0.05, **P < 0.01, ***P < 0.001, ****P < 0.0001; n.s., not significant).

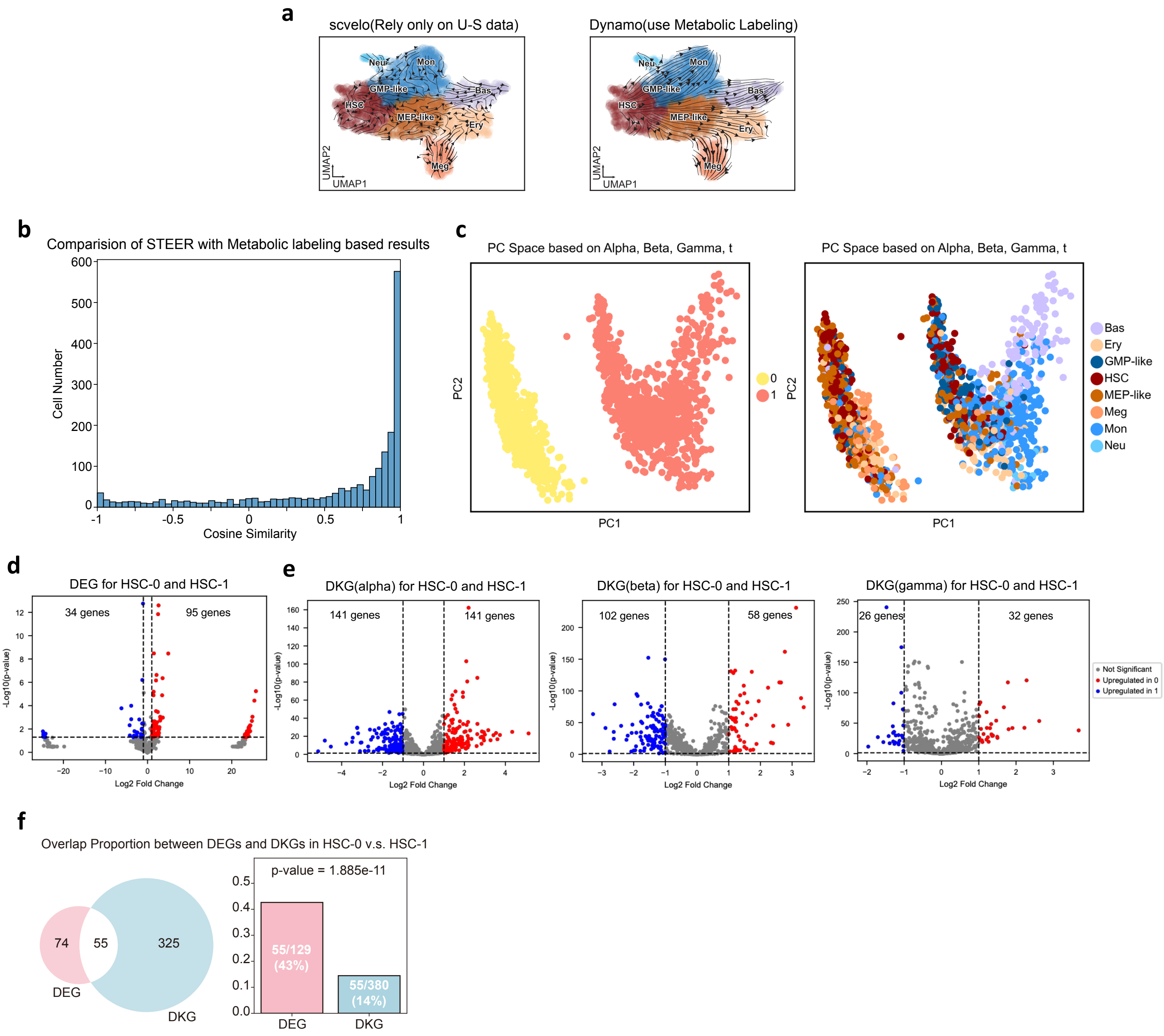

Supplementary Fig. 19. STEER captures early-stage kinetics driving lineage specification in human hematopoietic dataset

(a) RNA velocity projections in UMAP space from scVelo (left), and Dynamo (right). scVelo rely on unspliced and spliced data; Dynamo additionally incorporates metabolic labeling.

(b) Histogram showing the distribution of cosine similarities between RNA velocity vectors inferred by STEER (based solely on unspliced and spliced RNA) and by Dynamo (which incorporates metabolic labeling).

(c) Principal component analysis of STEER-inferred kinetic parameters—α (transcription rate), β (splicing rate), γ (degradation rate), and kinetic time—for human hematopoietic dataset, colored by Expert Systems (left) and by cell type (right).

(d, e) Differential gene expression (**d**) and differential kinetic parameters (**e**, α, β, γ) between the two expert systems in HSC were assessed using a *t*-test. Genes with $p \leq 0.05$ and $\log_2$ fold change (log2FC) ≥ 1 were considered upregulated in Expert 0 (red); genes with log2FC ≤ –1 and $p \leq 0.05$ were considered upregulated in Expert 1 (blue). The number of significant genes is indicated.

(f) Venn diagram showing the overlap between differentially expressed genes (DEGs) and differentially kinetic genes (DKGs) in the two HSC expert systems. The accompanying bar plot displays the proportion of genes overlapping exclusively with DEGs or DKGs. Statistical significance was assessed using a two-proportion *z*-test (two-tailed).

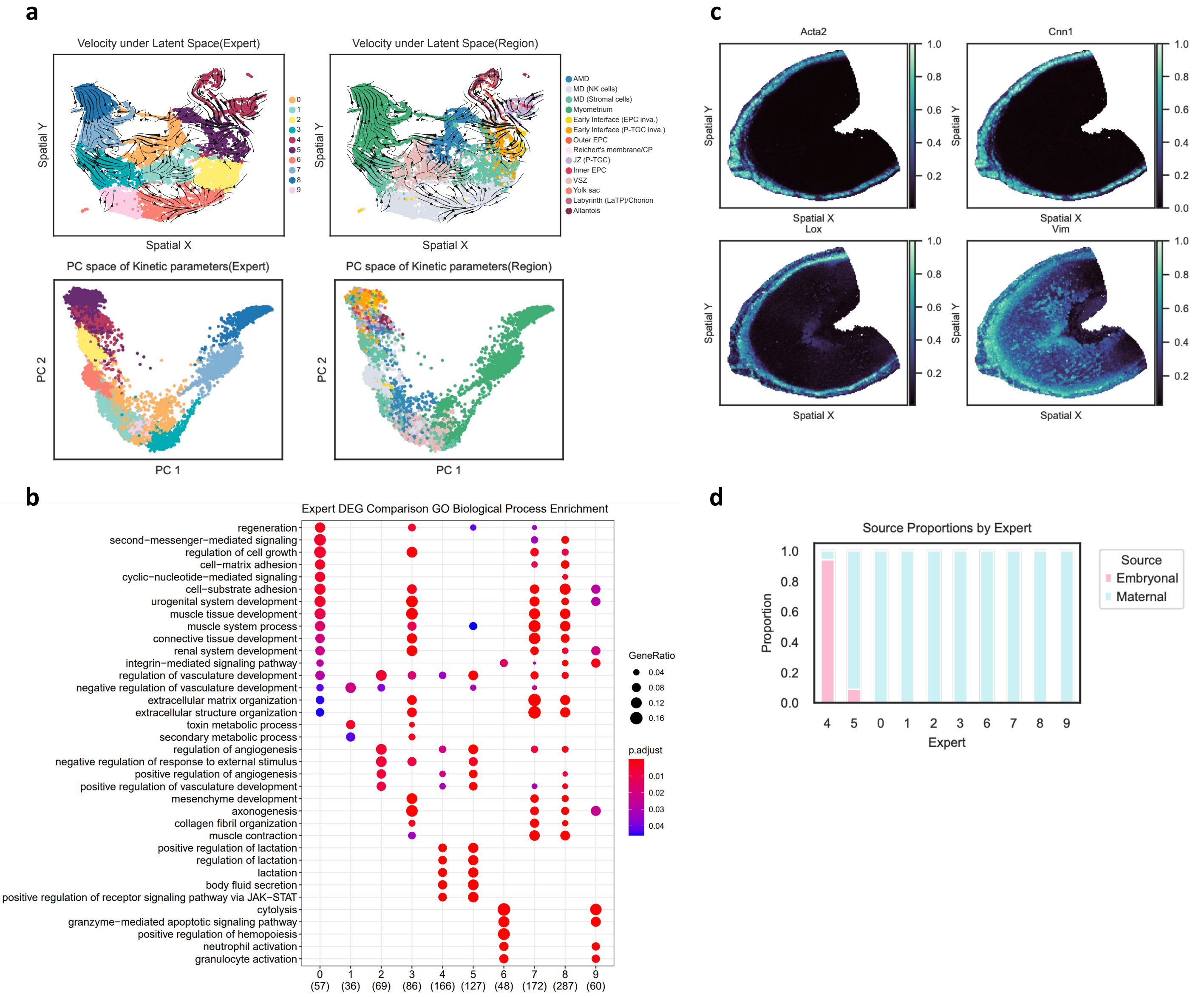

Supplementary Fig. 20. Expert systems decompose mixed dynamics in mouse placentation spatial transcriptomic data

(a) Top: Predicted RNA velocity in the learned latent space. Color encodes expert system (left) and spatial region (right). Bottom: Principal component analysis (PCA) of STEER-inferred kinetic parameters—transcription rate (α), splicing rate (β), degradation rate (γ), and kinetic time—colored by expert system (left) and by spatial region (right).
(b) Gene Ontology (GO) pathway enrichment of differentially expressed genes (DEGs) for each expert. DEGs were identified using a two-sided *t*-test with adjusted *p*-values ≤ 0.05 and $\log_2$ fold change ≥ 1. The number of upregulated genes per expert is indicated below the x-axis. Dot color encodes adjusted *p*-values, and dot size represents gene ratio (enriched gene count relative to pathway size). *P*-values were corrected using the Benjamini–Hochberg method.
(c) Spatial expression of four genes associated with the myometrium–decidua interface, colored by normalized expression values.
(d) Stacked bar plot showing tissue source composition (embryonic vs. maternal) for each expert system.
Note: Panels (a), (c) include 13,258 spatial transcriptomic spots.

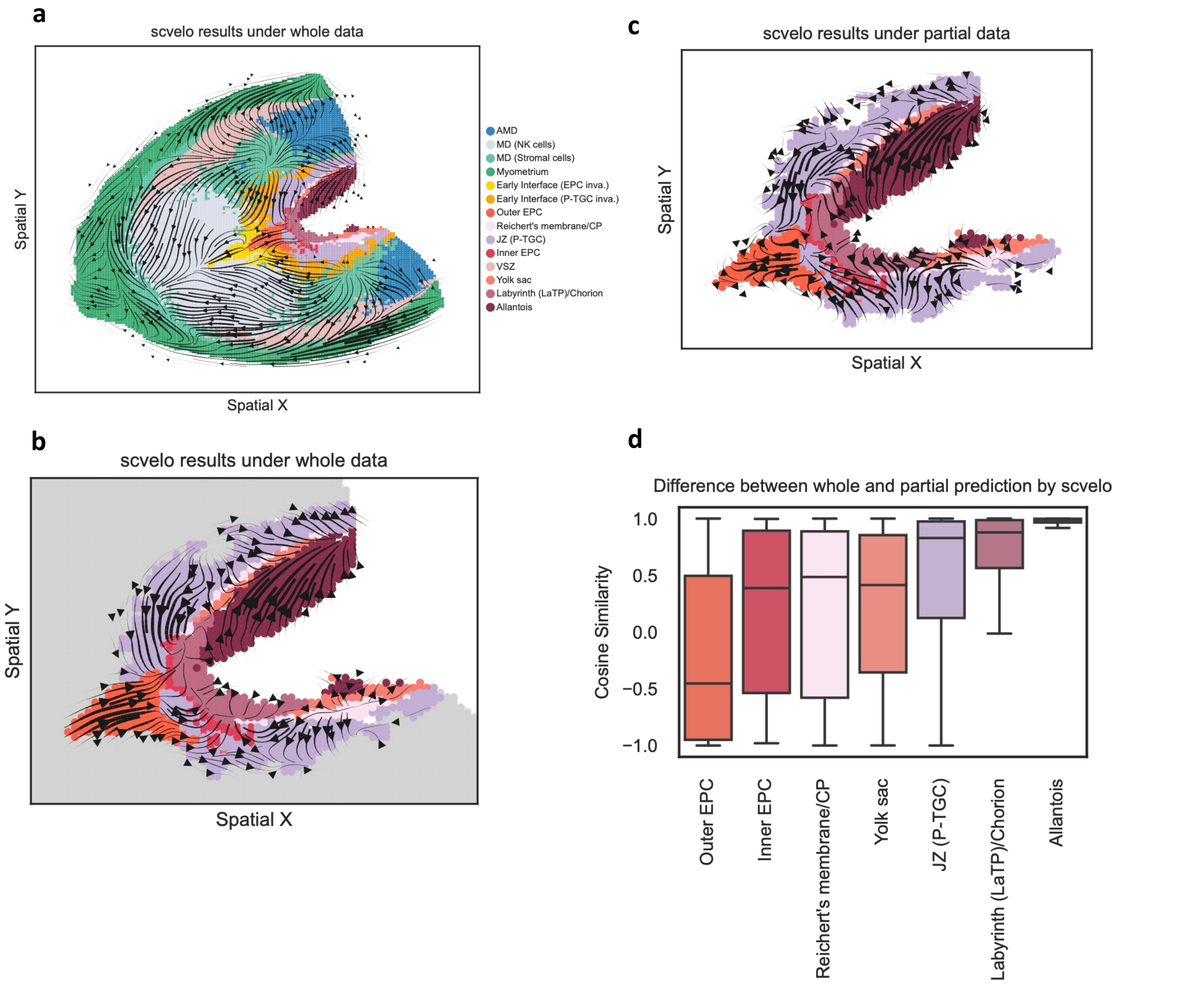

Supplementary Fig. 21. scVelo results on mouse placentation spatial transcriptomic data using whole and partial datasets

(a) RNA velocity projections from scVelo across the full spatial dataset, colored by regional annotation.

(b) RNA velocity projections computed using the full dataset, but visualized only for the embryonic region, colored by regional annotation.

(c) RNA velocity projections computed and visualized exclusively within the embryonic region, colored by regional annotation.

(d) Cosine similarity comparison between velocity fields derived from whole-data-based (as in b) and partial-data-based (as in c) analyses, across annotated regions.

Note: Panel (a) includes 13,258 spatial transcriptomic spots.

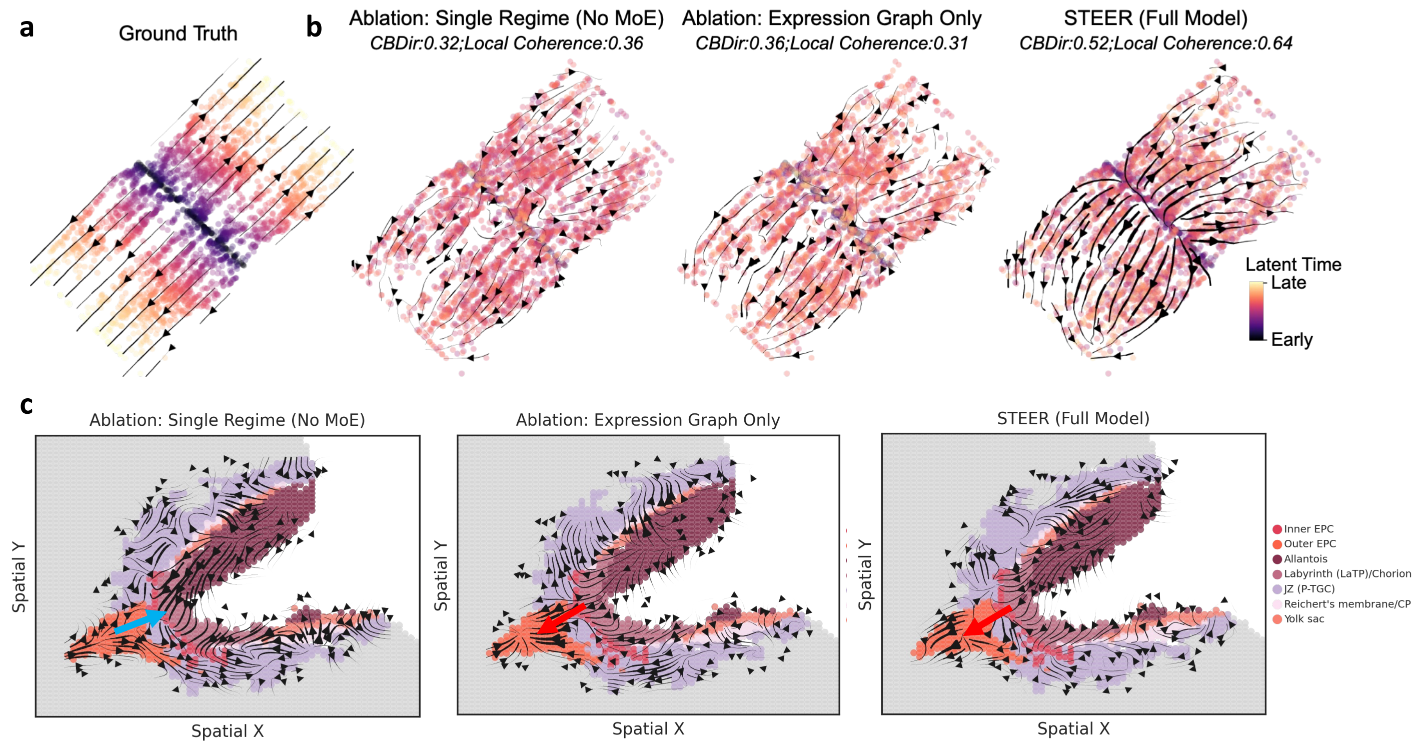

Supplementary Fig. 22. Component ablation of STEER on spatial simulations and mouse placentation data.
(a) Ground-truth velocity fields and latent time from TopoVelo spatial simulations. Colors indicate simulated time, and arrows represent local velocity directions. **(b)** Ablation analysis on spatial simulations. Left, single-regime model without MoE; middle, expression-only graph without spatial proximity while retaining MoE; right, STEER full model integrating expression- and spatially informed graph attention with multi-regime kinetic modeling. Velocity fields are visualized using identical parameters. CBDir and Local Coherence scores are shown below each panel.
(c) Ablation analysis on mouse placentation data at the inner-outer ectoplacental cone (EPC) boundary under the same three model configurations as in (b). Colors indicate annotated placental regions, and arrows denote inferred local velocity directions.

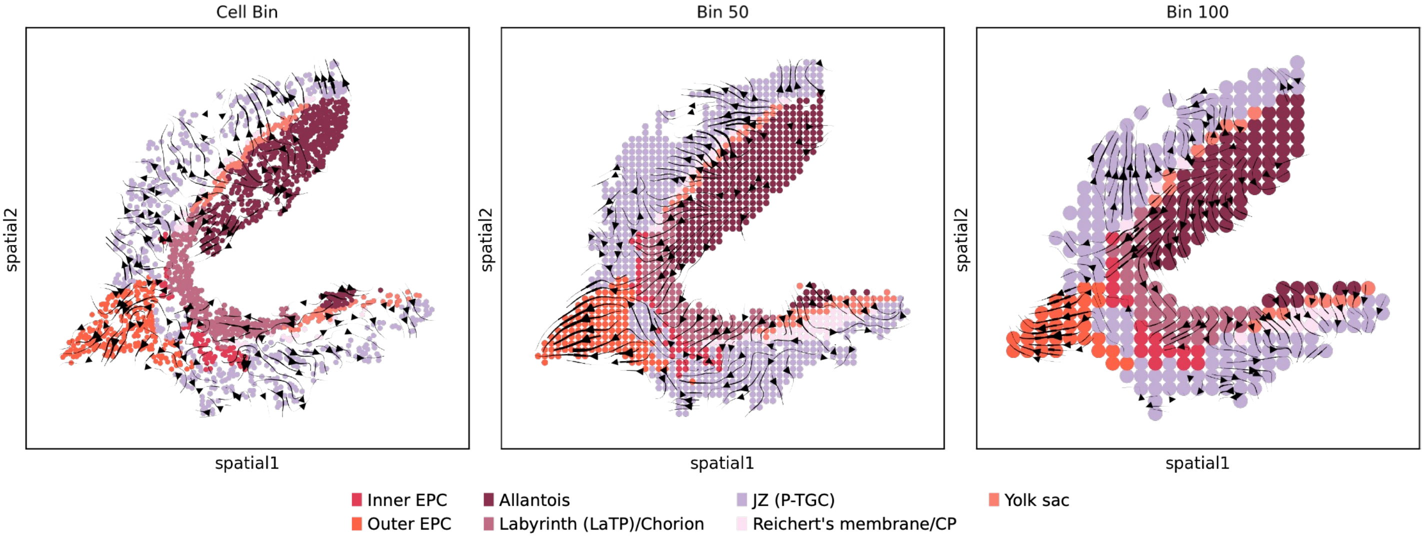

Supplementary Fig. 23. Consistent recovery of key spatial developmental processes across different Stereo-seq resolutions.

STEER was applied to the same mouse placentation dataset represented at three spatial resolutions: Cell-bin (near single-cell), Bin-50, and Bin-100. Across all resolutions, STEER consistently recovers two hallmark developmental processes: (i) the directional progression from the allantois toward the chorion, and (ii) the transition from Inner ectoplacental cone (Inner EPC) to Outer ectoplacental cone (Outer EPC). For each resolution, the model was trained on the full dataset, and the same anatomical region is shown here as a zoom-in view for direct comparison. Identical visualization settings were used across all panels.

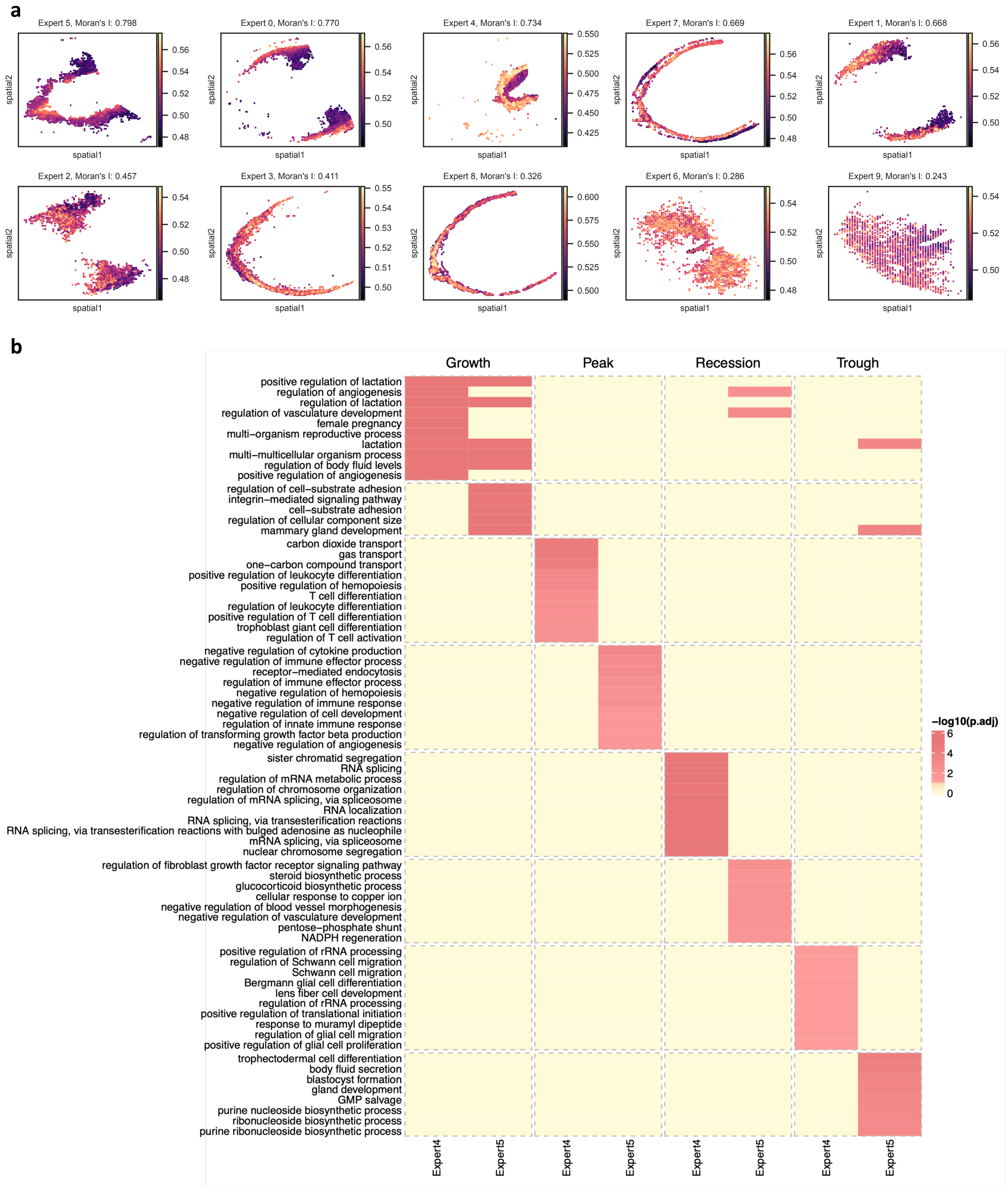

Supplementary Fig. 24. Kinetic Time Predictions Across All Experts and Pathway Enrichment Analysis of Time-Variable Genes Associated with Maternal-Fetal Interface Experts(4 and 5)

(a) Predicted kinetic time values for all experts. Spatial autocorrelation of these time values is quantified by Moran's I. **(b)** Pathway enrichment analysis of time-variable genes from Experts 4 and 5—including Growth, Peak, Recession, and Trough patterns. Bar color indicates $-\log_{10}$ adjusted *p*-values, with significance assessed using the Benjamini–Hochberg correction.

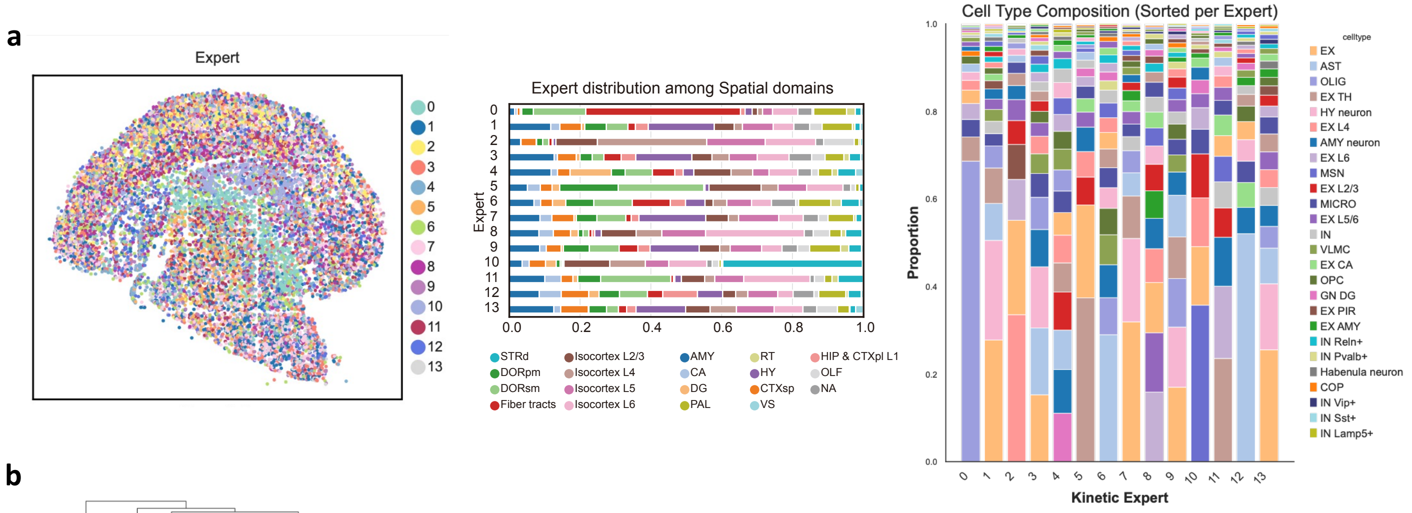

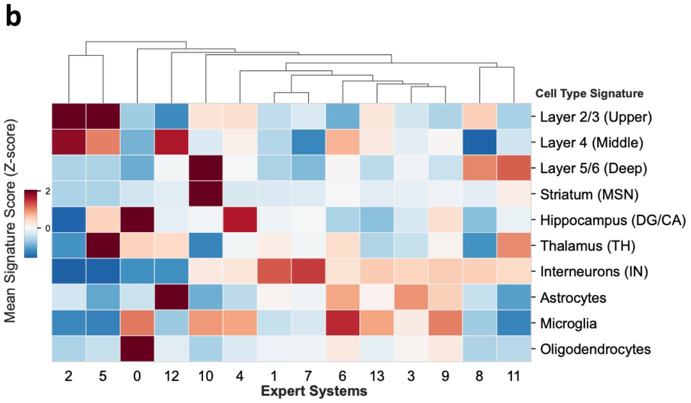

Cell Type Signature	Marker Genes
Layer 2/3 (Upper)	*Cux2, Calb1, Lamp5, Satb2*
Layer 4 (Middle)	*Rorb, Rspo1, Thsd7a*
Layer 5/6 (Deep)	*Bcl11b, Fezf2, Tle4, Foxp2, Syt6*
Striatum (MSN)	*Ppp1r1b, Drd1, Drd2, Penk, Tac1*
Hippocampus (DG/CA)	*Prox1, Wfs1, Fibcd1*
Thalamus (TH)	*Gbx2, Prkcd, Tcf7l2*
Interneurons (IN)	*Gad1, Gad2, Pvalb, Sst, Vip*
Astrocytes	*Aqp4, Slc1a2, Gfap, Aldoc*
Microglia	*P2ry12, C1qa, C1qb, Cx3cr1*
Oligodendrocytes	*Mbp, Plp1, Mog, Mag, Pdgfra, Cspg4*

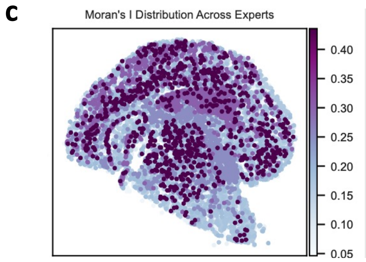

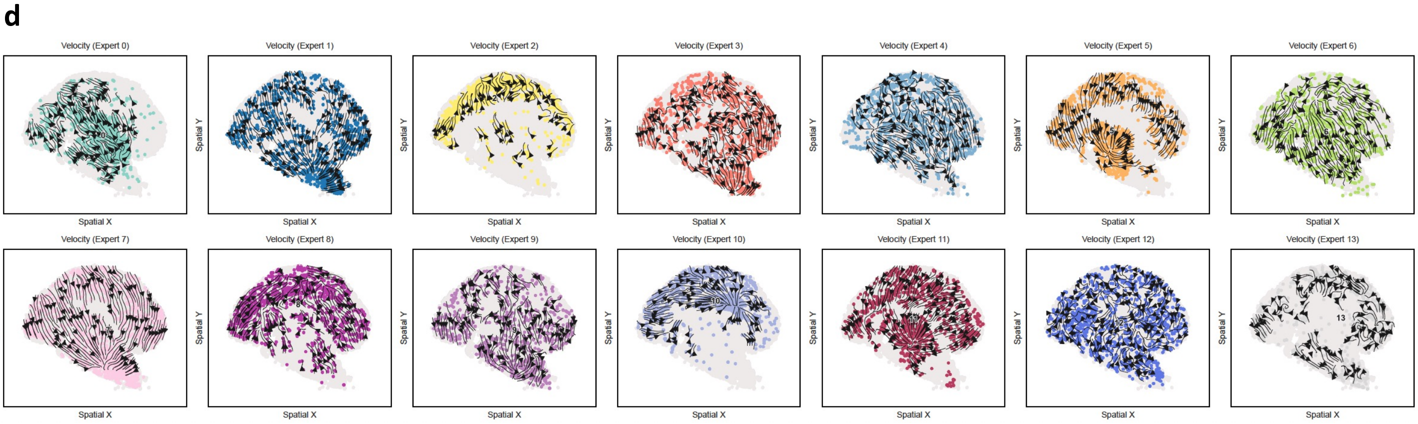

Supplementary Fig. 25. STEER resolves regionally overlapping but kinetically distinct dynamics in the mature mouse brain

(a) Left panel shows the spatial distribution of STEER-assigned expert systems across the adult mouse hemibrain. Middle/Right panels show the stack bar plot for spatial domains and cell type composition for each expert.

(b) Heatmap showing module scores for curated cell-type signatures computed from marker-gene sets (listed alongside) and summarized across STEER kinetic experts. Signatures include upper-layer (L2/3), middle-layer (L4), deep-layer (L5/6) excitatory programs, striatal medium spiny neurons (MSN), hippocampal (DG/CA) and thalamic excitatory programs, interneurons, and major glial lineages (astrocytes, microglia, oligodendrocyte/OPC). Scores are averaged within each expert, providing a compact view of expert composition and region-/cell-type–associated transcriptional programs. **(c)** Spatial distribution of Moran's I values for kinetic time within each expert, quantifying varying degrees of spatial-temporal coherence. **(d)** RNA velocity fields separated by expert assignment, visualizing distinct dynamic trajectories. The final panel presents principal component (PC) space embedding based on kinetic parameters, capturing expert-specific dynamical signatures.

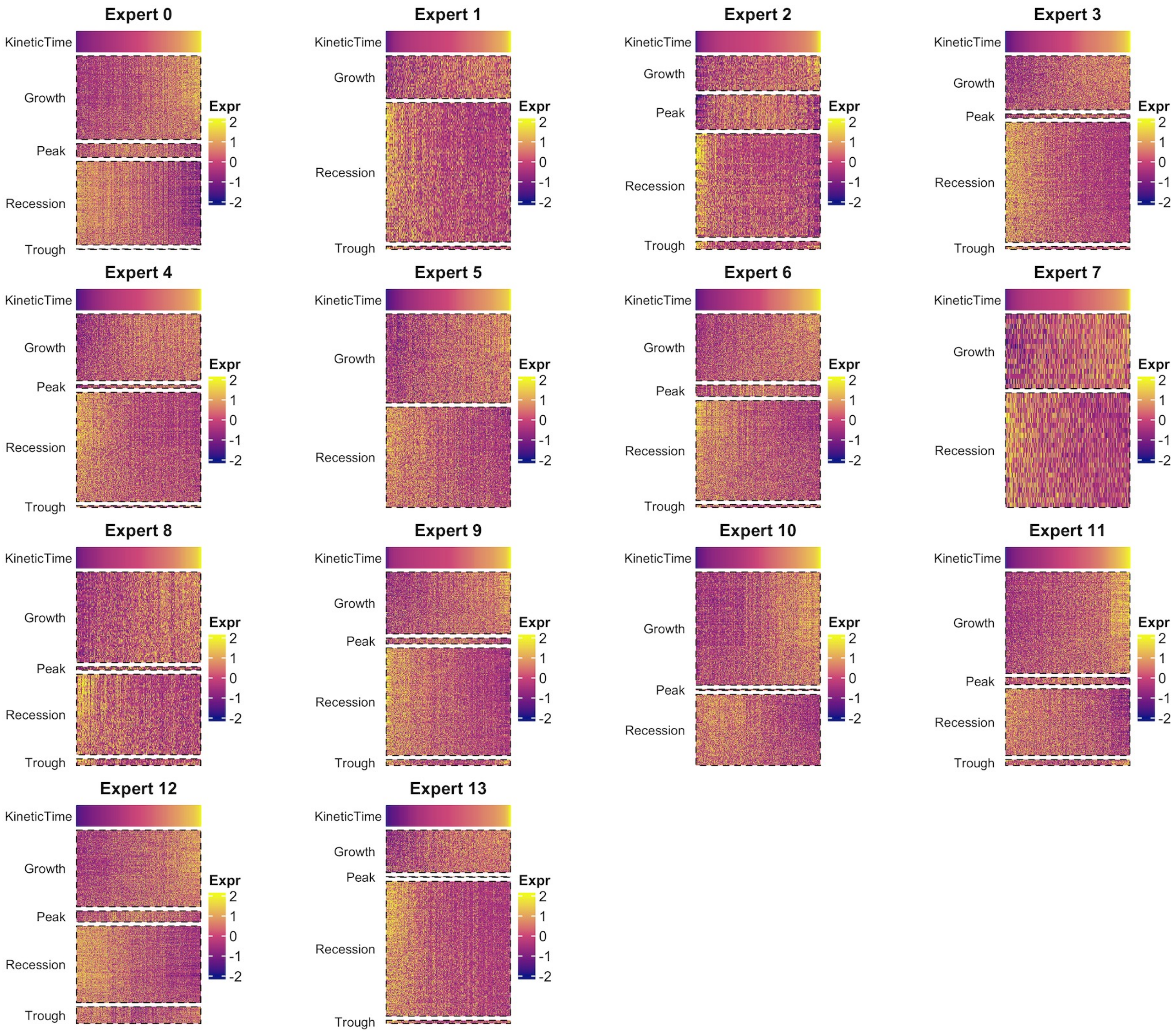

Supplementary Fig. 26. Temporal gene programs that characterize STEER experts in the adult mouse hemibrain

Heat-map shows genes whose expression changes significantly along each expert-specific kinetic time. Columns are single cells ordered from early to late kinetic time; rows are genes, grouped into four temporal patterns (Growth, Peak, Recession and Trough). Color indicates z-score-normalized expression.

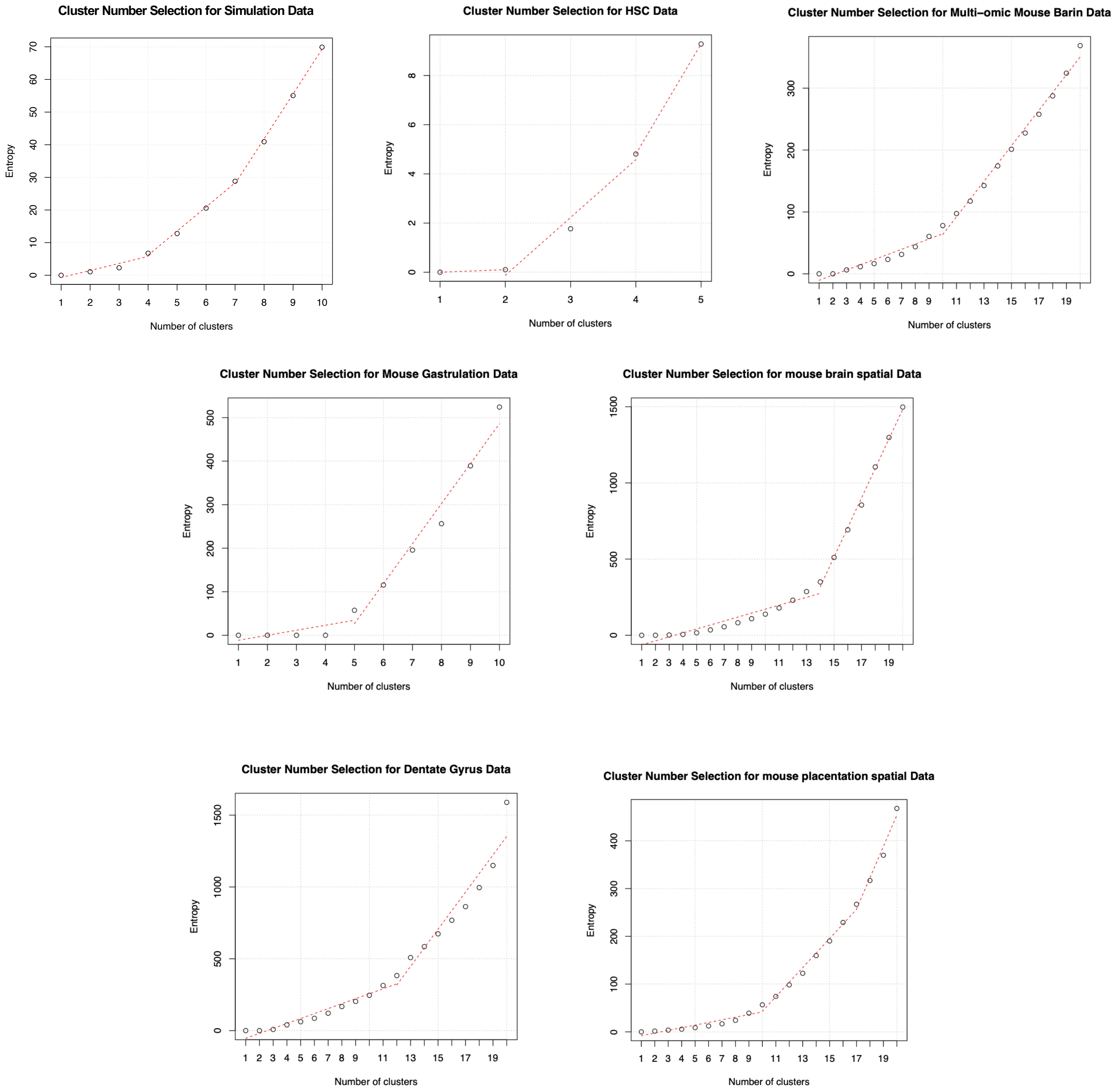

Supplementary Fig. 27. Expert number selection across datasets

Each data point represents the entropy value corresponding to a specific number of clusters. Red lines indicate linear fitting of the entropy values. The optimal number of clusters for each dataset was determined by identifying the first elbow point in the entropy curve—defined as the point preceding the onset of an upward trend.

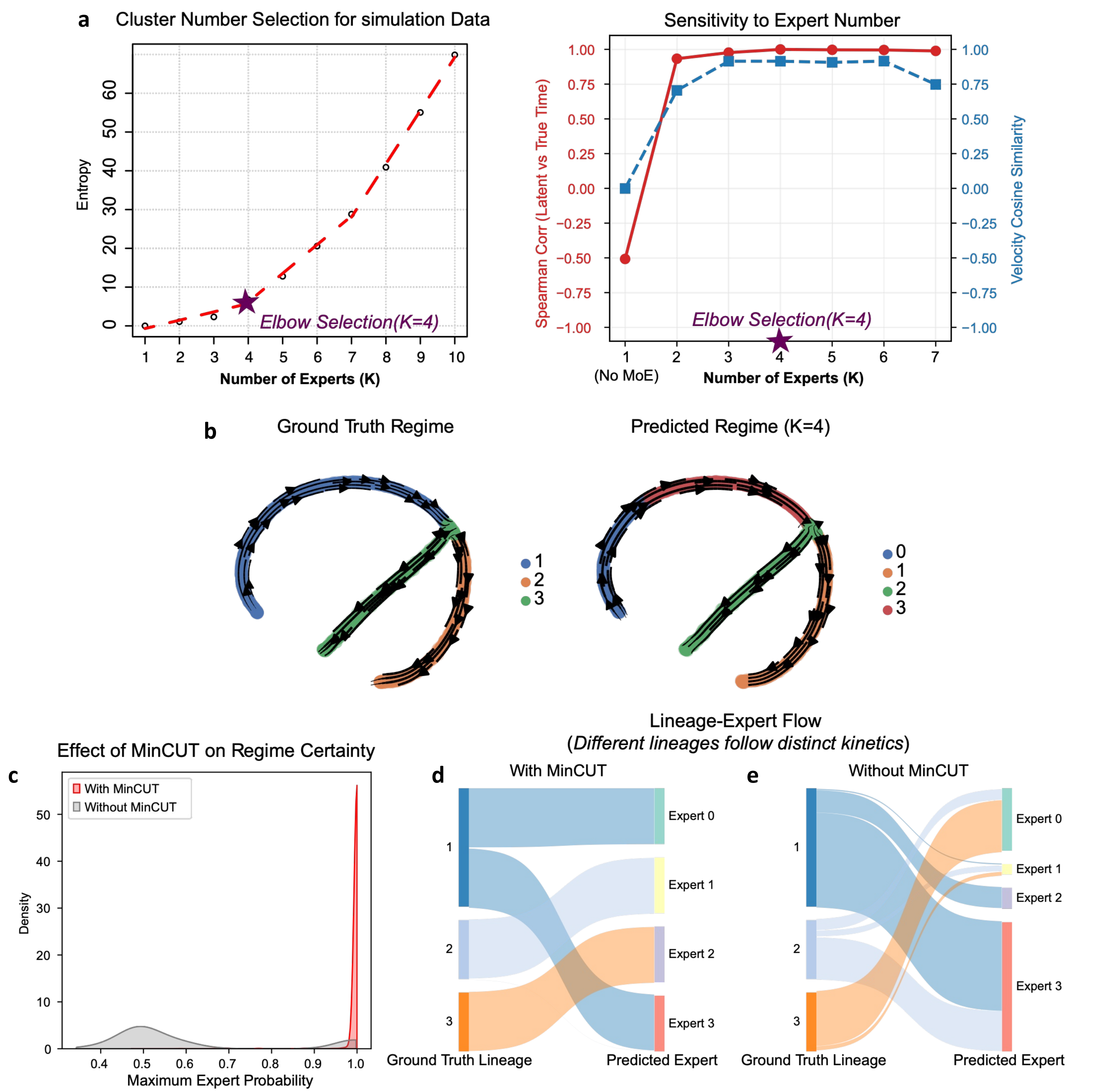

Supplementary Fig. 28. Robustness to expert number and effect of MinCUT regularization.

(a) Left: Entropy–elbow curve for selecting the number of experts K in the synthetic dataset, with the selected value K=4 indicated. Right: Sensitivity of velocity accuracy (cosine similarity) and latent-time reconstruction (Spearman correlation) to the number of experts. Performance remains stable for values of K near the selected optimum, with degradation observed only under substantial under-specification.

(b) Velocity streamplots showing high visual consistency between the ground truth regime and the STEER-predicted field (K=4).

(c) Expert assignment certainty. MinCUT regularization (red) enforces high-confidence assignments (peaking near 1.0) compared to the ambiguous distribution without it (grey).

(d, e) Lineage-to-expert mapping. MinCUT ensures clear functional specialization (d), while its exclusion results in redundant and diffuse mappings **(e)**.

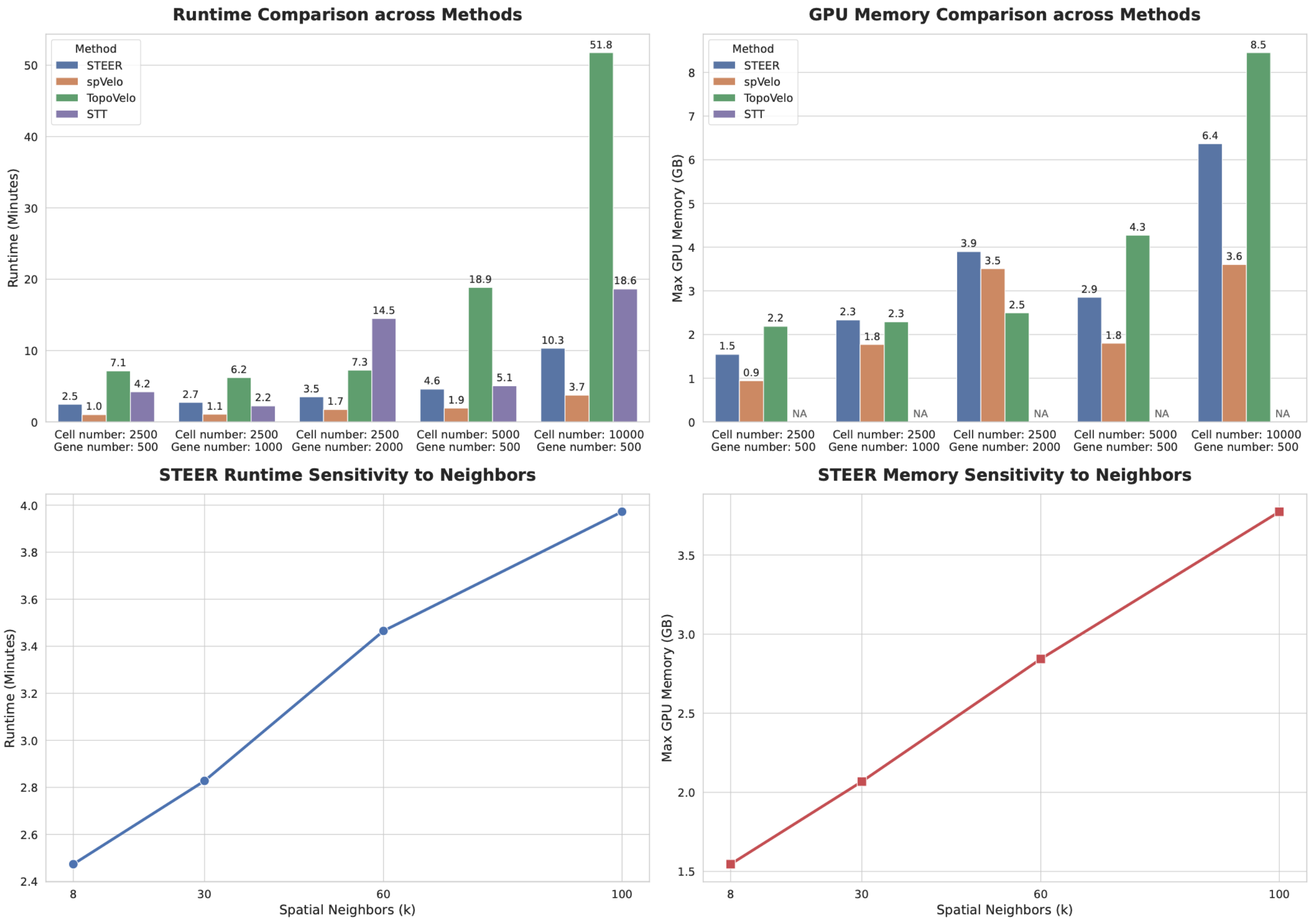

Supplementary Fig. 29. Computational cost and scalability on simulated spatial transcriptomics data.
Top, end-to-end wall-clock runtime (minutes; including preprocessing) and peak GPU memory (GB) for STEER, spVelo, TopoVelo and STT across increasing dataset sizes (number of cells) and feature sizes (number of genes). All GPU-based methods were run on a single NVIDIA L20 GPU (48 GB). STT is CPU-only and therefore GPU memory is reported as NA. Bottom, sensitivity of STEER to spatial graph density: end-to-end runtime and peak GPU memory as a function of the number of spatial neighbors k on a fixed simulated dataset (2,500 cells; 500 genes), showing a monotonic increase with k.
