# Supplementary material for: STEER: decoupling kinetics with Spatial-Temporal Explainable Expert model for RNA velocity inference
Source: Natl Sci Rev. 2026 Mar 30;13(9):nwag199. doi: 10.1093/nsr/nwag199 (PMC13220760; doi:10.1093/nsr/nwag199)
Supplement: nwag199_Supplemental_Files [file nwag199_supplemental_files.zip › Supplementary Methods and Notes.pdf]

STEER: Decoupling kinetics with Spatial–Temporal Explainable Expert Model for RNA velocity inference

Zhiyuan Liu[1,2], Yaru Li[3], Dafei Wu[1], Weiwei Zhai *[1,2,4] and Liang Ma †[1,2]

[1]State Key Laboratory of Animal Biodiversity Conservation and Integrated Pest Management, Institute of Zoology, Chinese Academy of Sciences, Beijing, 100101, China
[2]University of the Chinese Academy of Sciences, Beijing, 100049, China
[3]Department of Automation, Tsinghua University, Beijing, 100084, China
[4]Center for Excellence in Animal Evolution and Genetics, Chinese Academy of Sciences, Kunming, 650223, China

Supplementary Methods

Model specification for expert-resolved RNA velocity

STEER is a deep learning framework for RNA velocity inference that integrates a spatially informed graph-attention autoencoder (GAAE) and a kinetically guided mixture-of-experts (MoE) model to estimate cell- and gene-specific transcriptional kinetics. The model takes as input both unspliced and spliced transcript counts, together with a unified cell–cell graph constructed from transcriptional similarity and, when available, spatial proximity. The GAAE learns a low-dimensional embedding of cell states that preserves local context. A continuous latent-time variable is learned as a mapping from the latent state and is regularized to align with local transcriptional tendencies. To capture kinetic heterogeneity, a graph-constrained gated MoE module assigns cells to a set of discrete transcriptional regimes, each modeled by a dedicated expert network. This architecture enables flexible and interpretable modeling of complex, multi-regime transcriptional dynamics.

Generalized transcription kinetics. RNA velocity is modeled using a gene-wise system of first-order ordinary differential equations describing the temporal dynamics of unspliced and spliced transcripts:

$$\begin{cases} \dfrac{dU_g}{dt} = \alpha_g(\mathbf{x}, t) - \beta_g(\mathbf{x}, t)U_g, \\ \dfrac{dS_g}{dt} = \beta_g(\mathbf{x}, t)U_g - \gamma_g(\mathbf{x}, t)S_g, \end{cases}$$

where U_g and S_g denote the unspliced and spliced mRNA abundance for gene g, and $\mathbf{x} \in \mathbb{R}^{2G}$ represents the concatenated transcriptomic state across G genes. The kinetic parameters α_g, β_g, and

*Corresponding author: weiweizhai@ioz.ac.cn
†Corresponding author: maliang@ioz.ac.cn

γ_g (transcription, splicing, and degradation rates, respectively) are allowed to vary with both cell state and latent time, enabling the model to capture state-dependent and non-stationary dynamics.

To enhance identifiability and reduce over-parameterization, STEER constrains these dynamics via a regime-based decomposition inspired by Waddington's epigenetic landscape. Specifically, cells are mapped via $C(\mathbf{z}) \in \{1, 2, \ldots, K\}$ to one of K discrete kinetic regimes, within which the rate functions are assumed to vary smoothly with latent time:

$$\begin{cases} \dfrac{dU_g}{dt} = \alpha_g(C(\mathbf{z}), t) - \beta_g(C(\mathbf{z}), t)U_g, \\ \dfrac{dS_g}{dt} = \beta_g(C(\mathbf{z}), t)U_g - \gamma_g(C(\mathbf{z}), t)S_g. \end{cases}$$

This regime-aware parameterization enables STEER to disentangle overlapping kinetic programs while preserving continuous temporal trajectories within each regime.

Model architecture. The GAAE module encodes the concatenated unspliced–spliced expression profiles into a latent representation $\mathbf{z}_i$ for each cell i, leveraging attention mechanisms over a joint transcriptomic–spatial graph (Fig.1a–b). A time encoder then assigns each cell a latent kinetic time t_i, regularized to vary smoothly across the manifold and to correlate with local transcriptional tendency. The combined representation $(\mathbf{z}_i, t_i)$ is routed through a soft-gated MoE layer comprising K expert networks (Fig.1c). Each expert specializes in modeling a distinct transcriptional regime and outputs a triplet of kinetic rates $(\alpha_{i,g}, \beta_{i,g}, \gamma_{i,g})$ for every gene g and cell i.

Expert assignments are refined via a MinCut-based regularization that promotes intra-regime coherence and inter-regime separation within the learned cell graph. Velocity vectors $\mathbf{v}_{i,g} = [\frac{dU_{i,g}}{dt}, \frac{dS_{i,g}}{dt}]^T$ are derived from these rate estimates and compared against finite-difference estimates $\mathbf{\Delta}_{j,g} = [U_{j,g} - U_{i,g}, S_{j,g} - S_{i,g}]^T$ computed over temporally forward neighbors $\mathcal{N}_i^+ = \{j : t_j \geq t_i\}$, enforcing dynamic consistency during training.

Training strategy. STEER is trained in three sequential stages:

(1) *Initialization.* A preliminary GAAE is trained on highly variable genes to learn an initial latent representation and unsupervised cell clusters. These clusters capture global transcriptional structure and are used both filter out uninformative genes and to infer local transcriptional tendencies. In addition, they provide a coarse prior for subsequent expert assignment.

(2) *Embedding refinement.* The GAAE is retrained on the selected gene set with the kinetic time and temporal regularization enabled, yielding a temporally coherent and spatially informed embedding that serves as initialization for the subsequent dynamical learning phase.

(3) *Dynamical learning.* The full model, comprising the MoE module, is trained end-to-end to jointly optimize expert routing, cell- and gene-specific kinetic parameters, and velocity consistency across temporally ordered neighborhoods.

Detailed descriptions of the model components, including the GAAE and MoE architectures, temporal regularization, local transcriptional tendency inference, and loss formulations, are provided in the following subsections and in the Supplementary Notes.

Cell-Context learning by Graph-Attention Auto Encoder

Let $\mathcal{I} = \{1, 2, \ldots, I\}$ denote the set of cells and $\mathcal{G} = \{1, 2, \ldots, G\}$ represent the assayed genes. Define $\mathbf{U}_{I\times G}$ as the matrix of unspliced counts and $\mathbf{S}_{I\times G}$ as the matrix of spliced counts.

Data preprocessing and graph construction Unspliced ($\mathbf{U} \in \mathbb{R}^{I \times G}$) and spliced ($\mathbf{S} \in \mathbb{R}^{I \times G}$) count matrices were each column-normalized by their maximum value across I cells and then concatenated into

$$\mathbf{X} \;=\; \big[\mathbf{U} \,\|\, \mathbf{S}\big] \;\in\; \mathbb{R}^{I \times (2G)},$$

where each row $\mathbf{x}_i$ ($i = 1, \ldots, I$) encodes cell i. We constructed a cell–cell graph by taking, for each cell i, its k-nearest neighbors based on cosine similarity over $\mathbf{x}_i$. When spatial coordinates were available, the final adjacency was formed by the union of expression-based k-NN and spatial-based k-NN; otherwise, only expression-based neighbors were used (see Supplementary Note 1 for details). Denote this neighborhood set by $\mathcal{N}(i)$.

Encoder architecture A two-layer Graph Attention Network (GAT) projects $\mathbf{X}$ into a low-dimensional latent space. In the first GAT layer, attention coefficients $\{\alpha_{ij}\}$ are computed via a sigmoid-normalized scoring function (details in Supplementary Note 1), and each cell's hidden vector is

$$\mathbf{h}_i^{(1)} \;=\; \sigma\Big(\sum_{j \in \mathcal{N}(i)} \alpha_{ij}\, \mathbf{W}^{(1)}\, \mathbf{x}_j^T\Big),$$

where $\mathbf{W}^{(1)} \in \mathbb{R}^{d_1 \times (2G)}$ is trainable and $\sigma(\cdot)$ denotes Exponential Linear Units (ELU) activation. The second (linear) layer produces a d_2-dimensional latent embedding via

$$\mathbf{h}_i^{(2)} \;=\; \mathbf{W}^{(2)}\, \mathbf{h}_i^{(1)}, \quad \mathbf{W}^{(2)} \in \mathbb{R}^{d_2 \times d_1}.$$

Decoder architecture To reconstruct the original concatenated features, two decoder layers mirror the encoder with tied weights and attention. In the first decoder layer, the same $\{\alpha_{ij}\}$ are reused:

$$\mathbf{h}_i^{(3)} \;=\; \sigma\Big(\sum_{j \in \mathcal{N}(i)} \alpha_{ij}\, \mathbf{W}^{(2)\,T}\, \mathbf{h}_j^{(2)}\Big),$$

where $\mathbf{W}^{(2)\,T} \in \mathbb{R}^{d_1 \times d_2}$ reverses the encoder's second-layer projection. In the final layer, the reconstruction is

$$\widehat{\mathbf{x}}_i \;=\; \mathrm{sigmoid}\big(\mathbf{W}^{(1)\,T}\, \mathbf{h}_i^{(3)}\big),$$

so that $\widehat{\mathbf{x}}_i \in [0, 1]^{2G}$ matches the normalized input range.

All parameters and attention weights in this phase are optimized by minimizing a combined objective: a mean-squared-error reconstruction loss between $\mathbf{x}_i$ and $\widehat{\mathbf{x}}_i$, and a latent-space similarity regularization that encourages neighboring embeddings $\mathbf{h}_i^{(2)}$ to be close (see Supplementary Note 1). During the Cell-Context Learning phase, these two terms form the primary objective. To warm up the Mixture-of-Experts (MoE) gating network in later stages, we also introduce a MinCUT clustering loss; its detailed formulation and settings are provided in the MoE section and Supplementary Note 1.

Gene selection for dynamic learning and local transcriptional tendency inference

To identify genes informative for modeling transcriptional dynamics, we computed the Shannon entropy of predicted cluster labels across discretized spliced and unspliced expression space. Since these clusters capture global transcriptomic structure, genes exhibiting cluster-specific expression

patterns (i.e., low entropy) were selected for downstream dynamic modeling (see Supplementary Note 2).

For each selected gene, we performed quadratic B-spline fitting on unspliced and spliced counts within each cluster. Based on the curvature of these fitted trajectories in the unspliced–spliced phase space, cells were assigned local transcriptional tendencies, reflecting either upregulation or downregulation. This classification leverages the observation that transcriptional directionality induces asymmetric curvature in phase portraits. While this initialization relies on the assumption that transcriptional trends are locally monotonic, this constraint is applied within clusters rather than globally, allowing the model to flexibly accommodate branching or nonlinear dynamics. Importantly, STEER does not require all genes to exhibit reliable curvature trends—only a subset of coherent genes is sufficient to guide early time inference. To further enhance robustness, we aggregate directional signals across genes and treat these regulatory states as soft priors, which are iteratively refined or down-weighted during training. Genes with inconsistent or non-monotonic dynamics are automatically excluded from contributing to time supervision as learning progresses (see Supplementary Note 2).

Dynamic systems fitting framework

To capture heterogeneous transcriptional kinetics across the latent space, STEER augments the GAAE embedding with a Mixture-of-Experts (MoE) framework. A single global model often fails to represent diverse regulatory programs, especially in highly heterogeneous systems. Instead, the MoE partitions the latent manifold into K kinetic regimes (expert selection detailed in Supplementary Note 3), with a soft gating mechanism assigning each cell a distribution over expert networks to enable smooth transitions between regimes. While the gating mechanism is soft during training, assigning each cell a probability distribution over experts, we interpret the expert with the highest responsibility as the cell's primary regime during downstream analysis.

This modular design enables each expert to specialize in a distinct dynamical system, reducing interference across unrelated kinetics and improving the local estimation of transcription (α), splicing (β), and degradation (γ) rates. A cell-specific latent time variable further imposes global temporal directionality, guiding learning toward biologically coherent trajectories.

Time-prediction network. Each embedding $\mathbf{z}_i \in \mathbb{R}^D$ is mapped to kinetic time $t_i \in (0,1)$:

$$t_i = \sigma_{\text{sigmoid}}\big(\mathbf{W}_{\boldsymbol{\tau}}^{(2)}\, \sigma_{\text{LReLU}}(\mathbf{W}_{\boldsymbol{\tau}}^{(1)}\mathbf{z}_i + \mathbf{b}_{\boldsymbol{\tau}}^{(1)}) + \mathbf{b}_{\boldsymbol{\tau}}^{(2)}\big),$$

$$\mathbf{W}_{\boldsymbol{\tau}}^{(1)} \in \mathbb{R}^{H_\tau \times D}, \quad \mathbf{W}_{\boldsymbol{\tau}}^{(2)} \in \mathbb{R}^{1 \times H_\tau}.$$

Mixture-of-Experts. Expert k ($k = 1, \ldots, K$) is a four-layer MLP receiving $\mathbf{h}_k^{(0)} = [\mathbf{z}_i;\, t_i] \in \mathbb{R}^{d_0}$ ($d_0 = D + 1$):

$$\mathbf{h}_k^{(l+1)} = \sigma_{\text{LReLU}}\big(\mathbf{W}_{ek}^{(l)}\mathbf{h}_k^{(l)} + \mathbf{b}_{ek}^{(l)}\big), \quad l = 0, 1, 2,$$

$$\mathbf{W}_{ek}^{(l)} \in \mathbb{R}^{d_{l+1} \times d_l}.$$

A Sigmoid head outputs

$$\mathbf{e}_i^{(k)} = \sigma_{\text{sigmoid}}\big(\mathbf{W}_{ek}^{(3)}\mathbf{h}_k^{(3)} + \mathbf{b}_{ek}^{(3)}\big) \in (0,1)^{3G}, \quad \mathbf{W}_{ek}^{(3)} \in \mathbb{R}^{3G \times d_3}.$$

Gating and aggregation. Expert weights are produced by

$$\mathbf{g}_i = \mathrm{softmax}\big(\mathbf{W}_g^{(2)}\,\sigma_{\mathrm{LReLU}}(\mathbf{W}_g^{(1)}\mathbf{z}_i + \mathbf{b}_g^{(1)}) + \mathbf{b}_g^{(2)}\big)$$

$$\mathbf{W}_g^{(1)} \in \mathbb{R}^{d_g \times D}, \quad \mathbf{W}_g^{(2)} \in \mathbb{R}^{K \times d_g}.$$

Aggregated kinetics are

$$[\alpha_{ig}, \beta_{ig}, \gamma_{ig}] = \sum_{k=1}^{K} g_{ik}\,\mathbf{e}_{ig}^{(k)}.$$

Loss functions and training objective

Graph Attention Auto-Encoder loss. The GAAE model is trained to minimize the discrepancy between the input features and their reconstructed counterparts, while also preserving the structural integrity of the graph. The loss function is composed of a mean squared error term for node features, coupled with a graph regularization term that maintains similarity in latent representations among neighboring nodes. The loss function is formalized as:

$$\mathcal{L}_{GAAE} = \frac{1}{2IG}\sum_{i=1}^{I}\|\mathbf{x}_i - \hat{\mathbf{x}}_i\|_2^2 + \frac{1}{I}\sum_{i=1}^{I}\sum_{j\in\mathcal{N}(i)} -\log\left(\sigma(\mathbf{z}_i^T\mathbf{z}_j)\right),$$

where $\mathbf{x}_i$, $\hat{\mathbf{x}}_i$ and $\mathbf{z}_i$ are the original, reconstructed and latent features of node i, respectively, and σ denotes the Sigmoid function. This formulation enables effective capture of node features and preservation of graph topology, which facilitates a comprehensive analysis of the graph's intrinsic properties.

Expert-assignment loss. Expert assignment is constrained by a MinCUT objective, a dual-component loss that promotes distinct expert specialization by minimizing inter-cluster connectivity while maximizing intra-cluster cohesion:

$$\mathcal{L}_{\mathrm{MinCUT}} = \mathcal{L}_C + \mathcal{L}_O, \qquad \mathcal{L}_C = 1 - \frac{\mathrm{Tr}(\mathbf{C}^\top\mathbf{S}\mathbf{C})}{\mathrm{Tr}(\mathbf{C}^\top\mathbf{D}\mathbf{C})},\ \mathcal{L}_O = \left\|\frac{\mathbf{C}^\top\mathbf{C}}{\|\mathbf{C}^\top\mathbf{C}\|_F} - \frac{\mathbf{I}_K}{\sqrt{K}}\right\|_F.$$

Here $\mathbf{C} \in \mathbb{R}^{I\times K}$ are soft cluster weights with I cells and K experts; $\mathbf{S}$ and $\mathbf{D}$ denote the symmetrically normalised adjacency and degree matrices (see details in Supplementary Note 4).

Kinetic-time loss. Kinetic time inference is guided by two complementary regularization terms: (i) a local smoothness term that promotes temporal continuity along the latent manifold, and (ii) a consistency term that enforces alignment between latent time and gene-wise transcriptional directionality.

$$\mathcal{L}_{\mathrm{Time}} = \mathcal{L}_{\mathrm{Local\ Smooth}} + \mathcal{L}_{\mathrm{Expert\ Consistency}}$$

Local smoothness term. Inspired by t-SNE, we minimize the Kullback–Leibler divergence between Gaussian similarities p_{ij} in the GAAE-derived latent space and Student's t-distribution similarities q_{ij} in the one-dimensional time space (see Supplementary Note 5 for kernel details):

$$\mathcal{L}_{\text{Local Smooth}} = \text{KL}(p_{ij} \parallel q_{ij})$$

Expert consistency term. To incorporate biological priors into kinetic time estimation, STEER leverages transcriptional tendencies derived from unspliced–spliced curvature analysis within initial clusters (see Supplementary Note 2). These tendencies are encoded in a regulation matrix $\mathbf{R} \in \{-1, 0, 1\}^{K \times G}$, where R_{kg} denotes the dominant regulation sign of gene g within cluster k. During training, these clusters serve as an initialization for expert regimes, which are subsequently refined by the MoE architecture. The expert consistency loss encourages inferred time to align with these prior regulatory directions, while allowing dynamic adaptation over training.

$$\mathcal{L}_{\text{Expert Consistency}} = 1 - \frac{1}{K} \sum_{k=1}^{K} \frac{1}{\|\mathbf{R}_k\|_1 \sqrt{G}} \sum_{g=1}^{G} R_{kg} \cdot \text{corr}\big(t_k,\, u_{k,g}\big)$$

Here, t_k and $u_{k,g}$ denote the latent time vector and unspliced counts of gene g in cluster k, respectively. The normalization term $\|\mathbf{R}_k\|_1 = \sum_g |R_{kg}|$, together with $\sqrt{G}$, ensures that large experts or gene sets do not disproportionately dominate the loss. Because $\mathbf{R}$ encodes soft priors rather than hard constraints, STEER can iteratively down-weight inconsistent genes, enabling adaptive refinement of temporal trajectories throughout training (see Supplementary Note 5).

Velocity-consistency loss. To learn gene- and cell-specific kinetic rates, we compare the model-predicted instantaneous velocities with observed forward differences toward temporally later neighbors. For each cell i and gene g, we construct a *time-guided* neighborhood $\mathcal{N}_{i,g}$, retaining only those cells j with $t_j > t_i$ (see Supplementary Note 6 for details). Among these, we select the top-p neighbors that maximize cosine similarity in the (u, s)-plane, and define the per-cell–gene loss as:

$$\mathcal{L}_{\text{dyn}}^{i,g} = 1 - \frac{1}{p} \sum_{j \in \mathcal{N}_{i,g}^{p}} \cos\Big([u_{g,j} - u_{g,i},\, s_{g,j} - s_{g,i}], [v_{u,g}(t_i),\, v_{s,g}(t_i)]\Big),$$

where the predicted velocity components are given by $v_{u,g}(t_i) = \alpha_{ig} - \beta_{ig} u_{g,i}$ and $v_{s,g}(t_i) = \beta_{ig} u_{g,i} - \gamma_{ig} s_{g,i}$.

The overall dynamic loss is defined by averaging over all cells $\mathcal{I}$ and genes $\mathcal{G}$:

$$\mathcal{L}_{\text{dynamic}} = \frac{1}{|\mathcal{I}|\,|\mathcal{G}|} \sum_{i \in \mathcal{I}} \sum_{g \in \mathcal{G}} \mathcal{L}_{\text{dyn}}^{i,g}.$$

This loss enforces consistency between the learned kinetic rates and local transcriptomic changes along inferred temporal trajectories. To enhance training stability, we retain only the top-p future neighbors that yield the lowest cosine discrepancy with the predicted velocity. This heuristic suppresses noisy or ambiguous supervision and improves robustness across heterogeneous kinetic regimes.

Training objective. The full loss combines the above terms with the upstream GAAE reconstruction loss:

$$\mathcal{L}_{\text{total}} = \mathcal{L}_{\text{GAAE}} + \mathcal{L}_{\text{MinCUT}} + \mathcal{L}_{\text{Time}} + \mathcal{L}_{\text{dynamic}}.$$

Collectively, this objective ensures that inferred kinetics not only match transcriptomic trends locally, but also reflect globally coherent, temporally ordered dynamics.

To ensure stable optimization without requiring loss reweighting, we adopt a staged training strategy that first stabilizes representation learning, then progressively introduces time estimation and kinetic modeling. Specifically, STEER is trained in four phases: (1) initial GAAE pretraining to establish latent representations, (2) MinCUT regularization to refine regime structure, (3) warm-up initialization of latent time, and (4) joint training with all loss components. All loss terms are equally weighted throughout, ensuring robust and tuning-free performance across datasets.

Simulations

To benchmark STEER under controlled conditions, we constructed a synthetic dataset designed to reflect key challenges in RNA velocity inference, including monotonic maturation, lineage bifurcation, regime-specific kinetics, and transcriptional cycling. Cells were sampled from a branching manifold comprising one progenitor population and two divergent lineages, with unspliced–spliced dynamics governed by a first-order kinetic model:

$$\frac{du}{dt} = \alpha - \beta u, \qquad \frac{ds}{dt} = \beta u - \gamma s,$$

where α, β, and γ denote gene- and group-specific transcription, splicing, and degradation rates.

We embedded four canonical kinetic patterns—Sequential Shifts, Branching Trajectories, Multiple Independent Kinetics, and Classic Circular Pattern—into distinct gene subsets. Each gene's rate parameters were sampled from a uniform distribution around pattern-specific means. Trajectories were numerically integrated using an adaptive RK45 solver, and Gaussian noise was added to emulate technical variability.

The final dataset comprises 2,000 cells and 1,000 genes across multiple dynamic regimes. Full implementation details, parameter configurations, and generation code are available in Supplementary Note 7 and Supplementary Table 3.

Spatio-temporal Coherence Metric

To quantify the degree of spatial organisation in the inferred temporal dynamics, we computed a Moran's I statistic for each expert-specific kinetic system. For expert k, let $\mathcal{S}_k$ denote the set of spatial spots whose highest STEER responsibility is assigned to that expert k ($n_k = |\mathcal{S}_k|$). With t_i denoting the kinetic time of spot $i \in \mathcal{S}_k$ and (x_i, y_i) its spatial coordinates. Moran's I is defined as:

$$I_k \;=\; \frac{n_k}{W}\,\frac{\displaystyle\sum_{i\in\mathcal{S}_k}\sum_{j\in\mathcal{S}_k} w_{ij}\big(t_i-\bar{t}\big)\big(t_j-\bar{t}\big)}{\displaystyle\sum_{i\in\mathcal{S}_k}\big(t_i-\bar{t}\big)^2}, \qquad \bar{t} \;=\; \frac{1}{n_k}\sum_{i\in\mathcal{S}_k} t_i,$$

where w_{ij} is a spatial weight between spots i and j, computed using a 5-nearest-neighbour graph on Euclidean coordinates and row-normalized such that $\sum_j w_{ij} = 1$; $W = \sum_{i,j} w_{ij}$ is the normalizing constant. The index I_k ranges from –1 (strong spatial anti-correlation) to +1 (strong spatial clustering), with $I_k = 0$ indicating spatial randomness. In our data, all I_k values fall between 0 and 1. Higher values indicate that similar kinetic time values are spatially co-localized—i.e., stronger spatio-temporal coherence—while lower values may reflect spatial distributions that are not primarily governed by transcriptional kinetics, such as in cell migration, immune infiltration, or tissue remodeling.

Defining genes with Differential Kinetic Rates (DKGs)

STEER infers kinetic parameters for transcription (α), splicing (β), and degradation (γ) for each gene and each cell. To identify genes with differential kinetic rates (DKGs), we compared STEER-inferred kinetic parameters between expert-defined cell groups using statistical testing (see Supplementary Note 12 for details). Genes exhibiting a significant difference in at least one kinetic parameter were designated as DKGs.

DKGs were further classified based on the number of kinetic parameters showing significant differences: genes with one differential parameter were classified as single differential rate (SDR), those with two as double differential rate (DDR), and those with all three as triple differential rate (TDR).

Within DDR genes, we additionally distinguished coordinated (Sync) versus uncoordinated (Async) rate combinations. Sync patterns correspond to coordinated modulation of transcription and degradation that act in the same effective direction on mRNA abundance ($\uparrow \alpha$ with $\downarrow \gamma$, or $\downarrow \alpha$ with $\uparrow \gamma$), whereas other combinations lacking a clear monotonic effect on transcript accumulation or depletion were classified as Async.

Evaluation of velocity inference

In multi-kinetic simulations, we evaluated velocity inference accuracy by computing cosine similarity between the inferred and ground-truth velocity vectors. To sensitively probe performance under distinct kinetic regimes, we report cosine similarity at two resolutions (cell-level and gene-level) and separately for the spliced and unspliced velocity components.

Cell-level cosine similarity. For each cell $i \in \{1, \ldots, N\}$, let $\mathbf{v}_i^{(\cdot)} \in \mathbb{R}^G$ denote the velocity vector across G genes (with $(\cdot)$ indicating either the spliced or unspliced component). The cell-level cosine similarity is defined as

$$\text{cos\_cell}(i) = \frac{\left\langle \mathbf{v}_i^{(\cdot),\text{pred}},\ \mathbf{v}_i^{(\cdot),\text{gt}} \right\rangle}{\left\| \mathbf{v}_i^{(\cdot),\text{pred}} \right\| \ \left\| \mathbf{v}_i^{(\cdot),\text{gt}} \right\|}, \tag{1}$$

where $\mathbf{v}_i^{(\cdot),\text{gt}}$ and $\mathbf{v}_i^{(\cdot),\text{pred}}$ denote the ground-truth and inferred velocity vectors for cell i, respectively.

Gene-level cosine similarity. For each gene $g \in \{1, \ldots, G\}$, let $\mathbf{v}_g^{(\cdot)} \in \mathbb{R}^N$ denote the velocity values across N cells (again computed separately for spliced and unspliced components). The gene-level cosine similarity is defined as

$$\text{cos\_gene}(g) = \frac{\left\langle \mathbf{v}_g^{(\cdot),\text{pred}},\ \mathbf{v}_g^{(\cdot),\text{gt}} \right\rangle}{\left\| \mathbf{v}_g^{(\cdot),\text{pred}} \right\| \ \left\| \mathbf{v}_g^{(\cdot),\text{gt}} \right\|}. \tag{2}$$

For spatial simulations with 2D physical coordinates, we additionally quantified global performance in physical space using task-relevant metrics that capture directionality, temporal ordering, cross-boundary progression, and local smoothness (velocity accuracy, latent-time correlation, CB-Dir, and local coherence), as defined below.

Velocity Accuracy. Let $\mathbf{v}_i^{\text{pred}} \in \mathbb{R}^2$ and $\mathbf{v}_i^{\text{gt}} \in \mathbb{R}^2$ denote inferred and ground-truth velocity vectors projected to the physical coordinate system for cell i. We defined per-cell velocity accuracy as cosine similarity,

$$\text{Acc}_i = \frac{\left\langle \mathbf{v}_i^{\text{pred}},\ \mathbf{v}_i^{\text{gt}} \right\rangle}{\left\| \mathbf{v}_i^{\text{pred}} \right\| \ \left\| \mathbf{v}_i^{\text{gt}} \right\|}, \tag{3}$$

and summarized Acc_i across cells.

Latent time correlation. Latent time accuracy was assessed by Spearman rank correlation between inferred and ground-truth latent times, computed across all cells.

Cross-boundary directionality (CBDir). Following the TopoVelo simulation protocol, cells were partitioned into five temporal groups by discretizing the ground-truth generation time into equal-width bins, yielding labels $c_i \in \{0, 1, 2, 3, 4\}$. We considered consecutive transitions $\mathcal{E} = \{(0,1), (1,2), (2,3), (3,4)\}$. Let $\mathcal{N}(i)$ denote the set of spatial neighbors of cell i defined by a k-nearest-neighbor graph in physical space, and let $\mathcal{N}_v(i) = \{j \in \mathcal{N}(i) : c_j = v\}$ be the subset of neighbors that lie in the target temporal group v. For an edge $(u, v) \in \mathcal{E}$ and a cell i with $c_i = u$, we computed the mean cosine similarity between the inferred velocity $\mathbf{v}_i^{\text{pred}}$ and the displacement vectors from i to its cross-boundary neighbors:

$$s_i^{u \to v} = \frac{1}{|\mathcal{N}_v(i)|} \sum_{j \in \mathcal{N}_v(i)} \frac{\left\langle \mathbf{x}_j - \mathbf{x}_i,\ \mathbf{v}_i^{\text{pred}} \right\rangle}{\left\| \mathbf{x}_j - \mathbf{x}_i \right\| \ \left\| \mathbf{v}_i^{\text{pred}} \right\|}, \tag{4}$$

where $\mathbf{x}_i \in \mathbb{R}^2$ is the physical coordinate of cell i. The CBDir score for an edge (u, v) is the average of $s_i^{u \to v}$ over all source cells with $c_i = u$, and the overall CBDir score is the mean across all edges in $\mathcal{E}$.

Local coherence. To measure spatial smoothness of inferred dynamics, we computed, for each cell i, the mean cosine similarity between its inferred velocity and those of its spatial neighbors:

$$\text{LC}_i = \frac{1}{|\mathcal{N}(i)|} \sum_{j \in \mathcal{N}(i)} \frac{\left\langle \mathbf{v}_i^{\text{pred}},\ \mathbf{v}_j^{\text{pred}} \right\rangle}{\left\| \mathbf{v}_i^{\text{pred}} \right\| \ \left\| \mathbf{v}_j^{\text{pred}} \right\|}, \tag{5}$$

and summarized LC_i across cells.

Intra-Expert Transition flows calculation

To resolve spatial dynamics specific to distinct kinetic regimes, we compute coarse-grained transition flows within each expert system. Since STEER partitions cells into transcriptionally coherent regimes via expert assignments, this strategy enables us to isolate regime-specific spatial trajectories without cross-regime confounding.

We first compute cell-to-cell transition probabilities among the 30 nearest neighbors in latent space, following standard RNA velocity formulations[1,2]. For each expert, we restrict analysis to spots with maximal responsibility assigned to that expert, and aggregate transitions across spatial

regions. The top 10 most abundant regions are identified, and pairwise net flows are computed by subtracting reverse-direction probabilities.

Edges with low flow magnitude are omitted for clarity. The resulting antisymmetric flow matrix is visualized as a directed circular graph, with edge widths proportional to net flow strength and nodes indicating spatial region identity.

Preprocessing of Data

Erythroid Lineage of Mouse Gastrulation Data We obtained the scRNA-seq dataset (9,815 cells) from the scVelo website, comprising blood progenitors 1 and 2 as well as erythroid stages 1, 2, and 3. These cells were collected at embryonic stages E7.0, E7.25, E7.5, E7.75, E8.0, E8.25, and E8.5. Preprocessing followed standard scVelo procedures, using 100 nearest neighbors for smoothing (consistent with the CellDancer preprocessing parameters) to reduce transcriptional noise.

Human Hematopoiesis scNT-seq Data We downloaded the raw hematopoiesis data (1,947 cells) from Dynamo. Preprocessing and gene selection were performed according to Dynamo's recommendations, with smoothing parameters set to 100 nearest neighbors.

Mouse Developing Hippocampus Data We obtained data from the developing mouse hippocampus (18,213 cells) from scVelo, covering two time points (P0 and P5). The dataset includes multiple branching lineages, leading to astrocytes, oligodendrocyte precursor cells (OPCs), granule neurons, and pyramidal neurons. Preprocessing was conducted following standard scVelo procedures, with smoothing parameters set to 100 nearest neighbors.

10x Embryonic E18 Mouse Brain (Single-Cell Multi-omic Data) We obtained the raw data (4,881 cells) from the 10x Genomics website, while the preprocessed data and annotations were sourced from MultiVelo. ATAC-seq data were preprocessed following MultiVelo guidelines, with smoothing parameters set to 100 nearest neighbors.

Mouse Placentation Spatial Transcriptomic Data We retrieved bin-50 Stereoseq mouse placentation data (E8.5 S1; 13,631 cells) from the MPSTA database. Preprocessing was performed using 50 nearest neighbors for smoothing.

Mouse Brain Spatial Transcriptomic Data We utilized cell bin-level Stereoseq mouse brain data comprising 11,854 cells. Preprocessing involved a smoothing technique based on the 100 nearest neighbors.

Evaluation of predicted time for comparing methods

We evaluated all methods with published support for time prediction, including scvelo, VeloVI, CellDancer, and latentVelo. For a fair benchmarking of cell-specific time predictions, we directly used the predicted time outputs from methods such as STEER and latentVelo, which are capable of deriving cell-specific time values. For CellDancer, we employed the command `celldancer.pseudo_time`, and for scvelo and VeloVI, we followed the scvelo guidelines to derive gene-shared latent time.

Training and Implementation Details

All datasets were processed using a consistent STEER architecture and shared hyperparameter settings. Specifically, the GAAE uses a single-head GAT (no dropout) with a 512-dimensional hidden layer and a 128-dimensional latent embedding, with a symmetric decoder. The MoE gating network uses a 64-dimensional hidden layer, and each expert is a 3-layer MLP with widths 256/512/1024. The model was implemented in PyTorch and trained with GPU acceleration (1–2 NVIDIA RTX 3090 GPUs). A learning rate of 0.001 was used with the AdamW optimizer. Early stopping was used to prevent overfitting (default patience = 100 epochs in our benchmarks; we increase patience for more complex or noisier datasets when the validation objective continues to improve; see Supplementary Note 13)

For graph construction, we used an unweighted, symmetric k-nearest neighbors (KNN) graph based on cosine similarity of concatenated unspliced and spliced expression profiles, with $k = 30$ for all single-cell datasets. For spatial datasets, the graph was augmented with 8 spatially proximal neighbors and merged with the transcriptomic KNN graph; for irregular or sparse spatial layouts, we recommend increasing the number of spatial neighbors to ensure sufficient graph connectivity. For the scNT-seq and dentate gyrus datasets, only spliced counts were used for graph construction.

To reduce noise and enhance interpretability, we selected 1,000 genes with the lowest entropy scores (see Supplementary Note 2). We applied KNN smoothing using 100 neighbors by default; this parameter can be increased for noisier datasets. The number of experts K was selected automatically using our entropy-based criterion (Supplementary Note 3). For the local time–expression correlation metric used for temporal supervision, correlations were computed on unspliced counts by default; for Slide-seq data, where unspliced counts are highly sparse, we compute this metric on spliced counts to obtain a stable estimate. For the large-scale placentation spatial dataset, 70% of spots were randomly subsampled during training to reduce memory usage, and predictions were extrapolated to the full dataset.

STEER was trained using a four-stage schedule: (1) autoencoder pretraining to initialize latent representations; (2) additional epochs with MinCUT regularization to refine regime structure; (3) warm-up phase for latent time initialization; and (4) joint training with all loss terms enabled.

Supplementary Notes

Supplementary Note 1: Details in Cell-Context Learning Stage

Graph Attention Auto-Encoder Network Architecture Our model employs a Graph Attention Network (GAT) in an encoder-decoder format. Each node in the graph represents a cell, encapsulating gene expression by concatenating unspliced and spliced metrics.

Let $\mathcal{I} = \{1, 2, \ldots, I\}$ denote the set of cells and $\mathcal{G} = \{1, 2, \ldots, G\}$ represent the assayed genes. Define $\mathbf{U}_{I\times G}$ as the matrix of unspliced counts and $\mathbf{S}_{I\times G}$ as the spliced counts matrix.

Construction of the Adjacency Matrix The adjacency matrix, within the context of the graph attention framework, serves as a critical component for identifying potential candidates that can enhance cell representations by capturing relevant intercellular relationships. To expand the search space for these candidates, we combine the two adjacency matrices based on expression and spatial proximity. Specifically, the expression-based adjacency matrix $A_{\text{expression}}$ is constructed using a k-nearest neighbors (k-NN) approach based on cosine similarity of concatenated unspliced

and spliced gene expression profiles:

$$\mathcal{N}_{\text{expression}}(i) = \{i\} \cup \{j \in \mathcal{I} \mid j \text{ is one of the } k \text{ nearest neighbors of } i \text{ based on cosine similarity}\}.$$

Similarly, the spatial adjacency matrix A_{spatial} is constructed based on the physical spatial coordinates of the cells:

$$\mathcal{N}_{\text{spatial}}(i) = \{i\} \cup \{j \in \mathcal{I} \mid j \text{ is one of the } k \text{ nearest neighbors of } i \text{ based on spatial proximity}\}.$$

The final adjacency matrix A is obtained by taking the union of these two matrices:

$$A = A_{\text{expression}} \cup A_{\text{spatial}}.$$

In cases where only single-cell data is available, and spatial information is absent, the adjacency matrix is solely based on gene expression similarity:

$$A = A_{\text{expression}}.$$

This strategy expands the search space for relevant neighbors while mitigating spatial heterogeneity, enforcing local manifold continuity, and preserving velocity accuracy under low-coverage conditions. By integrating both spatial and transcriptional context, it yields a more coherent representation of cellular relationships and improves the quality of downstream analyses in single-cell and spatial transcriptomics.

Encoder: The encoder maps node features into a lower-dimensional latent space preserving essential biological information, structured as follows:

1. **Input Layer:** Normalizes and concatenates unspliced and spliced expression metrics as

$$\mathbf{X}_{I\times 2G} = (\mathbf{x}_i)_{i\in I} = \mathbf{U}_{I\times G} \| \mathbf{S}_{I\times G},$$

   where $\|$ denotes concatenation and normalization is done by the maximum value for each gene across cells, x_i represents the input features of cell i.

2. **First GAT Layer:** Maps input features to a hidden representation, the cell i in this layer 1 being represented by

$$\mathbf{h}_i^{(1)} = \sigma\left(\sum_{j\in\mathcal{N}(i)} \alpha_{ij}^{(1)} \mathbf{W}^{(1)} \mathbf{h}_j^{(0)}\right),$$

   where $\alpha_{ij}^{(1)}$ are attention coefficients between cell i and cell j, $\mathbf{W}^{(1)}$ is the trainable weight matrix, and σ is a nonlinear activation function. By treating expression profiles as initial cell embeddings, $\mathbf{h}_i^{(0)} = \mathbf{x}_i^T, i \in 0, 1, \ldots, I$

3. **Second GAT Layer:** Transforms the intermediate hidden features to latent features essential for downstream tasks, the embeddings of cell i in this layer is defined by

$$\mathbf{h}_i^{(2)} = \mathbf{W}^{(2)} \mathbf{h}_i^{(1)},$$

   with $\mathbf{W}^{(2)}$ as the trainable weight matrix.

Decoder: The decoder's role is to reconstruct the input features from the latent representations, effectively inversely mirroring the encoder's architecture to achieve robust embeddings learning:

1. **Third GAT Layer:** This layer reflects the attention coefficients from the first GAT layer to ensure that the embeddings' attention structure mirrors that found in the original expression space, thus conserving the biological relevance of cell interactions. The mathematical formulation of cell i in this layer is:

$$\mathbf{h}_i^{(3)} = \sigma\left(\sum_{j\in\mathcal{N}(i)} \alpha_{ij}^{(3)}\mathbf{W}^{(3)}\mathbf{h}_j^{(2)}\right),$$

   where $\alpha_{ij}^{(3)} = \alpha_{ij}^{(1)}$ is used to maintain consistent attention across layers, and $\mathbf{W}^{(3)} = \mathbf{W}^{(2)^T}$, enabling a reverse flow of feature transformations while preventing overfitting.

2. **Fourth GAT Layer:** The final layer of the decoder aims to accurately reconstruct the initial input features from the transformed embeddings, the reconstructed expression of cell i is represented by:

$$\hat{\mathbf{x}}_i = \sigma\left(\mathbf{W}^{(4)}\mathbf{h}_i^{(3)}\right),$$

   where $\mathbf{W}^{(4)} = \mathbf{W}^{(1)^T}$, σ is the Sigmoid activation function that ensuring output values are constrained between 0 and 1, optimizing the reconstruction quality and fidelity to the normalized data.

Attention Coefficients Calculation: The self-attention mechanism employed adjusts the importance of neighboring cells dynamically, facilitating nuanced learning of cellular relationships:

$$\alpha_{ij}^{(k)} = \frac{\exp\left(\text{Sigmoid}\left(\mathbf{v}^T[\mathbf{W}^{(k)}\mathbf{h}_i^{(k-1)}\|\mathbf{W}^{(k)}\mathbf{h}_j^{(k-1)}]\right)\right)}{\sum_{l\in\mathcal{N}(i)\cup\{i\}}\exp\left(\text{Sigmoid}\left(\mathbf{v}^T[\mathbf{W}^{(k)}\mathbf{h}_i^{(k-1)}\|\mathbf{W}^{(k)}\mathbf{h}_l^{(k-1)}]\right)\right)},$$

where $\mathbf{h}_i^{(k-1)}$ is the feature vector from the preceding layer, $\mathbf{W}^{(k)}$ is the specific weight matrix for that layer, and $\mathbf{v}$ is the trainable attention vector.

Loss Function for Graph Attention Auto-Encoder: The GAAE model is trained to minimize the discrepancy between the input features and their reconstructed counterparts, while also preserving the structural integrity of the graph. The loss function is composed of a mean squared error term for node features, coupled with a graph regularization term that maintains similarity in latent representations among neighboring nodes. The loss function is formalized as:

$$\mathcal{L}_{GAAE} = \frac{1}{2IG}\sum_{i=1}^{I}\|\mathbf{x}_i - \hat{\mathbf{x}}_i\|_2^2 + \frac{1}{I}\sum_{i=1}^{I}\sum_{j\in\mathcal{N}(i)} -\log\left(\sigma(\mathbf{z}_i^T\mathbf{z}_j)\right),$$

where $\mathbf{x}_i$, $\hat{\mathbf{x}}_i$ and $\mathbf{z}_i$ are the original, reconstructed and latent features of node i, respectively, and σ denotes the Sigmoid function. This formulation enables effective capture of node features and preservation of graph topology, which facilitates a comprehensive analysis of the graph's intrinsic properties.

Supplementary Note 2: Details in Gene selection for dynamic learning and Local transcriptional tendency inference

Using Entropy to Select Genes Related to Biological Processes We use Shannon entropy under the predicted label as a metric to select genes, extending the analysis to datasets lacking real-time or developmental labels. To calculate entropy, we partition the normalized (maximum normalized) unspliced and spliced values into five grids respectively. For each grid point, we calculate the entropy value for the predicted labels based on cell embeddings in GAAE phase by mclust.

Specifically, for each grid point, we count the number of cells assigned to each cluster to determine the probability p_k of each predicted cell type label. The mean entropy among the non-zero grid points represents the gene's entropy. Genes exhibiting the lowest entropy values (top 1000 genes) are subsequently selected. The Shannon entropy is calculated as:

$$H = -\sum_k (p_k \log(p_k))$$

where p_k is the probability of the cluster k at each grid point, derived from the counts of cells assigned to clusters based on the cluster matrix C. The mean entropy for each gene is calculated as:

$$H_{mean} = \frac{1}{N_{\text{nz}}} \sum_{i=1}^{N_{\text{nz}}} H_i$$

where N_{nz} is the number of non-zero grid points and H_i is the entropy at the i-th non-zero grid point.

Identification Local transcriptional tendency for Genes and Cell Populations After selecting genes in previous section, we focus on extract local transcriptional tendency of those genes exhibiting distinct unspliced–spliced (u–s) expression patterns. We hypothesize that, within each cell cluster, cells can be partitioned into two regulatory states—upregulation or downregulation—based on the convexity of gene expression trajectories.

To infer these states, we perform separate quadratic B-spline fittings on the spliced and unspliced counts of each gene within each cluster, computing the second derivative at each cell as a proxy for local transcriptional curvature. This separation addresses cases in which non-monotonic kinetics may lead to non-bijective u–s relationships. Statistical significance of curvature polarity is assessed using the Wilcoxon rank-sum test comparing cells with positive versus negative first derivatives.

If the convexities inferred from spliced and unspliced fits are directionally concordant—namely, positive in spliced and negative in unspliced curves (or vice versa)—we assign the corresponding regulatory label to the cluster. Specifically, if the majority of cells in a cluster exhibit positive convexity in spliced RNA and negative convexity in unspliced RNA, the gene is labeled as downregulated in that cluster. Conversely, the reverse configuration is interpreted as upregulation. In the case of discordant curvature directions, we compare significance levels and adopt the assignment with stronger statistical support. To mitigate the influence of outlier curvature estimates, we exclude the top and bottom 5% of convexity values when computing mean convexity per cluster. After inference, we derived the regulation matrix as $\mathbf{R}_{\text{init}} \in \{-1, 0, +1\}^{I \times G}$ (+1 for upregulation, −1 for downregulation, and 0 for uninformative genes).

These inferred regulatory states provide initial labels for dynamic systems fitting. To account for possible inaccuracies introduced at this stage, regulation labels are not fixed, but are adaptively updated throughout the training process, enabling the model to refine state assignments in response to the learned transcriptional dynamics.

Supplementary Note 3: Expert Numbers Selection

We adopted a data-driven strategy to determine the optimal number of experts (denoted as K) for each dataset. After retaining 2,000 highly variable genes, we performed PCA and used the top 30 principal components as input features. An entropy-based criterion was applied to estimate the number of transcriptional regimes[3]. Specifically, we selected the first elbow point in the entropy curve—prior to any upward trend—as the optimal cluster number, which we then assigned as the number of experts (Supplementary Fig.27). By default, the search range was set from 1 to 20 clusters.

For datasets with relatively few cells or simple, near-linear developmental trajectories, we narrowed the search range to avoid overfitting. For example, in the HSC dataset (1,947 cells), we limited the range to 1–5; for the mouse erythroid maturation dataset, containing only progenitor and erythroid populations, the range was set to 1–10.

Users may adjust the upper bound of the K search when sample size (N) is small, and we suggest limiting K such that the expected per-expert sample size (approximately N/K) remains sufficiently large (e.g., typically 300–500 as an empirical baseline, subject to data quality), thereby reducing the risk of very small experts and improving kinetic stability.

The selected numbers of experts were as follows: 5 experts in the erythroid lineage of mouse gastrulation; 2 in human hematopoiesis (scNT-seq); 12 in developing mouse hippocampus; 10 in both embryonic E18 mouse brain and mouse placentation (Stereo-seq); and 14 in adult mouse brain spatial transcriptomics.

Supplementary Note 4: MinCUT Loss for Expert Assignment in gating network

To enhance graph partitioning into clusters, the model employs the MinCUT Loss, which consists of Cut Loss and Orthogonality Loss components. This dual-structure loss function optimizes cluster assignments by minimizing inter-cluster connections and maximizing intra-cluster cohesion, thereby promoting distinctiveness and orthogonality in cluster assignments:

$$\mathcal{L}_{MinCUT} = \mathcal{L}_C + \mathcal{L}_O$$

Cut Loss The Cut Loss aims to optimize the graph's partitioning by minimizing edges connecting different clusters, thus enhancing connectivity within clusters. It is computed as follows:

$$\mathcal{L}_C = 1 - \frac{\mathrm{Tr}(\mathbf{C}^\mathbf{T}\mathbf{S}\mathbf{C})}{\mathrm{Tr}(\mathbf{C}^\mathbf{T}\mathbf{D}\mathbf{C})}$$

Here, $\mathbf{S}$ represents the symmetric Laplacian normalized adjacency matrix of the graph, denoting node connections, and $\mathbf{D}$ is the diagonal degree matrix with $D_{ii} = \sum_j S_{ij}$, representing the total connections of each node. The numerator, $\mathrm{Tr}(\mathbf{C}^\mathbf{T}\mathbf{S}\mathbf{C})$, calculates the total edge weight within the same clusters, while the denominator normalizes this by the total degree of the nodes in each cluster, ensuring balance and independence from cluster size.

Orthogonality Loss The Orthogonality Loss ensures that cluster assignment vectors are distinct and orthogonal, which is crucial for reducing redundancy and enhancing cluster separation. It is expressed as:

$$\mathcal{L}_O = \|\frac{\mathbf{C}^{\mathbf{T}}\mathbf{C}}{\|\mathbf{C}^{\mathbf{T}}\mathbf{C}\|} - \frac{\mathbf{I_k}}{\sqrt{K}}\|$$

Here, $\mathbf{C} \in \mathbb{R}^{+I \times K}$ refers to the cluster matrix, $\mathbf{I_k}$ is the K dim identity matrix, and $\| \cdot \|$ denotes the Frobenius norm. This loss encourages orthogonality among the vectors of different clusters, minimizing overlap and maximizing the distinctiveness of each cluster.

Supplementary Note 5: Time Loss details

Time Loss Components To incorporate the time loss into the model, we use two main components: Local Smooth loss and Expert Consistency loss. The overall time loss $\mathcal{L}_{\text{Time}}$ is given by:

$$\mathcal{L}_{\text{Time}} = \mathcal{L}_{\text{Local Smooth}} + \mathcal{L}_{\text{Expert Consistency}}$$

Local Smooth Loss: This component ensures that cells close in the latent space (embedding space) are also close in predicted time, thereby maintaining temporal consistency in the learned embeddings. Inspired by t-distributed Stochastic Neighbor Embedding (t-SNE), the smooth loss is defined as:

$$\mathcal{L}_{\text{Local Smooth}} = \frac{1}{I|\mathcal{N}_i|} \sum_{i=1}^{I} \sum_{j \in N_i} p_{ij} \log\left(\frac{p_{ij}}{q_{ij}}\right)$$

where:

- I is the number of cells, $\mathcal{N}_i$ is neighbors of cell i based on Euclidean distance in embedding space, here we set $|\mathcal{N}_i| = 50$.

- p_{ij} represents the similarity between cells i and j in the high-dimensional latent space, computed using a Gaussian kernel:

$$p_{ij} = \frac{\exp\left(-\frac{\|\mathbf{z}_i - \mathbf{z}_j\|^2}{2\sigma^2}\right)}{\sum_{k \in \mathcal{N}_i} \exp\left(-\frac{\|\mathbf{z}_i - \mathbf{z}_k\|^2}{2\sigma^2}\right)}$$

  Here, $\mathbf{z}_i$ and $\mathbf{z}_j$ are the embeddings of cells i and j, and σ is a scaling parameter, here we set $\sigma =$.

- q_{ij} represents the similarity in the low-dimensional time space, computed using a Student's t-distribution:

$$q_{ij} = \frac{(1 + \|t_i - t_j\|^2)^{-1}}{\sum_{k \in \mathcal{N}_i} (1 + \|t_i - t_k\|^2)^{-1}}$$

  Here, t_i and t_j are the predicted time points for cells i and j.

The t-SNE-inspired smooth loss aligns the temporal smoothness with the proximity in the latent space by minimizing the Kullback-Leibler (KL) divergence between the high-dimensional and low-dimensional similarities. This loss is particularly applied during the Dynamic Systems Fitting stage, where a relatively steady latent space has been obtained, aiding in stable time learning.

Expert-consistency loss: To orient latent time consistently with transcriptional regulation, we require that, within each expert cluster, (i) cells annotated as *up-regulated* for a gene show increasing unspliced counts over time, whereas (ii) *down-regulated* cells exhibit the opposite trend. Formally, for cluster k we minimise

$$\mathcal{L}^{k}_{\text{Expert Consistency}} = 1 - \frac{1}{\|\mathbf{R}_k\|_1 \sqrt{G}} \sum_{g=1}^{G} R_{kg} \cdot \operatorname{corr}\big(\mathbf{t}_k,\, \mathbf{u}_{k,g}\big),$$

where $\mathbf{t}_k \in \mathbb{R}^{I_k}$ is the vector of latent times, $\mathbf{u}_{k,g}$ the unspliced counts for gene g, and $R_{kg} \in \{-1, 0, 1\}$ the average regulation sign

$$R_{kg} = \frac{1}{I_k} \sum_{i \in \mathcal{I}_k} R_{ig}, \qquad \mathbf{R}_k = (R_{k1}, \ldots, R_{kG}).$$

The normaliser $\|\mathbf{R}_k\|_1 \sqrt{G}$ makes the loss invariant to cluster size and gene number. Corr denotes Pearson correlations. The total correlation loss is

$$\mathcal{L}_{\text{Expert Consistency}} = \frac{1}{K} \sum_{k=1}^{K} \mathcal{L}^{k}_{\text{Expert Consistency}}.$$

This expert-consistency loss, together with the smoothness term, shapes the latent time axis, enabling STEER to recover coherent temporal trajectories that respect gene-expert specific regulation dynamics.

Adaptive regulation prior: The initial regulation matrix $\mathbf{R}_{\text{init}} \in \{-1, 0, +1\}^{I \times G}$ is derived from curvature trends computed within GAAE-inferred clusters. These clusters approximate coarse kinetic regimes and serve as initialization for expert grouping during training. As the MoE refines regime assignment, the regulation prior $\mathbf{R}$ remains fixed during warm-up, and becomes adaptive in the later training phase.

Supplementary Note 6: Time-guided Neighbor Selection used in kinetic fitting

STEER adopts a training strategy that uses local forward differences to estimate the velocities of unspliced ($v_{u,g}$) and spliced ($v_{s,g}$) mRNA. Neighbor selection is critical because the true future cell states are unknown, and selecting reverse neighbors would lead to incorrect directionality. Therefore, we base neighbor selection on the predicted time points, retaining only those neighbors whose time points are later than that of the target cell. This process is essential for accurately capturing local kinetic variations.

To determine the neighborhood of target cell i for each gene g, we first use the k-nearest neighbors based on Euclidean distance to establish the initial neighbor set $\mathcal{N}^0_{i,g}$. The time-guided neighbor set $\mathcal{N}_{i,g}$ is then defined as:

$$\mathcal{N}_{i,g} = \big\{ j \mid t_j > t_i, \forall j \in \mathcal{N}^0_{i,g} \big\}$$

where $\mathcal{N}_{i,g}^0$ represents the initial set of neighbors for cell i, and t_j and t_i are the predicted time points for cells j and i, respectively. The $\mathcal{N}_{i,g}$ will be updated during training.

Supplementary Note 7: Simulations details

Kinetic framework All simulations are governed by the first-order RNA-velocity model

$$\frac{du}{dt} = \alpha - \beta u, \qquad \frac{ds}{dt} = \beta u - \gamma s,$$

where u and s are unspliced and spliced transcript counts. The transcription (α), splicing (β) and degradation (γ) rates are gene– and group–specific. For every gene in every group, the three rates were drawn independently from a uniform distribution whose bounds lie $\pm 10\,\%$ around the pattern-specific mean listed in Supplementary Table 3, e.g. $\alpha \sim \mathcal{U}(0.9\,\bar{\alpha},\ 1.1\,\bar{\alpha})$. The coupled ordinary differential equations were solved with an adaptive Runge–Kutta–Fehlberg integrator (RK45); step sizes were chosen automatically. Gaussian measurement noise was added to both u and s to emulate technical variability.

Overall-scheme simulation A single heterogeneous dataset was generated by embedding four canonical kinetic patterns within a branching manifold composed of one progenitor group (G_0) and two descendants (G_1, G_2).

Cell sampling

- $\mathbf{G}_0$ — 1 000 cells taken at equally spaced kinetic time points from $t = 0$ to $t = 2$.
- $\mathbf{G}_1$ and $\mathbf{G}_2$ — 500 cells each, equally spaced from $t = 2$ to $t = 5$.

Kinetic patterns

1. **Sequential shifts** — descendants share a common parameter set distinct from the progenitor, modelling monotonic maturation.
2. **Branching trajectories** — each descendant acquires its own parameter set after bifurcation, representing divergent lineage commitment.
3. **Multiple independent kinetics** — every group receives a unique parameter set; initial u and s values are zero for each group, reflecting de-novo regulation.
4. **Classic circular pattern** — genes are in an open phase ($\alpha > 0$) in G_0 and a closed phase ($\alpha = 0$) in both descendants, capturing induction–repression cycles.

For patterns 1, 2 and 4 the initial u and s values of each descendant were set to the terminal values of the progenitor trajectory, ensuring continuity along the manifold. For pattern 3 each group was initialised at $u = s = 0$.

Gene sampling strategy To preserve the intended kinetic topology while providing sufficient statistical power, we generated a four-gene unit—one gene per kinetic pattern—and replicated this unit 250 times. In every replication, α, β and γ for each gene were resampled independently from the pattern-specific distributions, yielding 250 statistically independent realisations per pattern.

The final dataset contains 2 000 cells and 1 000 genes and is produced deterministically from a single random seed; complete code is available in the STEER repository (see Code availability).

This consolidated overall-scheme integrates branching topology, group-specific rate heterogeneity and mixed cyclic/monotonic gene programmes in one dataset, offering a stringent yet interpretable benchmark for RNA-velocity algorithms.

Supplementary Note 8: Empirical observability of regime-specific kinetics in unspliced–spliced phase portraits

To provide direct, data-level evidence that distinct kinetic regimes are observable in real datasets, we examined unspliced–spliced (u,s) phase portraits for representative genes, with cells colored by STEER-inferred experts (Supplementary Fig.4c,d for erythroid maturation; Supplementary Fig.5d for dentate gyrus neurogenesis). These phase portraits visualize transcriptional dynamics in the raw (u,s) space and illustrate characteristic structures that are difficult to reconcile with a single globally shared kinetic relationship across all cells. We focus on two representative scenarios that commonly challenge global-kinetics fits: rate shifts along maturation and lineage branching.

Rate-shift (multi-rate) kinetics during erythroid maturation. Several erythroid "transcriptional boosting" genes reported previously[4] (e.g., *Hba-x*, *Smim1*, *Cpox*, *Fech*, *Hbb-y*) exhibit phase-portrait structures consistent with a stage-dependent change in effective kinetics. In Supplementary Fig.4c,d, late-stage cells occupy a terminal region of (u,s) space that follows a markedly steeper upregulation trend than earlier-stage cells, suggesting that a single global parameterization may not capture both the early and late segments of the trajectory simultaneously. Consistently, STEER assigns late-stage cells to distinct experts and recovers elevated transcription rates (α) in these terminal regimes, aligning with the expected late-maturation transcriptional boost.

Multi-branch kinetics in a branching neurodevelopmental system. In the dentate gyrus dataset, lineage branching gives rise to coexisting dynamical programs. For genes such as *Cntn1* and *Dscam*, the phase portraits in Supplementary Fig.5d display branch-structured patterns in (u,s) space (i.e., more than a single up-branch paired with a single down-branch), indicating that a single globally shared kinetic curve is unlikely to capture the full set of observed trajectories. STEER captures these branch-specific patterns by routing cells into different experts that correspond to distinct lineage-associated regimes, yielding expert-resolved velocity fields along each branch. For genes that change more monotonically along neuronal progression (e.g., *Rfx3*), the phase portraits likewise show expert-stratified structure consistent with smoothly varying kinetics across maturation.

Interpretation of expert stratification. Finally, we note that STEER's experts form a piecewise partition of the learned latent state–time space (latent representation space) designed to

reduce parameter interference while preserving global temporal continuity. Consequently, adjacent experts may correspond to consecutive segments of a broader dynamical progression and can exhibit similar (u,s) structures, which is expected under continuous dynamics rather than implying over-discretization. Together, these expert-stratified phase portraits provide direct empirical support that regime-dependent kinetics are observable in real biological datasets and motivate modeling frameworks that relax globally shared kinetic assumptions.

Supplementary Note 9: Downsampling analysis in linear developmental trajectories

To evaluate the robustness of STEER under temporally sparse sampling, we conducted systematic downsampling experiments on the mouse erythroid maturation dataset, which features a largely linear developmental trajectory. Selective removal of early or late time points led to noticeable declines in performance, whereas omission of intermediate stages had minimal effect (Supplementary Fig.9). Remarkably, training STEER on a reduced subset containing only one representative time point per cell type still yielded accurate estimates of latent kinetic time and transcriptional parameters, and generalized reliably to held-out cells (Supplementary Fig.10). These findings suggest that STEER effectively leverages global trajectory structure and requires only a minimal set of well-positioned temporal anchors to reconstruct coherent dynamic programs.

Supplementary Note 10: Branch-specific kinetic regimes in hippocampal neurogenesis inferred by STEER

We further evaluated STEER on the hippocampal neurogenesis dataset[1], where radial glia branch into astrocytes, oligodendrocyte precursor cells (OPCs), and diverse neuronal states. STEER partitioned the population into expert-defined kinetic regimes that align with major branches and capture branch-specific kinetics (Supplementary Fig.5a-c). For instance, an OPC-associated regime was characterized by elevated expression of canonical OPC markers (e.g., *Pdgfra*, *Cntn1*, *Dscam*), whereas an astrocyte-associated regime showed enrichment of astroglial markers (e.g., *Hapln1*) (Supplementary Fig.5c-d).

Within the neuronal branch, STEER identified a sequence of experts that are contiguous in kinetic-parameter space, consistent with progressive maturation from neuroblast-like states to dentate-gyrus granule neurons (Supplementary Fig.5c,e). Representative phase portraits further illustrate smooth expert-resolved velocity patterns for both upregulated and downregulated genes along this trajectory (Supplementary Fig.5c-e).

Supplementary Note 11: Robustness to expert number and effect of MinCUT regularization

The expert number K is selected using an entropy–elbow criterion. To assess robustness, we varied K around the selected value in a synthetic system with known kinetic regimes and ground-truth dynamics. STEER yields stable velocity fields and latent-time estimates for values of K near the selected optimum, with noticeable degradation only under substantial under-specification (Supplementary Fig.28), indicating limited sensitivity to moderate mis-specification of K.

We further examined the effect of MinCUT regularization on expert assignments. Removing MinCUT results in more diffuse and overlapping expert responsibilities, whereas its inclusion yields more concentrated and more interpretable regime assignments without affecting velocity accuracy (Supplementary Fig.28), supporting its role in promoting coherent and non-redundant kinetic specialization.

Supplementary Note 12: Implementation Details for Differential Kinetic Gene Identification

To identify Differential Kinetic Genes (DKGs) as described in the Methods section, we performed differential analysis on STEER-inferred kinetic parameters, including transcription (α), splicing (β), and degradation (γ) rates.

STEER infers cell-specific kinetic parameters for each gene. For each kinetic parameter layer (α, β, and γ), we compared the distributions of inferred rates between expert defined cell groups using the differential testing framework implemented in the Scanpy toolkit. Specifically, we applied the *rank_genes_groups* function with a standard t-test to assess whether a given kinetic parameter differed significantly between expert groups.

A gene was considered to exhibit a significant difference in a given kinetic parameter if it satisfied both of the following criteria: (i) p-value < 0.05, and (ii) an absolute $\log_2$ fold change > 1 (corresponding to a fold change greater than two). Genes meeting these criteria in at least one kinetic parameter were designated as DKGs.

DKGs were further classified according to the number of kinetic parameters showing significant differences between expert groups. Genes with one differential parameter were classified as single differential rate (SDR), those with two as double differential rate (DDR), and those with all three as triple differential rate (TDR). For DDR genes, we additionally distinguished coordinated (Sync) versus uncoordinated (Async) rate combinations based on their dynamical consequences: coordinated modulation of transcription and degradation acting in the same effective direction on mRNA abundance ($\uparrow\alpha$ with $\downarrow\gamma$, or $\downarrow\alpha$ with $\uparrow\gamma$) was classified as Sync, whereas all other combinations were classified as Async.

Supplementary Note 13: Computational cost and scalability

We benchmarked the computational cost of STEER on simulated spatial transcriptomics data and report end-to-end wall-clock runtime (including preprocessing, training, and inference) and peak GPU memory, together with empirical scaling trends as a function of the number of cells (N), velocity genes (G), and spatial neighbors (k) (Supplementary Fig.29). All benchmarks use the same model configuration as in our simulation evaluations; the number of experts is selected by the entropy-based criterion (Supplementary Note 3) and is $K = 3$ in Supplementary Fig.29. Specifically, STEER uses a GAAE encoder/decoder with a 512-dimensional hidden layer and a 128-dimensional latent embedding, and each expert is a three-layer MLP with hidden widths 256/512/1024 predicting gene-wise kinetics.

All methods were executed using the default pipelines and recommended hyperparameters provided in their public GitHub repositories. GPU-based methods were run on a single NVIDIA L20 GPU (48 GB). Peak GPU memory was recorded as the maximum allocated GPU memory during execution; STT is CPU-only and is therefore marked as not applicable (NA) for GPU memory. As a representative reference point, for $N = 10{,}000$ and $G = 500$, STEER completes the end-to-end

pipeline in < 15 minutes with peak GPU memory < 7 GB (Supplementary Fig.29). Across the tested settings, wall-clock runtime and peak memory increase monotonically with N, G, and k (Supplementary Fig.29), providing transparent guidance on resource requirements for spatial RNA velocity analysis.

For the scalability benchmarks in Supplementary Fig.29, we used a fixed training budget and early-stopping configuration across all tested settings to enable consistent runtime/memory comparisons: `pretrain_epochs`=500, `cluster_epochs`=200, `MIN_IMPRO`=0.01, and early stopping with `PATIENCE`=100. In practical applications, users may increase the training budget and early-stopping patience for more complex or noisier datasets (e.g., `PATIENCE`=300-1000 and proportionally larger `pretrain_epochs`/`cluster_epochs`). The exact configuration and scripts used to reproduce Supplementary Fig.29 are provided in the GitHub repository.

Supplementary Note 14: Quantification of expert boundary sharpness

To assess whether expert boundaries reflect kinetics-specific transitions beyond transcriptomic differences, we quantified boundary sharpness by comparing local shifts in inferred kinetic parameters and gene expression profiles between neighboring cells.

We first constructed a k-nearest-neighbor (kNN) graph ($k = 30$) in transcriptomic PCA space to ensure that all comparisons are restricted to locally similar cells in expression space. Based on this graph, we defined two classes of neighboring cell pairs: (i) *boundary pairs*, consisting of adjacent cells assigned to different experts, and (ii) *internal pairs*, consisting of adjacent cells assigned to the same expert.

For each pair, we computed an expression distance (Euclidean distance in PCA space) and a kinetic distance (Euclidean distance in the inferred per-cell (α, β, γ) space). To obtain scale-independent measures and control for local variability, boundary-pair distances were normalized by the mean internal-pair distance within the same region, yielding a relative jump magnitude (i.e., fold-change over the internal baseline).

For boundary pairs, we then compared the relative jump magnitude in kinetic space versus transcriptomic space using two-sided Mann–Whitney U tests. Across multiple regions (including the RG/Astro/OPC compartment and the V-SVZ example discussed in the main text), boundary interfaces exhibit significantly larger normalized transitions in kinetic space than in expression space (Supplementary Fig.14c).

These results indicate that expert boundaries are marked by kinetics-specific discontinuities that are not explained by local transcriptomic separation alone, supporting the interpretation that STEER experts capture regime-level dynamical organization rather than higher-resolution clustering of expression states.

Supplementary Note 15: Data Requirements and Robustness

To clarify practical data requirements, we evaluated STEER's robustness to reduced read coverage using controlled binomial downsampling calibrated to real-world spatial transcriptomics (ST) regimes (Supplementary Fig.11). Across downsampling levels, STEER preserved global temporal ordering and the projected low-dimensional (2D) velocity field on the embedding, whereas gene-resolved kinetic estimates degraded progressively with increasing sparsity, consistent with fundamental information limits under intronic depletion.

As an empirical reference, using a high-depth 10x Genomics v2 dentate gyrus dataset as a base-

line, STEER retained reliable trajectory directionality and latent-time ordering even when effective coverage was reduced to ∼20–25% of the reference depth (Supplementary Fig.11). This depth range overlaps with challenging yet common single-cell–resolution ST settings (e.g., Slide-seq and cell-bin Stereo-seq), where spliced signal can be relatively preserved while unspliced counts are sparse. Together, these results underscore a practical distinction between robust global dynamical inference (latent time and projected velocities) and more coverage-sensitive gene-wise kinetic interpretation, providing guidance for interpreting STEER outputs across diverse single-cell and spatial platforms.

References

[1] La Manno, Gioele and Soldatov, Ruslan and Zeisel, Amit and others. RNA velocity of single cells. *Nature*, 560(7719):494–498, 2018.

[2] Bergen, Volker and Lange, Moritz and others. Generalizing RNA velocity to transient cell states through dynamical modeling. *Nature Biotechnology*, 38(12):1408–1414, 2020.

[3] Baudry, Jean-Patrick and Raftery, Adrian E and Celeux, Gilles and Lo, Kenneth and Gottardo, Raphael. Combining mixture components for clustering. *Journal of computational and graphical statistics*, 19(2):332–353, 2010.

[4] Barile, Melania and Imaz-Rosshandler, Ivan and Inzani, Isabella and Ghazanfar, Shila and Nichols, Jennifer and Marioni, John C and Guibentif, Carolina and Göttgens, Berthold. Coordinated changes in gene expression kinetics underlie both mouse and human erythroid maturation. *Genome Biology*, 22:1–22, 2021.
